# How a patient advocacy group developed the first proposed draft guidance document for industry for submission to the U.S. Food and Drug Administration

**DOI:** 10.1186/s13023-015-0281-2

**Published:** 2015-06-24

**Authors:** Pat Furlong, John F. P. Bridges, Lawrence Charnas, Justin R. Fallon, Ryan Fischer, Kevin M. Flanigan, Timothy R. Franson, Neera Gulati, Craig McDonald, Holly Peay, H. Lee Sweeney

**Affiliations:** Parent Project Muscular Dystrophy, 401 Hackensack Avenue, 9th Floor, Hackensack, NJ 07601 New Jersey; Johns Hopkins Bloomberg School of Public Health, 624 N. Broadway, Rm 689, Baltimore, MD 21205 USA; Shire Plc, 300 Shire Way, Lexington, MA 02421 USA; Department of Neuroscience, Brown University, 185 Meeting Street, Box GL-N, Providence, RI 02912 USA; Nationwide Children’s Hospital, 700 N. Children’s Drive, Columbus, OH 43205 USA; YourEncore, 111 Monument Circle–Suite 1022, Indianapolis, IN 46204 USA; Suneel’s Light Foundation, 5651 Main Street, Suite 8-152, Williamsville, New York, 14221 USA; UC Davis NeuroNEXT, 4860 Y Street, Suite 3850, Sacramento, CA 95817 USA; University of Pennsylvania Perelman School of Medicine, Richards Bldg., Rm. B400, 3700 Hamilton Walk, Philadelphia, PA 19104 USA

**Keywords:** FDA, Industry guidance, Duchenne muscular dystrophy, Public policy, Advocacy, Rare disease, Clinical trial, Patient engagement, Drug development

## Abstract

Among the challenges confronting patients with rare diseases is a dearth of treatment options. The development of safe and effective new therapies is hampered by challenges associated with conducting clinical trials in small populations. In this article, we describe how the Duchenne muscular dystrophy community–led by Parent Project Muscular Dystrophy–created a proposed draft guidance document for industry for submission to the U.S. Food and Drug Administration. This unprecedented undertaking involved a broad coalition of more than 80 stakeholders collaborating across nine time zones to produce a document in only 6 months. We hope that other rare disease communities and advocacy organizations can use our experience as a model for developing their own draft guidance documents.

**Electronic supplementary material:**

The online version of this article (doi:10.1186/s13023-015-0281-2) contains supplementary material, which is available to authorized users.

## Introduction

Duchenne muscular dystrophy (Duchenne) is a genetic disorder characterized by progressive muscle weakness and degeneration [1–4]. It is caused by a mutation in the gene that encodes dystrophin, a protein critical to muscle integrity [1, 2, 4]. Boys are affected almost exclusively, with an estimated incidence of approximately 1 in every 4000 live male births [2, 4, 5].

Duchenne is typically diagnosed between 3 and 5 years of age, following the onset of symptoms such as delayed walking, difficulty climbing stairs, frequent falls, and difficulty running and jumping [1, 2, 4]. More than 90 % of patients require the use of a wheelchair by their mid-teens [5]. Weakened heart and lung muscles lead to various cardiac and pulmonary complications (e.g., cardiomyopathy, difficulty breathing, respiratory infections) [2, 4]. Affected individuals may also have varying degrees of cognitive, behavioral, or language impairment [4].

Within the last 15 years, individuals with Duchenne did not survive much beyond their teen years [1, 2, 4]. With improvements in care, the mean lifespan has increased to more than 25 years, with some patients living into their 30s, 40s, or even 50s [1, 2, 4]. Corticosteroids are prescribed off-label to slow the decline in muscle strength and function; current treatment guidelines recommend their use in all qualified individuals [4]. No drug currently has an FDA-approved indication for Duchenne [6].

To help accelerate the development and review of potential therapies for Duchenne, the Parent Project Muscular Dystrophy (PPMD) spearheaded an effort to develop the first patient advocacy-initiated draft guidance for a rare disease. This report details the process used to write the guidance, which included a broad coalition of more than 80 stakeholders collaborating across nine time zones. It is our hope that other rare disease communities and advocacy organizations can use this model for developing their own draft guidances.

### Deciding to develop draft industry guidance

The decision to develop a draft industry guidance came after PPMD had been working for more than a decade to educate the FDA and other regulatory agencies about Duchenne and its catastrophic effects on both patients and families. Several events were pivotal in making this decision. In March 2013, the European Medicines Agency (EMA) issued a draft guideline for public comment on the clinical investigation of medicinal products for the treatment of Duchenne and Becker muscular dystrophy [7]. The community stakeholders believed this draft guideline did not adequately address the needs of the Duchenne community. In response, PPMD convened an expert advisory committee to develop and issue recommendations about how the U.S. Food and Drug Administration (FDA) could most effectively evaluate new therapies for Duchenne. The committee included leading voices in academia, industry, and patient advocacy. The resulting white paper, “Putting Patients First,” was released in April 2013 [8]. PPMD also held several meetings with the FDA about developing a guidance for Duchenne.

In July 2013, PPMD met with FDA staff that included Center for Drug Evaluation and Research director Janet Woodcock. The meeting was a continuation of ongoing efforts to provide the agency with relevant information and data that might encourage greater flexibility in the drug review process. One of the topics discussed was the possibility of a guidance for industry addressing drug development for Duchenne. The purpose of such a document would be to assist sponsors in the clinical development of drugs and address the FDA’s current thinking about various issues that arise at all stages of product development, including the types of clinical trials that could be used to demonstrate efficacy and safety. Guidance documents traditionally have been authored by FDA staff.

During the discussion, the FDA staff acknowledged that the agency had neither the time nor resources to develop industry guidances for many of the rare diseases that might benefit, including Duchenne. As an alternative, FDA staff invited PPMD to submit a proposed draft guidance document, pursuant to language in the 2011 Good Guidance Practice (GGP) provisions [9]. A well-written proposed draft guidance could serve as the basis for–and hasten the development of–FDA’s own version of an industry guidance for Duchenne.

PPMD knew this invitation could not be declined. But given that no one outside FDA had ever developed a guidance document for industry, accepting the invitation meant entering uncharted territory. To begin navigating that terrain, PPMD organized a daylong policy forum modeled on a recently concluded scientific meeting in London. The policy forum, titled “Optimizing Clinical Trials in Duchenne Muscular Dystrophy in the New Era of Improved Care Standards: Guidance for the FDA,” was held in Silver Spring, Maryland, in December 2013. It was attended by more than 200 members of the Duchenne community, encompassing patients, parents, and representatives from the pharmaceutical and diagnostics industries, academia, clinical centers, and government. More than 20 FDA staff members (including Dr. Woodcock) also attended.

**Fig. 1:**
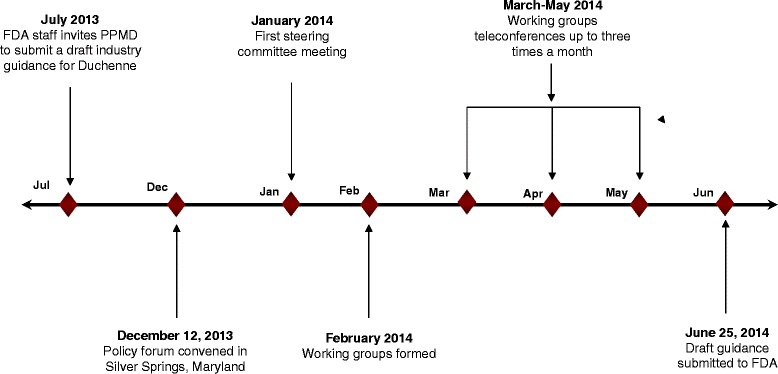
Draft guidance project timeline

The forum was structured as a mixture of scientific presentations, roundtable discussions, and testimony from patients, family members, and patient advocates, focused on (1) the challenges associated with designing and implementing clinical trials for rare diseases like Duchenne and (2) the need to accelerate approval of new therapies while meeting appropriate standards for safety and efficacy. It became evident during the course of the day that a draft industry guidance would require input from all of the key scientists and stakeholders–especially patients and Duchenne-affected families–to ensure that the final product would be both evidence-based and responsive to unmet community needs. The forum concluded with an agreement that the Duchenne community, led by PPMD, would work together to develop the proposed draft guidance. PPMD committed to completing the project in 6 months (see Fig. 1).

### Assembling the project team

**Fig. 2:**
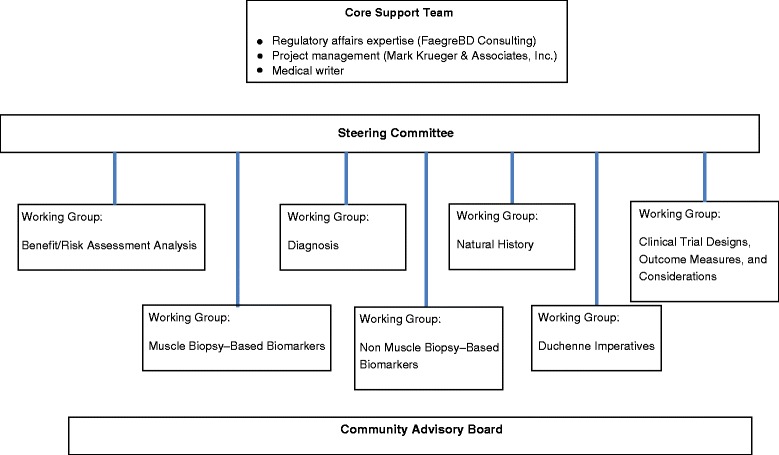
Entities involved in Developing the draft industry guidance

The aggressive timeline for completing the draft guidance necessitated a focused “divide and conquer” strategy, with content development for different parts of the document proceeding simultaneously. The strategy encompassed a core support team, a steering committee, seven expert working groups, and a community advisory board (Fig. 2).

#### Core support team

The core support team provided technical expertise and managed the many moving parts of guidance development. FaegreBD Consulting, a national firm with extensive experience in regulatory affairs, provided insight into working with the FDA and ensured that the proposed draft guidance document would be in the proper format and use appropriate terminology. Mark Krueger & Associates, Inc. (MK&A), a firm specializing in health care constituency relations, provided general project management services that included defining the scope and goals of the project, outlining participants’ responsibilities, organizing meetings of the steering committee and working groups, and ensuring the quality of all group discussions and work products. A medical writer with experience in treatment activism participated in all steering committee and working group meetings and created text for the evolving document.

#### Steering committee

The steering committee provided overall strategic direction for the guidance development process, setting the tone and ensuring that all perspectives from across the Duchenne community were represented. The committee consisted initially of six members: two parent/patient advocates, a pharmaceutical industry representative, a consultant who specialized in FDA policy, and two members of PPMD’s Scientific Advisory Committee. It was expanded to include all working group chairs who were not already part of the steering committee, bringing the total to 10.

#### Expert working groups

Working groups were responsible for assembling, analyzing, and reporting on the body of evidence related to a specific section of the draft guidance. To keep discussions manageable and increase the likelihood of meeting deadlines, most working groups were limited to six to eight members. All working groups had to include patient advocates, pharmaceutical industry representatives, and Duchenne experts (e.g., physicians, basic science researchers, epidemiologists). No more than three members of a working group could come from the pharmaceutical industry, and all were required to have appropriate and relevant scientific, clinical, or public health experience.

The chair of each working group also was a member of the steering committee and served as a liaison between the working group members and the steering committee. Chairs were permitted to invite non-members to participate if their specialized knowledge would illuminate specific discussions (e.g., an expert in clinical trial design invited to discuss outcome measures for pulmonary disease).

#### Community advisory board

Although Duchenne patients, parents, and patient representatives were included as members of the steering committee and all working groups, PPMD believed strongly that the draft guidance must represent the diverse needs and perspectives of the Duchenne community as much as possible. The organization solicited participants for a community advisory board through multiple avenues, including extending an invitation to community members who had attended the December 2013 policy forum, posting an open invitation on the PPMD website, sending targeted e-mail messages, and following up with telephone and face-to-face meetings. The community advisory board ultimately included 40 volunteers who reviewed the draft guidance with an eye toward ensuring that the patient voice was evident. The members of the Community Advisory Board are listed in Additional file 1.

### Establishing the vision

The steering committee met for the first time via teleconference on January 24, 2014. The members tackled an ambitious agenda:

- Articulate the goals, objectives, and framework of the draft guidance.
- Define topics to be addressed by working groups and develop charges for each group.
- Outline the roles and responsibilities of working group members and identify possible members for each group.

To determine the most appropriate length and format for the draft guidance, MK&A identified six representative FDA guidances in five therapeutic areas: Alzheimer’s disease, human immunodeficiency virus (HIV), community-acquired pneumonia, irritable bowel syndrome, and systemic lupus erythematosus. A side-by-side comparison revealed an average of 15 pages devoted to actual content (range 6–27 pages). Overlapping or common sections included an introduction, background, general considerations, specific efficacy trial considerations, references, and one or more appendixes. The steering committee decided to aim for a document that would include those sections and have 20 to 25 pages of actual content.

The steering committee identified seven theme areas to be included in the Duchenne draft guidance and addressed by working groups. The theme areas of each working group are detailed below:

#### Benefit/risk assessment analysis

This section would reflect the Duchenne community’s tolerance of either potential risks or uncertainty of benefit associated with new treatment options. Benefit/risk assessments typically are discussed in a few paragraphs towards the end of guidance documents. However, the steering committee felt that the benefit/risk ratio and patient/caregiver preferences deserved more in-depth consideration and positioning early in the Duchenne draft guidance. The benefit-risk framework is the fundamental structure under which the FDA makes regulatory decisions; putting it at the front of the document would provide an overarching focus for the remainder of the guidance. The steering committee wanted to encourage clinical trial sponsors to work with patients and parents to understand patient preferences at the very outset and throughout the clinical development of potential new therapies. Also, PPMD already had invested in research to determine treatment preferences and risk tolerance in Duchenne [10]; the results would enable FDA to rely more heavily on data regarding the community’s preferences than on public testimony and anecdotes gathered during New Drug Application hearings.

#### Diagnosis

This section would communicate problems associated with the diagnostic delay in Duchenne and the importance of genetic analysis using modern technologies, including access to genetic testing. It also would address the continued importance of clinical assessments in the diagnosis and staging of Duchenne.

#### Natural history

This section would feature a more detailed description of natural history than usually appears in guidance documents. The steering committee wanted to update sponsors and regulators on the large body of recent quality data that more accurately characterized the variable clinical course of Duchenne, to encourage use of the information for trial design as well as for non-concurrent controls in novel trial designs. Specific attention would be paid to the timing of loss of certain functional abilities with current medical management, including how Duchenne affects cardiac and pulmonary function.

#### Clinical trial designs, outcome measures and considerations

This section would focus on how best to evaluate new therapies to bring them to market efficiently, while calling on sponsors to design trials that include Duchenne patients of all ages and disease stages to the extent possible. At first, this theme was intended to be addressed by two working groups: one on outcome measures and one on clinical trials designs. The steering committee decided on a single working group when members realized how inter-related the topics were.

#### Biomarkers

This section would address the large and controversial topic of biomarkers in clinical trials of possible Duchenne therapies. The steering committee did create two working groups for this theme:

##### Muscle biopsy-based biomarkers

This working group focused on the use of biomarkers based on mutations in the dystrophin gene, quantification of dystrophin and muscle biopsy.

##### Non-muscle biopsy-based biomarkers

This working group explored emerging noninvasive techniques such as magnetic resonance imaging and spectroscopy (MRI/MRS).

#### Duchenne imperatives

This theme was not intended or expected to be a specific section in the Duchenne draft guidance. Rather, it was intended to strengthen the patient voice throughout the document. Several key community imperatives had been identified during the policy forum, including the use of placebo versus natural history controls in clinical trials, the narrow eligibility criteria of clinical trials, and controversies surrounding the use of dystrophin as an endpoint in clinical trials. The steering committee wanted a mechanism for the community to clearly voice its position on those key issues and other matters raised during the guidance development process.

### Developing the content

With the theme areas established, the steering committee formalized the charges for the working groups and identified possible members. Some steering committee members were designated as working group chairs; the other chairs were selected from the list of possible group members. The working group chairs were responsible for contacting and inviting the members of their respective groups. The steering committee and working group members are listed in Additional file 2.

The steering committee continued to meet monthly via teleconference or in person through May 2014. The two in-person meetings were scheduled to take advantage of member attendance at national conferences.

**Fig. 3:**
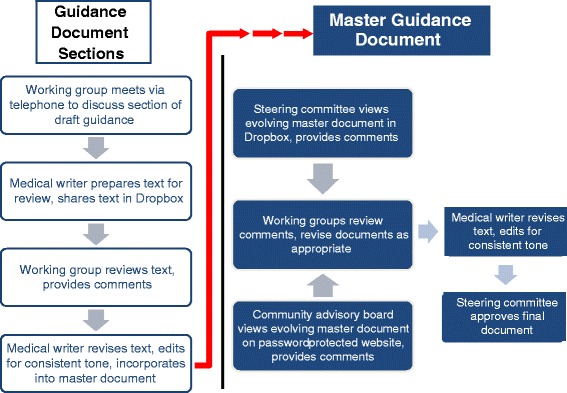
Process for developing individual draft guidance document sections and master document

Working groups met via telephone for 60 to 90 min once or twice each month (depending on the topic) from March to May (see Fig. 3). Before the first meeting, the chairperson of each working group collaborated with the medical writer on a detailed table of contents for the draft guidance. This outline helped to ensure minimal overlap or redundancy among sections; it also produced a de facto discussion guide that working group members used to focus and direct the conversation during meetings.

After each working group call, the medical writer created text based on the discussion and deposited the document in a shared Dropbox folder for member review and comment. As text was finalized, the medical writer edited it to ensure consistency of tone and added it to the working version of the draft guidance.

All working groups operated under a set of guiding principles established by the steering committee. The guidance document needed to be fair and balanced, incorporating and referencing the best available evidence while also representing the needs of patients. The process needed to be transparent, with ample opportunity for community commentary on both the direction and content. The guidance needed to be forward thinking, enabling industry to expedite the development of potential new therapies that meet high standards for safety and efficacy. It also had to incorporate the patient voice and encourage the use of established FDA policies to accelerate the approval of drugs that would increase the lifespan and improve the quality of life of patients with Duchenne.

In keeping with these guiding principles, free and open discussion of both published literature and current thinking was encouraged. Leading Duchenne experts who participated on working groups often were aware of research in progress or data that had been presented at scientific meetings but not published yet. Rules had to be established for what evidence could be included in the guidance. Steering committee members agreed that papers in peer-reviewed journals or in press could be cited. Other important papers that had been submitted to medical journals and were known to be in a late stage of review also could be cited, but they had to be marked clearly as unpublished data.

Part of MK&A’s role was to mediate and adjudicate conflicting views among steering committee and working group members when necessary. The steering committee wanted to avoid a situation in which a minority of participants disagreed with the direction the guidance document was taking and disassociated themselves from the project (or created an alternative guidance). Everyone involved in the process would have to stand behind the final product, acknowledging instances in which the literature and expert opinion did not necessarily converge.

The community advisory board met via teleconference or webinar in March, April, and May 2013 to review and comment on materials in development. The most current versions were posted on a password-protected page on the PPMD website. After each meeting, the medical writer compiled the comments and shared them with the working groups. Each working group determined how to address and incorporate comments relevant to its section.

As the complete guidance document took shape, the medical writer collaborated with the working group chairs on any sections deemed to need additional information or clarification. The penultimate version was shared with all steering committee and working group members for a last round of review. Once all comments were incorporated to everyone’s satisfaction, the steering committee approved the document.

### Finalizing the draft guidance

After nearly 50 revisions, the final version of the proposed draft guidance for industry–titled “Duchenne Muscular Dystrophy: Developing Drugs for Treatment over the Spectrum of Disease”–was submitted to the FDA on June 26, 2014 (Additional file 3). At 47 pages of content, the document was more than double the original target length.

The Duchenne Imperatives Working Group crafted a cover letter for the guidance that reiterated the urgency felt by patients and families, summarized the guidance content, and highlighted the community’s most critical requests (Additional file 4). These requests included some advocacy imperatives that did not fit or appear in the guidance, such as a call to implement routine newborn screening for Duchenne.

At the request of the community representative on the steering committee, the cover letter included personal stories from several parents. These stories recounted the heartbreak associated with watching a child’s progressive loss of function and independence and increasing dependence on family, as well as the extraordinary burdens–physical, financial, emotional, and spiritual–that Duchenne places on families and caregivers.

At the time this manuscript was prepared, it remained unclear how much of the proposed draft guidance would be included in FDA’s own version. The FDA accepted the submission and held an internal meeting on August 13, 2014 to review the document. A required 30-day public comment period began on September 4, 2014, via an open docket. Recent conversations between PPMD and the FDA suggest the FDA will release the FDA’s guidance for Duchenne by the end of 2015.

### Publishing the draft guidance

From the start of the process, the steering committee stressed the importance of publishing PPMD’s proposed draft guidance document in its entirety (including the cover letter, (Additional files 3 and 4) in a medical, scientific, or policy journal. Doing so would share the views of the working groups with the entire Duchenne community and present them to other rare disease groups for review. In addition, the steering committee felt that publishing PPMD’s guidance document would provide further encouragement for the FDA to integrate as many of PPMD’s recommendations into the FDA’s own guidance for industry on Duchenne.

PPMD views its submitted guidance for industry as a living document, to be updated as new data are published and new therapies enter the market. A formal process for accomplishing this has not been established yet. However, PPMD has acted opportunistically to provide additional input on pressing clinical challenges in Duchenne management. For example, PPMD collaborated with the National Institutes of Health (NIH) National Heart, Lung and Blood Institute (NHLBI) to convene a working group meeting in July 2014 that explored current issues relevant to cardiac disease in patients with Duchenne. Attendees included experts in cardiology, clinical trials, patient advocacy, and FDA. A discussion at that meeting led to questions about the optimal cardiac surveillance practices to include in Duchenne clinical trials. The working group developed a position paper [11] covering responses to those questions, including recommendations for cardiac surveillance in Duchenne clinical trials, and PPMD submitted it to the FDA for inclusion as an addendum to the draft guidance [12].

### Benefits to the Duchenne community

Working together to produce the guidance resulted in a stronger relationship between the Duchenne community, sponsors and the FDA. The quality of the document produced enhanced the credibility and relationship between PPMD, the FDA, sponsors and the Duchenne community. Both the FDA and sponsors are now more interested in obtaining the patient perspective.

This is an extension of PPMD’s previous work to inform the FDA’s benefit-risk assessments with concrete data on treatment preferences and risk tolerance in Duchenne. PPMD conducted a rigorous survey [10] of more than 100 Duchenne caregivers. In the study, caregivers were presented with hypothetical treatment profiles and asked to judge which aspects they thought were best and worst. The results indicated that caregivers would be willing to accept the risk of a serious side effect if the treatment stopped or slowed the progression of muscle weakness, even absent improvement in lifespan [10]. This study informed the FDA’s benefit–risk assessments, and PPMD believes the FDA will give greater weight to the demonstrated benefit/risk preferences of patients and caregivers when assessing and making risk–benefit determinations in the future.

The relationship PPMD and the Duchenne community have established with the FDA has fostered additional collaborations to improve patient involvement in clinical outcome measures. As mentioned above, PPMD collaborated with NHLBI to explore issues relevant to cardiac disease in Duchenne patients [12]. This collaboration developed a position paper [11] that included recommendations for cardiac surveillance in Duchenne clinical trials. PPMD and the Duchenne community continue to encourage sponsors to work with patients to validate current patient reported outcome measures or develop new ones, to explore the use of exploratory outcome measures and to support the development of novel outcome measures. PPMD has been involved in determining how to identify outcome measures so ambulatory and non-ambulatory Duchenne patients can participate in clinical trials. Examples include the development of outcome measures for upper limb strength and fatigue. Upper limb strength can be measured with MRI, and measurements of reachable work space interacting with the Microsoft Kinect system. Fatigue protocols have been developed to quantify levels of fatigue. These new outcome measures allow sponsors to stratify patients for inclusion criteria in clinical trials. This collaboration enables sponsors to conduct clinical studies that include individuals across the spectrum of Duchenne disease.

### Implications, critical learnings and key success factors

Other groups seeking to replicate this approach to developing a draft guidance for industry need to be cognizant of the significant time and expense involved. PPMD entered the process with many advantages, including an established relationship with key opinion leaders, the FDA, and the resources needed to complete the project. The success of this project is also attributed to learning from other communities, hiring an independent project management company and medical writer. The focus on the guidance enabled all members of the Duchenne community to find common ground and speak with one voice. All of these considerations contributed to the success of this project and are detailed below.

#### Access to leading experts

PPMD has always been well connected to leading basic and clinical researchers through its medical and scientific advisory board and peer-review process for grant applications, among other routes. Leaders in the Duchenne field already had ties to the organization and respected its goals and programs. As a result, many were eager to contribute their knowledge and credibility and willing to accept the challenge of creating the draft guidance on a short timeline. In fact, PPMD had more volunteers than working group spots; many difficult decisions were made on the basis of availability during the very short development time frame.

#### Credibility with regulators

PPMD has demonstrated its command of complex challenges in drug development and approval through efforts such as “Putting Patients First” and the December 2013 public policy forum. FDA was willing to engage with PPMD in part because of the organization’s knowledge and rational approach, incorporating objective views and data wherever possible.

#### Sufficient staff and budget

Creating the proposed draft guidance took considerable time and patience. PPMD saw this document as a long-term commitment and made the decision, with board approval, to invest the necessary funds. It helped considerably that all steering committee members, working group members, and community advisors generously volunteered their time and effort. However, PPMD hired MK&A, an independent project management company, and medical writer, which added to the success of the project.

#### Learning from other communities

At the start of this process, PPMD reached out to other communities, such as the HIV community, with known experience in this area to provide guidance and advice during this process. The HIV community was willing to provide this information, which added to the success of the project.

#### MK&A, project management company

MK&A organized all of the logistics needed to produce the guidance in the short (6 month) timeframe. This included helping to define the scope and goals of the project and outlining participants’ responsibilities. MK&A also handled all of the timelines, phone calls with committee members, scheduled all of the meetings, prepared agendas, attended and wrote summaries of all meetings, provided coordination between groups and ensured the quality of all group discussions and work products. This enabled all volunteers and the staff at PPMD to focus on writing the guidance document.

#### Medical writer

The independent medical writer was provided content from all parts of the Duchenne community. This writer participated in all steering committee and working group meetings, reviewed the materials and objectively developed the content included in the guidance. The resulting document reflects the opinions of all members of the Duchenne community.

#### Consistent messaging

The Duchenne community focused around the guidance, with a collective voice and consistent messaging. This was an important way for all members of the Duchenne community to work together.

PPMD and the Duchenne community realize the urgency of Duchenne disease and wanted to develop the guidance as thoroughly and quickly as possible. This community, led by PPMD, wanted to produce a quality document that the FDA would be able to use as a draft. Hence, the draft guidance became the priority for the Duchenne community. Since submission of the guidance, PPMD has received positive feedback from the FDA.

### Drafting a guidance with insufficient time and expenses

PPMD notes that limited resources would have extended the time needed for this project. It is likely that some members of the Duchenne community may not have been willing or able to volunteer their time for longer. It is estimated that each volunteer spent at least 4 to 6 h per month for 6 months on this project. Thus, producing the guidance in the most efficient manner was of utmost importance for both the volunteers and patients.

### Recommendations for other patient advocacy groups

Several factors need to be in place before a patient advocacy group community should consider developing a draft guidance. First, the disease must be characterized. Areas of unmet need should be established and discussed with leading experts in the field, and the FDA. Working as a partner with the FDA, PPMD has conducted studies^10^ to provide the data needed to inform decisions. Developing these relationships as partners in the fight against Duchenne disease were critical to successfully developing this guidance.

## Conclusion

Developing a proposed draft guidance for industry may represent the best opportunity for a rare disease community to shape the way the FDA reviews new potential therapies in historically challenging rare disease categories. We hope that our experience and the model described in this article demonstrate that such an undertaking is both realistic and achievable.

## Additional files

Additional file 1:
**Members of Community Advisory Board.** List of community advisory members and the organizations involved in the development of the guidance. Additional file 2:
**Members of Steering Committee and Working Groups.** List of steering committee and working group members involved in the development of the guidance. Additional file 3:
**Guidance.** Draft guidance submitted to the FDA. Additional file 4:
**Duchenne Community Imperatives and Cover Letter.** Duchenne Community Imperatives and Cover Letter submitted with draft guidance to the FDA.

## Abbreviations

Duchenne: Duchenne muscular dystrophy
EMA: European Medicines Agency
FDA: U.S. Food and Drug Administration
GGP: Good Guidance Practice
HIV: Human immunodeficiency virus
MK&A: Mark Krueger and Associates, Inc.
MRI/MRS: Magnetic resonance imaging and spectroscopy
NDA: New Drug Application
NHLBI: National Heart, Lung and Blood Institute
NIH: National Institutes of Health
PPMD: Parent Project Muscular Dystrophy
