# Supplementary material for: How a patient advocacy group developed the first proposed draft guidance document for industry for submission to the U.S. Food and Drug Administration
Source: Orphanet J Rare Dis. 2015 Jun 24;10:82. doi: 10.1186/s13023-015-0281-2 (PMC4486430; doi:10.1186/s13023-015-0281-2)
Supplement: Additional file 1: — Members of Community Advisory Board. List of community advisory members and the organizations involved in the development of the guidance. [file 13023_2015_281_MOESM1_ESM.docx]

**Additional file 1**

**Draft Industry Guidance Community Advisory Board Members**

| **Foundation Representatives** | |
| --- | --- |
| **Name** | **Foundation** |
| Steve Dreher | Hope for Gus |
| Neera Gulati, MD | Suneel's Light |
| Cath Jayasuriya | Coalition Duchenne |
| Alex Johnson | Joining Jack |
| Jenn Mcnary | The Jett Foundation |
| Christine McSherry | The Jett Foundation |
| Robert Meadowcroft | Muscular Dystrophy Campaign |
| Debra Miller | CureDuchenne |
| Marissa Penrod | Team Joseph |
| Jen Portnoy | Hope for Javier |
| James Raffone | Jar of Hope |
| Diana Ribeiro | Action Duchenne |
| Tracy Seckler | Charley's Fund |
| Alex Smith | Harrison's Fund |
| Elizabeth Vroom | UPPMD |
| Allison Willis | Two Smiles One Hope Foundation |

| **Patient and Parent Representatives** |
| --- |
| **Name** |
| Elliot Barnett |
| Lynnette Bartels |
| Karen Burch |
| Sarah Burgess |
| Mindy Cameron |
| Catherine Collins |
| Brian Denger |
| Debbie Dupree |
| Terri Ellsworth |
| Anessa Fehsenfeld |
| Julie Garcia |
| Mohamed Haider |
| Joanna Johnson |
| Grace Lightcap |
| Elizabeth Longmire |
| Jessica May |
| Todd Morrow |
| Stan Nelson, MD, PhD |
| Christine Piacentino |
| Regina Reidenberg |
| Aparna Surampudi |
| Tayjus Surampudi |
| Ellen Wagner |
| Jeff Watkins |
