# Supplementary material for: How a patient advocacy group developed the first proposed draft guidance document for industry for submission to the U.S. Food and Drug Administration
Source: Orphanet J Rare Dis. 2015 Jun 24;10:82. doi: 10.1186/s13023-015-0281-2 (PMC4486430; doi:10.1186/s13023-015-0281-2)
Supplement: Additional file 2: — Members of Steering Committee and Working Groups. List of steering committee and working group members involved in the development of the guidance. [file 13023_2015_281_MOESM2_ESM.docx]

**Additional file 2**

**Draft Industry Guidance Steering Committee and Working Group Members**

***Steering Committee***

| **Expertise** | **Name** | **Affiliation** |
| --- | --- | --- |
| Academia | John Bridges, PhD | John Hopkins University |
| Industry | Lawrence Charnas, MD, PhD | Shire |
| Academia | Justin Fallon, PhD | Brown University |
| Science and Clinical Medicine | Kevin Flanigan, MD | Nationwide Children’s |
| Patient Advocacy | Pat Furlong | PPMD |
| Policy | Tim Franson, MD | YourEncore |
| Patient Advocacy | Neera Gulati, MD | Suneel’s Light Foundation |
| Science and Clinical Medicine | Craig McDonald, MD | UC Davis Health System |
| Science and Clinical Medicine | Lee Sweeney, PhD | University of Pennsylvania |

***Working Groups***

| **Name** | **Affiliation** |
| --- | --- |
| *Working Group 1: Benefit/Risk Assessment Analysis* | |
| John Bridges, PhD* | Johns Hopkins Bloomberg School of Public Health |
| Holly Peay, MS* | Parent Project Muscular Dystrophy |
| Erin Longley, MD | Parent/Patient advocate |
| Patrick Denger | Patient/Patient advocate |
| Susan DosReis | Department of Pharmacy, UMD |
| Richard Hermann, MD, MPH | AstraZeneca |
| Reed Johnson | RTI |
| Bennett Levitan | Johnson & Johnson |
| Marilyn Metcalf, PhD | GSK |
| Rebecca Noel, DrPH,MPH | Eli Lilly and Company |
| Ellen Wagner | Parent/Patient advocate |
| Eve Wittenberg, PhD | Center for Health Decision Science Harvard School of Public Health |
| *Working Group 2: Diagnosis* | |
| Kevin Flanigan, MD* | Nationwide Children's Hospital |
| Annemike Aartsma-Rus | Department of Human Genetics, Leiden UMC |
| Kate Haviland | Sarepta Therapeutics |
| Madhuri Hegde, PhD, FACMG | Emory University, Department of Human Genetics |
| Ann Martin, MS, CGC | DuchenneConnect |
| Kathy Mathews, MD | University of Iowa Hospitals & Clinics |
| Vanessa Rangel Miller, MS, CGC | PatientCrossroads |
| Todd Morrow | Parent/Patient advocate |
| Stan Nelson, MD, PhD | UCLA |
| Bob Weiss, PhD | University of Utah, Department of Human Genetics |
| *Working Group 3: Natural History* | |
| Craig McDonald, MD* | National Institute of Neurological Disorders and Stroke |
| Doug Biggar, MD | Holland Bloorview Kids Rehabilitation Hospital, University of Toronto |
| Katie Bushby, MD | Institute of Genetic Medicine at Newcastle University |
| Avital Cnaan, PhD | George Washington University, Children's National Medical Center |
| David Cox, PhD | Eli Lilly |
| Kevin Flanigan, MD | Nationwide Children's Hospital |
| Nathalie Goemans | Universitair Ziekenhuis Leuven |
| Mohamed Haider | Patient |
| Eugenio Mercuri | The Agostino Gemelli Teaching Hospital, Italy |
| Marrissa Penrod | Parent/Patient advocate |
| Allen Reha, MS | PTC Therapeutics |
| *Working Group 4: Muscle Biopsy-Based Biomarkers* | |
| Justin Fallon, PhD* | Brown University |
| Annemieke Aartsma-Rus | Department of Human Genetics of the LUMC |
| Mindy Cameron | Parent/Patient advocate |
| James Ervasti, PhD | University of Minnesota |
| Kevin Flanigan, MD | Nationwide Children's Hospital |
| Sharon Hesterlee, PhD | Parent Project Muscular Dystrophy |
| Eric Hoffman, PhD | Children's National Medical Center |
| Afrodite Lourbakos , PhD | Prosensa Therapeutics |
| Peter Sazani , PhD | Sarepta Therapeutics |
| *Working Group 5: Non Muscle Biopsy-Based Biomarkers* | |
| Lee Sweeney, PhD* | University of Pennsylvania |
| Terri Ellsworth | Parent/Patient advocate |
| Larry Gold, PhD | SomaLogic |
| Glen Nuckolls, PhD | National Institutes of Health (NIH) |
| Stu Peltz, PhD | PTC Therapeutics |
| Bill Rooney, PhD | Oregon Health & Science University |
| Krista Vandenborne,PhD | University of Florida |
| Glenn Walter, PhD | University of Florida |
| *Working Group 6: Clinical Trial Designs, Outcome Measures, and Considerations* | |
| Lawrence Charnas, MD, PhD* | Shire |
| Giles Campion, MD | Prosensa |
| Catherine Collins | Parent/Patient advocate |
| Anne Connolly, MD | Washington University School of Medicine |
| Linda Cripe, MD | Nationwide Children's Hospital |
| Nathalie Goemans, MD., PhD | Kinderneurologie-NMRC Kinderen |
| Ed Kaye, MD | Sarepta Therapeutics |
| Craig McDonald, MD | UC Davis Health System |
| Eugenio Mercuri, MD | The Agostino Gemelli Teaching Hospital, Italy |
| Elizabeth Vroom, DMD | Duchenne Parent Project Netherlands, UPPMD |
| *Working Group 7: Duchenne Imperatives* | |
| Pat Furlong* | Parent Project Muscular Dystrophy |
| Albert J. Allen, MD, PhD | Eli Lilly and Company |
| Margaret Anderson | Faster Cures |
| Steve Dreher | Parent/Patient advocate |
| Tim Franson, MD | FaegreBD Consulting |
| Stephen Groft | National Institutes of Health (NIH) |
| Marlene Haffner, MPH | Haffner Associates, LLC |
| Mohamed Haider | Patient |
| Julia Jenkins | EveryLife Foundation for Rare Diseases |
| Annie Kennedy | Muscular Dystrophy Association (MDA) |
| Kim McCleary | Faster Cures |
| Jeff Watkins | Parent/Patient advocate |

*Indicates committee chairs or co-chairs.
