# Supplementary material for: How a patient advocacy group developed the first proposed draft guidance document for industry for submission to the U.S. Food and Drug Administration
Source: Orphanet J Rare Dis. 2015 Jun 24;10:82. doi: 10.1186/s13023-015-0281-2 (PMC4486430; doi:10.1186/s13023-015-0281-2)
Supplement: Additional file 3: — Guidance. Draft guidance submitted to the FDA. [file 13023_2015_281_MOESM3_ESM.pdf]

Guidance for Industry
Duchenne Muscular Dystrophy Developing Drugs for Treatment over the Spectrum of Disease

TABLE OF CONTENTS

I. INTRODUCTION **1**
II. BACKGROUND **2**
III. BENEFIT/RISK ASSESSMENTS IN DUCHENNE MUSCULAR DYSTROPHY **4**
- **A. General Comments** **4**
- **B. FDA's Approach to Benefit/Risk Assessment** **5**
- **C. Incorporating Patient/Family Preferences** **6**
  - 1. Duchenne Case Study 6
- **D. Guidance to Sponsors** **7**
- **E. Conclusions and Special Considerations in Duchenne** **7**

IV. DIAGNOSTIC CRITERIA **8**
- **A. General Comments** **8**
  - 1. The DMD diagnostic odyssey 9
- **B. Diagnostic Laboratory Investigations/Methods for confirming diagnosis** **9**
  - 1. Genetic Analysis 9
    - a. Methodology 10
    - b. Access to genetic testing 10
  - 2. Dystrophin expression on muscle biopsy as a diagnostic biomarker 10
- **C. Newborn screening** **11**
- **D. The spectrum and clinical classification of dystrophinopathies** **11**

V. THE CURRENT UNDERSTANDING OF THE NATURAL HISTORY OF DUCHENNE MUSCULAR DYSTROPHY **11**
- **A. General Comments** **11**
- **B. Testing and evaluation across the spectrum of disease** **12**
- **C. Overview of the Natural History in Duchenne muscular dystrophy** **13**
- **D. How optimal medical management affects the course of DMD** ... **16**
  - 1. Glucocorticoid therapy 16
  - 2. Contracture management 17
  - 3. Spine deformity management 17
  - 4. Pulmonary management 17
  - 5. Cardiac management 17
- **E. Heterogeneity in DMD disease progression: Predictability and sources of variability** **18**
  - 1. Sources of variability 18
    - a. Disease severity / stage of disease 18
    - b. Genetic predictors of disease progression 19
    - c. Genetic modifiers 19
    - d. Corticosteroid therapy 20
    - e. Night splinting, physical therapy, and other standard interventions 20
- **F. Ongoing natural history study needs** **21**

VI. DMD CLINICAL TRIAL DESIGNS, OUTCOME MEASURES AND CONSIDERATIONS……21
- **A. General Comments……21**
- **B. Maximizing inclusion of populations in studies……23**
- **C. Clinical outcome measures and endpoints in DMD**
  - 1. Motor Outcome Measures……23
    - a. In neonates and infants……23
    - b. For young Ambulatory (from four to approximately seven)……24
    - c. The Late Ambulatory stage (from approximately seven to thirteen years of age)……25
    - d. The non-ambulant population……26
  - 2. Pulmonary Outcome Measures and Endpoints……27
  - 3. Cardiac Endpoints……28
  - 4. Patient Reported Outcomes (PROs) in DMD……29
  - 5. Development of Additional Novel Endpoints in DMD……29
- **D. Clinical Trials……30**
  - 1. Feasibility issues for trials in DMD: The limits of rare disease and medical addressability by endpoint (pool of participants and trial sites)……30
  - 2. Trial Design……31
    - a. Novel trial designs……32
  - 3. Standardization of measurement across trials……32
  - 4. Use of biomarkers in DMD trials……32
    - a. As Single Primary Outcome Measures……32
    - b. Use of Biomarkers as Supportive Secondary Outcome Measures……33
- **E. Extrapolation……33**

VII. BIOMARKERS IN DUCHENNE MUSCULAR DYSTROPHY……33
- **A. General Comments……33**
- **B. Muscle Biopsy Biomarkers: Dystrophin And Utrophin……35**
  - 1. General comments……35
  - 2. Considerations related to muscle biopsies……35
    - a. Ethical concerns of biopsies in children……35
    - b. Criteria of what is an appropriate biopsy for dystrophin or utrophin quantification……36
      - i. Site of biopsy/muscle group……36
      - ii. The handling of the biopsy……36
      - iii. Minimizing variability and sampling errors ….36
  - 3. Dystrophin Analyses……37
    - a. Broadly disseminated techniques……37
      - i. Immunofluorescence or immunohistochemical analysis by type……37
      - ii. Western Blot……38
    - b. Emerging technologies……38
      - i. Mass spectrometry……38

c. Current limitations for all methods……38
d. Reference ranges and outliers……39
e. Use of dystrophin quantification or relative quantification as a biochemical outcome measure……39
4. Utrophin Analysis……39
5. Muscle Biopsy Biomarkers: RT/RNA PCR analysis for exon-skipping detection to confirm mechanism of action in the exon-skipping field……40
C. Non-Biopsy Based Biomarkers……41
1. General Comments……41
2. Serum and Urine Biomarkers……41
a. Proteins and protein fragments……42
b. MicroRNAs……42
c. Recommendations regarding serum and urine biomarkers……43
3. Imaging Modalities……43
a. Ultrasound……43
b. Electrical impedance myography (EIM)……44
c. DEXA……44
d. MRI and MRS……44
i. MRI/MRS: Emerging Biomarkers of Human muscular dystrophy Pathology……45
ii. MR Imaging of Fibrosis……46
iii. MRI/MRS: Role in Clinical Trials……46
VIII. CONCLUSION……47

REFERENCES……48

Guidance for Industry
Duchenne Muscular Dystrophy
Developing Drugs for
Treatment over the Spectrum of Disease

This draft guidance represents the first FDA guidance initially composed by a disease community, with input from industry, sponsors, academia and the Duchenne muscular dystrophy patient community. When finalized, it will represent the Food and Drug Administration's (FDA's) current thinking on this topic. It does not create or confer any rights for or on any person and does not operate to bind FDA or the public. You can use an alternative approach if the approach satisfies the requirements of the applicable statutes and regulations. If you want to discuss an alternative approach, contact the FDA staff responsible for implementing this guidance. If you cannot identify the appropriate FDA staff, call the appropriate number listed on the title page of this guidance.

I. INTRODUCTION

The purpose of this guidance is to assist sponsors in the clinical development of medical products (i.e., human drugs, and therapeutic biological products) for the treatment of Duchenne muscular dystrophy (DMD) over the entire spectrum of the disease.

This guidance is the result of the first collaboration between the FDA and a disease specific community to produce clinical guidance in their respective disease area. The FDA invited the Duchenne community (including patients, parents and caregivers, clinicians, academic experts and industry) to develop the initial draft of this guidance as provided under FDA's interpretation of Good Guidance Practice provisions. The first iteration of the guidance, together with supporting papers from the working groups that produced it, can be found at [TBA]. Upon receipt of the guidance, the FDA opened a docket and held further meetings with the DMD community and other experts. The current document reflects some revisions based upon regulatory and statutory requirements and more recently published data.

This guidance addresses the FDA's current thinking regarding the consideration that should be given to the benefit/risk preferences of the DMD community considering the rarity and seriousness of the disease and in light of the lack of current causal treatment options. It also reflects the FDA's appreciation that the understanding of the natural history of DMD and causes for variability in outcomes has been updated

by recent evidence from patient registries, natural history studies and clinical trial cohorts. It addresses the selection of endpoints for clinical trials in populations with DMD as well as the manner in which disease modification might be demonstrated — and encourages the use and exploration of new biomarkers that could prove supportive of new drug applications, (consistent with FDA's stated interests in advancing personalized medicine efforts), particularly those with the potential to become surrogate endpoint markers for clinical development. Finally, the guidance discusses possible strategies that companies may pursue to secure one of the FDA's three pathways for expedited approval (meaning priority review, accelerated approval (based on subpart H provisions)) and prior designations of either breakthrough or fast track), as well as the evidence base required to secure traditional approval.

This guidance is intended to serve as a focus for continued discussions among the FDA, the medical industry, sponsors, academic community, the patient and caregiver community, and the public. The design of clinical trials that are specifically focused on the treatment of patients with Becker muscular dystrophy or any of the other muscular dystrophies is not explicitly discussed, although many of the principles in this guidance will be pertinent to the development of therapies for those conditions.

FDA's guidance documents, including this guidance, do not establish legally enforceable responsibilities. Instead, guidances describe the Agency's current thinking on a topic and should be viewed only as recommendations, unless specific regulatory or statutory requirements are cited. The use of the word *should* in Agency guidances means that something is suggested or recommended, but not required.

II. **BACKGROUND**

Duchenne muscular dystrophy is a genetic disorder characterized by the progressive degeneration and loss of skeletal muscle, the muscles of respiration and the cardiac muscle, primarily in boys — though a small percentage of female carriers may exhibit a range of muscle symptoms from the full Duchenne phenotype to milder skeletal muscle weakness. It is caused by mutations in the *DMD* gene that prevent the expression of functional dystrophin, an important structural component in muscle tissue. The condition is inherited in an X-linked manner and *de novo* mutations may occur in people from families without a known family history of the disease. DMD affects approximately 1 in 3,802 to 6,291 live male births worldwide.[1, 2]

In association with other proteins, dystrophin protects muscle fibers against the mechanical forces of contraction — in the absence of dystrophin, muscle is prone to damage, deterioration and fibrosis.

Muscle weakness generally becomes apparent in the first few years of life with a delay in motor milestones, and a mean age of walking around 18 months. Progressive muscle weakness leads to a loss of independent ambulation before the

age of thirteen in the absence of disease modifying treatment. In non-ambulatory boys and young men, there is gradual loss of upper limb and neck functions, so that grooming, toileting, bathing, dressing, and eating become impaired or impossible to do perform by oneself — affecting the quality of life of patients, their caregivers and families.

This is accompanied by weakness affecting respiratory muscles and the heart that contributes to decreased respiratory function and cardiomyopathy — with heart disease now being the most common cause of death in boys and young men with DMD.

Over the past decade, patient organizations, academia and industry have worked together to develop several patient registries, disseminate improved standards of care, and explore clinical outcome measures and biomarkers. This experience and data collection has resulted in a greatly improved understanding of the pathogenesis and the natural history of DMD, including factors that may lead to variability in the course of the disease.

Natural history studies have shown that the use of glucocorticoids and the management of spine deformity, pulmonary and cardiac dysfunctions have altered the timing of some of the clinical milestones of the disease. But with limited medical management have come new complications, and quality of life often suffers. For instance, adverse events known to be associated with glucocorticoid usage includes excessive weight gain, growth inhibition, risk of diabetes, behavioral abnormalities, Cushingoid features, change in pubertal progression and cataracts. Of particular concern for the Duchenne community is the issue of weight gain, since DMD is a progressively debilitating disease and weight gain can compound the physical limitations of a dystrophic myopathy.

At the time of writing, there are no FDA-approved DMD-specific therapies — and no way to reverse the underlying condition. Once ambulation or some other functional capacity is lost in an individual with Duchenne muscular dystrophy, it is gone forever. Death can happen without warning, at any moment, even in younger boys.

There is an urgent unmet need to develop new treatments, especially those that address the underlying cause of DMD. While a number of potential therapeutic agents are in or entering clinical development, sponsors need formal guidance on how best to demonstrate a treatment's effectiveness and safety in this rare disease and what sort of effect would be clinically meaningful to patients and their caregivers.

The FDA has acknowledged the concerns expressed by the DMD community that flexibility be exercised in the review of products for the disease — recognizing that many patients and care givers are willing to take greater risks for a treatment that may slow clinical deterioration or delay the loss of functional milestones, each of which is clinically meaningful.

When recommending this draft guidance, the Duchenne Community chose to place the topic of benefit/risk assessment at the start of the document, because it was felt that sponsors should be guided by patient and caregiver preferences from the very start of a product's clinical development program — and that sponsors also need clear guidance from the Agency on how benefit risk assessments contribute to a product's review process in the specific DMD disease context.

The FDA shares the Duchenne Community's goal to work with industry to get new therapeutic agents onto the market as rapidly and responsibly as possible. This guidance for industry is but a step towards achieving that goal.

III. BENEFIT/RISK ASSESSMENTS IN DUCHENNE MUSCULAR DYSTROPHY

A. General Comments

The assessment of benefits and risk plays a central role in the FDA review process. In recent years, the FDA has been mandated to make the assessment of benefits and risk more transparent and to better incorporate the perspective of patients and families into the benefit risk assessment. The FDA acknowledges that patients and families are already empowered to play a role in such decision. Specifically, the assessment of benefit and risk is fundamental to decisions made each day by patients and families, in collaboration with their healthcare professionals, about the use of treatments or devices.

To embrace a more patient/family centric approach to benefit-risk, sponsors are advised to quantify the preferences of patients and family members, when feasible. To promote patient-centered drug development, the FDA encourages industry to engage patients and family early in the development of therapy. Partnering with established advocacy efforts can be valuable in understanding the perspectives of patients and their family. Such partnerships can help the FDA better understand the preferences of patients and family members, and can guide the FDA in determining meaningful benefit, risk tolerance, acceptable tradeoffs and preference heterogeneity. The FDA accepts that the assessment of benefit and risk in DMD is complex. Although DMD is a debilitating disease that causes muscular deterioration and loss of function over the lifespan, the window of opportunity for a therapeutic intervention may come years before significant clinical events associated with morbidity and mortality — and yet, the consequences of failing to intervene are clear.

While regulators have traditionally been hesitant to allow risk in a young population, we are aware that parents of this population are willing to accept more uncertainty and take greater risk early on, because of the predictable outcomes in the disease.[3] For these reasons, activities with regards to the DMD patient and caregiver perspective should be done in consultation with a broader community rather than the traditional thinking of the community as subjects in a study.

We also appreciate that preferences of patients and caregivers may differ and that preferences may change over time. For progressive debilitating disorders, uncertainty about benefits and risks are weighed against the known implications of not treating. The certainty of disease progression is a compelling concern in the Duchenne benefit risk assessment.[4]

B. FDA's approach to benefit risk assessment

Benefit-risk analysis requires evidence on the benefits and risks of the therapy being investigated, an assessment of the certainty of the benefit and risk evidence, evaluation of risk mitigation strategies, and a subjective assessment of meaningful benefit, risk tolerance and an acceptable risk-benefit tradeoff. As the Agency has previously stated, risk-benefit assessments need to be tailored to the population being studied.

As described in the FDA framework, for each decision factor decision-makers consider both the evidence and its uncertainties, and use these to assess the implications of that component on the decision. The decision context combines analysis of the condition and of current treatment options. The analysis of the condition includes the natural course of the disease and its severity, and the assessment of current treatment options describes how well the medical need is met by currently available therapies.

Though this process is informed by data, the ultimate outcome relies on reviewer and Agency judgment as to what levels of risk are acceptable for particular levels of benefit. In recent years, there has been movement at the FDA to better understand patient preferences and to incorporate these in a transparent and scientific way in benefit-risk assessments. This concerns not only how the trial results are interpreted with regard to a regulatory decision, but how a clinical trial is conducted and in endpoint determinations. In addition, the information generated by a sponsor's clinical development program is also critical for the individual benefit/risk assessments made by the patient and caregivers.

These efforts to understand patient preferences and quantitative trade-offs are important in cases where there are uncertainties over benefit and risk. In other instances where benefits and risks are well characterized, it is frequently qualitatively understood how one might trade a risk for a benefit. Historically, the benefit/risk assessment has attempted to include patient/caregiver priorities predominately through the use of testimony. An important, outstanding question is how well the voice of those giving testimony reflects the perspectives of the broader disorder community.

While FDA encourages direct patient and community engagement with the agency regarding their risk benefit preferences at regulatory hearings and other forums, it is preferable to supplement this with a scientific approach—especially when benefits and risks are uncertain and not treating causes harm. In this way, input that has historically been ad hoc, unstructured narrative that is impossible to quantify or

generalize can be made quantifiable. Rigorous and generalizable approaches to quantifying risk benefit assessment hold weight in regulatory decisions about what constitutes meaningful benefit and acceptable risk, and sponsors should be aware that regulators can best integrate patient/caregiver preferences when those preferences are quantified using robust stated preferences methods. Consistent with FDA's mandate for more patient-centered regulatory decision-making, the Agency is interested in ways to take these preferences into account in evaluating options for accelerated development and approval of new treatments.

C. Incorporating patient/family preferences

A number of models and approaches have emerged in recent years to facilitate regulatory benefit-risk analysis.[5] These vary from simple graphical techniques[6, 7] to more comprehensive approaches that can be beneficial in the selection, organization, summary, and communication of evidence relevant to benefits and risks analysis.[8, 9] Methods to incorporate patient/family preferences have also emerged[10, 11, 12] and are now frequently found in the literature.[13, 14]

One of the most common approaches to measuring the perspectives of patients and their family are stated-preferences methods.[15] The most commonly applied of these stated-preference approaches is conjoint analysis – a broad class of methods that include discrete-choice experiments.[16, 17, 18] Such approaches aim at documenting acceptable tradeoffs across various risks and benefits. They can also be used to document the underlying heterogeneity of preferences[19] and to cluster individuals into groups with similar preferences.[20]

Duchenne Case Study

A collaborative advocacy/academic partnership demonstrated a community-engaged approach to measure caregiver preferences for potential benefits and risks of emerging therapies for DMD.[21] Caregivers' treatment preferences were measured using best-worst scaling. Attributes describing potential benefits and risks of emerging DMD therapies were identified through engagement with community stakeholders including advocates, clinicians, and drug developers. The attributes in this preliminary study included muscle function, lifespan, knowledge about the drug, nausea, risk of bleeds, and risk of arrhythmia. The study was implemented through an online survey of DMD caregivers, who were recruited in the US.

DMD caregivers identified moderate benefits of stabilizing or slowing progression of muscle weakness as the most important among experimental attributes (28.7%), followed by risk of heart arrhythmia (22.4%) and risk of bleeds (21.2%). Having additional post approval data (an attribute reflecting uncertainty) was relatively the least important attribute (2.3%).[22] As presented in the study, caregivers were willing to accept a serious risk when balanced with a non-curative treatment, even absent lifespan improvement. In other words, stabilization of the child's progression was considered a benefit worth a serious risk. However, caregivers indicated a limit to their risk tolerance in that they would not accept a risk of death and a risk of additional lifelong disability for a drug that stopped or slowed progression. This

study highlights the synergistic integration of traditional advocacy methods and scientific approach to quantify benefit-risk preferences.

D. Guidance to Sponsors

Clinical trial sponsors should take patient and/or caregiver preferences and priorities into account when designing clinical trials and when preparing for FDA submission. If relevant preference data does not already exist in the target decision-making population, sponsors should obtain this information. Patient and caregiver preferences may differ, and sponsors should explore the perceptions of the appropriate population(s) depending on the target and clinical trial protocol. Using a robust method to quantify preferences, clinical trial sponsors are encouraged to engage in patient-/community-centered research in preparing for FDA submission, both to inform the FDA benefit risk analysis and to engage a broader constituency in preparation for submissions. The patient-engaged approach should integrate topics (attributes) of importance to patients and families into the stated preferences experiments. Similar to other quantitative methods, stated preferences methods are only indicative of actual decision-making when the experimental attributes are relevant and understandable to individuals who are providing data.

Sponsors should explore risk mitigation strategies and patient/caregiver preferences for these strategies. Risk management and mitigation considers what activities are in place to optimize benefits while limiting the consequences of the risks, such as targeted monitoring, provider education strategies, controlled distribution, and special labeling. Particularly in conditions such as DMD — a pediatric-onset, lifelong disorder -- stakeholder concerns and preferences are relevant for informing the choice of risk-management strategies. Risk management approaches that engage treating clinicians may be particularly relevant for DMD, as a large proportion of the Duchenne population have health care providers that follow their patients for years.

Sponsors should anticipate that appraisals of benefit/risk will change over time due to disease and non-disease related factors, available treatment options, uncertainty, and other contextual influences. The type of benefit about which a patient or parent may be most concerned may vary according to their stage of disease: for instance, slowing the progressive loss of ambulation may be an important preference for the parents of a child who is still able to walk, while a non-ambulant patient may be much more concerned with upper arm function — and maintaining personal care ability. Such ongoing personal benefit-risk assessments make the patient-caregiver community a critical resource to engage in the development of benefit-risk research agenda.

E. Conclusions and special considerations for Duchenne

The FDA understands that communities facing progressive and fatal disorders, especially where there is a lack of effective treatment options, are willing to take greater risks than people with chronic and stable or slowly progressing disorders where there are existing treatments. For progressive disorders, uncertainty about

benefits or potential risk of any therapy must be weighed against the known implication of not treating. This certainty of disease progression without treatment should be included as a compelling harm in the benefit risk assessment. Consequently, the agency is more flexible in situations where there are no, or limited treatments, and more tolerant of uncertainty.

FDA benefit-risk assessment related to treatments for Duchenne will be strongly influenced by well-documented preferences of patients and families. The need to include patient/caregiver preferences is especially compelling for serious, progressive disorders with limited treatment options. Thus, sponsors should provide the FDA with robust data on patient/caregiver preferences. The FDA acknowledges the complexity of DMD and that meaningful benefit/risk tolerance and acceptable tradeoffs may vary across clinical subtypes, across the disease progression and/or as a consequence of preference heterogeneity across patients and caregivers. When possible, a properly designed and powered stated preferences study best provides insight into preference heterogeneity. The agency supports a model where data is collected in collaboration with patient/caregiver advocates or advocacy groups to empower patient communities and ensure meaningful preferences data.

Understanding evidence-based benefit risk preferences permits the Agency to exercise maximum flexibility in allowing accelerated access to therapies with appropriate labeling that clearly addresses uncertainty and describes benefits, side effects, harms to facilitate individual benefit risk choices. Sponsors should be aware that the FDA would consider labels that permit indications to provide access to early adopters of treatment — patients who are highly risk tolerant due to their desire to prevent or delay the loss of critical functional capacity and serious morbidity or mortality. Patients and caregivers, with support from their clinicians, can then make appropriate decisions for themselves with as much information on benefit and risk as available. This approach best meets the needs of the entire Duchenne community—patients, caregivers, clinicians, researchers, and industry—while achieving the FDA mandate to provide access faster for high-need rare disorder communities.

IV. **DIAGNOSTIC CRITERIA**

A. General Comments

The diagnosis of DMD is usually made by a neuromuscular specialist on the basis of family history, clinical features (delays in reaching developmental milestones and motor difficulties) followed by appropriate laboratory investigations.[23]

Guidance published by the American Academy of Pediatrics recommends that all children with motor delay and low muscle tone should initially be screened by measuring the serum creatine phosphokinase (CK) activity, which is significantly elevated in DMD, usually >1000 U/L.[24] This should be done irrespective of whether there is a family history of DMD, as about one third of the DMD cases result from spontaneous mutations in the *DMD* gene.[25] If serum CK activity is elevated, the

diagnosis of DMD should be confirmed with molecular analysis of the *DMD* gene or by assessment of dystrophin protein expression on muscle biopsy.

The DMD diagnostic odyssey

Sponsors should be aware that despite early signs of weakness, there may be significant delays in arriving at a DMD diagnosis because parents do not voice their concerns or local healthcare professionals are not familiar with the disease, resulting in a delay in pursuing testing. The delay can be substantial — one cohort, MD-STARnet—reported a delay of 2.5 years from the time symptoms were first noticed.[26] Another cohort reported that the mean age at diagnosis was 4 years and 10 months (SE 3·9 months, range 16–99 months).[27] Less than a third of the boys in that cohort were diagnosed before the age of 4 years (32%) — many were in school, and their developmental delays had been noted by teachers, but not health professionals. This suggests education of practitioners is key to shortening the diagnostic odyssey (see the American Academy of Pediatrics statement on the childmuscleweakness.org).

There may also be financial barriers to completing a referral to a specialist. In addition, there is lack of uniformity in access or availability of genetic testing that may also contribute somewhat to a delayed diagnosis as well.

Sponsors should be aware, however, that there are a number of initiatives underway to increase awareness among primary clinicians of what steps to take in children with developmental delays.

B. Diagnostic Laboratory Investigations/Methods for confirming diagnosis

Genetic analysis

In young boys with the clinical features suggestive of DMD and elevated CK, analysis of the *DMD* gene from genomic DNA (such as lymphocyte-derived DNA) is diagnostic in ~95 % of cases. Approximately 60% of mutations are large-scale deletions, 5% are duplications, and the remainder, detectable from genomic DNA, are point mutations or small deletions/insertions. The remaining ~5% of mutations are due to intronic mutations that are undetectable by standard genomic analysis but result in altered splicing only detectable by mRNA analysis from muscle tissue.[28, 29] Thus, lack of a detectable DMD gene mutation using standard methodology does not exclude a DMD diagnosis. As discussed in further detail below, muscle biopsy and dystrophin expression analysis remains the gold standard of diagnosis, remaining particularly useful in cases where no mutation was detected by standard clinical molecular diagnostic testing.

The understanding of relationship between the location, type or the size of the mutations in the *DMD* gene and the severity of phenotype is evolving.[30] However, genetic testing in the *DMD* gene can be contrasted to other genotypic tests due to the high probability of finding a mutation and the capacity to predict with a degree of certainty what that functional effect of that mutation will be for dystrophin

production for most cases. DMD overwhelmingly results from mutations predicted to lead to truncated dystrophin protein, with missense mutations in specific functional domains found only rarely. Therefore the common genetic test practice of reporting of 'variants of uncertain significance' is rarely used for a patient with Duchenne muscular dystrophy. If a muscle biopsy shows absence of dystrophin, a genetic analysis is considered the standard of care even if the diagnosis made on the basis of protein.

Methodology

The quality and type of diagnostic testing may need to be considered in the context of the sponsor's specific study. There are now a number of established genetic testing techniques, and methods can be expected to continue to evolve. However, for changes in exon copy number (deletions and duplications), any acceptable modern molecular diagnostic method must interrogate all exons to establish completely the extent of the deletion/duplication. Similarly, methods of sequence analysis should provide sequence coverage of the entire coding region in probands. Patients who have been screened with older technologies may need to be re-tested in order to more accurately characterize their mutations

Testing should be performed at a CLIA (and, potentially, CAP) certified lab (or their equivalent outside of the US) and the results interpreted by a qualified professional.

Access to genetic testing

Barriers that limit access to genetic testing exist and these include financial and health care provider education. Partnerships between advocacy and industry can facilitate access to genetic testing [for example: Decode Duchenne program (https://www.duchenneconnect.org/)].

Dystrophin expression on muscle biopsy as a diagnostic biomarker

Immunohistochemical, immunofluorescent, or Western blot analysis can show the relative amount of dystrophin in skeletal muscle specimen, and Western blot can reveal its size, helping to distinguish between DMD and milder muscular dystrophy phenotype such as Becker muscular dystrophy (BMD). An amount of dystrophin of less than 3% of normal has been described as consistent with DMD, and greater than 20% as consistent with mild BMD,[31] but as discussed in the Biomarkers section, standardization of dystrophin quantification is challenging, and in current clinical diagnostic practice, dystrophin expression is frequently descriptive or semi-quantitative. BMD is typically caused by in frame DMD mutations resulting in the expression of internally truncated dystrophin proteins. The expression levels and functionality of these different dystrophin proteins vary and contribute to the variable phenotype in BMD yet without a linear correlation between dystrophin levels and phenotype.[32,33,34,35] Correlation of dystrophin amounts with clinical benefit has been seen in female DMD carriers (normal dystrophin).[36, 37, 38] It is expected that the laboratory will make every effort to maintain good laboratory practice as outlined in the Biomarkers chapter (page 36), section, "The handling of the biopsy."

C. Newborn screening

Newborn screening has been performed via measurement of CK activity on dried blood spots from neonates, and the ability to use the same blood spot for *DMD* mutational analysis has recently been shown to be feasible.[39]

Because of the expected importance of early therapeutic intervention, there is enthusiasm in the community for newborn screening. At the national and international level, it remains under administrative review and there are potential problems to be resolved (ex. non-DMD patients identified, informed consent, cost).

This is something that remains in evolution at the time of drafting this document.

D. The spectrum and clinical classification of dystrophinopathies

The amount and size of dystrophin in the muscle biopsy tends to predict the severity of muscular dystrophy, while genomic analysis also has a high degree of predicted value for disease phenotype. But sponsors should be aware that a molecular diagnosis is not the same as the clinical diagnosis, and does not with a hundred percent certainty determine phenotype.[40] Although genomic analysis generally has a high predictive value depending on the mutation, and prognosis based on the open reading frame is usually quite effective at differentiating DMD from BMD, detailed analyses of larger series of BMD patients have documented many apparently out-of-frame mutations where dystrophin production was still seen by various molecular mechanisms. For example, some predicted nonsense mutations are associated with Becker muscular dystrophy, a clinically milder disorder.[41]

Thus genotype alone does not determine classification in all patients, and although genetic and protein results can make predictions, they do not replace the clinical assessment in determining where a patient is on the spectrum of dystrophin-related muscular dystrophy. Recognizing this spectrum of dystrophinopathies — and heterogeneity in the course of progression — some experts have proposed further sub-categorization of dystrophinopathies to include, for example, an intermediate (IMD) form falling between classic DMD and more severe BMD based upon clinical grounds. While the Agency does not believe these sub-categorizations warrant distinctions in access to new therapies, they may represent a source of heterogeneity that sponsors should take into consideration when conducting clinical trials.

V. THE CURRENT UNDERSTANDING OF THE NATURAL HISTORY OF DUCHENNE MUSCULAR DYSTROPHY

A. General comments

The natural history of DMD is much better characterized today than it was ten to twenty years ago, as a consequence of patient registries, natural history studies and data drawn from the placebo arms of industry trials (which have been to shown to

correspond closely to the natural history data).[42, 43] Over the same period, improved medical management has been shown to prolong survival and slightly slow disease progression. Despite this, the unmet medical need and urgency for improved therapies for DMD is profound. Progressive quadriparesis during the first two decades due to dystrophin deficiency and skeletal muscle fiber loss remains the common disease course. Pulmonary insufficiency from skeletal muscle involvement and cardiomyopathy leads to substantially shortened lifespans among patients receiving even optimal care. Sponsors should be aware of how the current understanding of the natural history of DMD provides opportunities for the evaluation of new treatments across the spectrum of the disease.

B. Testing and evaluation across the spectrum of disease

Sponsors should be aware of the variety of testing and evaluation tools that have been used to measure developmental delay, functional loss and other parameters of progression of DMD. The use of these outcome measures in natural history studies and patient registries have helped to better characterize the natural history of the disease.

Beginning in 2010, the International DMD Clinical Outcomes Working Group met on a number of occasions in order to reach international consensus on age-appropriate clinical outcome measures for use in the study of subjects with DMD.[44] In addition to mapping out the outcome measures currently being used, they reviewed data from eight large contemporary cohorts (including more than 1900 subjects across the spectrum of the disease followed over a twenty year period) in order to establish the current expected clinical course of the disease and determine whether data existed to define the relationship between the outcome measures and milestones of disease progression. Where deficiencies and endpoints occurred across the spectrum of disease, working subgroups were formed to work collaboratively to validate new endpoints.[45]

The following schematic, adapted from the proceedings of those meetings, includes a number of outcome measures that are most widely used, and where there is broad scientific consensus regarding their utility in DMD, organized by the age group and disease stage in which they are used. In order for an assessment tool or outcome measure to be included in this schematic, there needed to be consensus that there was a conceptual framework for the endpoints that fit Duchenne dystrophy and data on reliability, concurrent validity, cross validation (with other endpoints), normative ranges, experience with the endpoints and existing longitudinal data in ongoing natural history studies, evidence of responsiveness to a treatment, and clinical meaningfulness. Finally, the tool had to have been successfully implemented in multinational clinical trials.[46] Guidance pertaining to inclusion of specific outcome measures in clinical intervention trials is discussed in Section F (DMD Clinical Trial Designs, Endpoints, and Considerations).

Figure 1

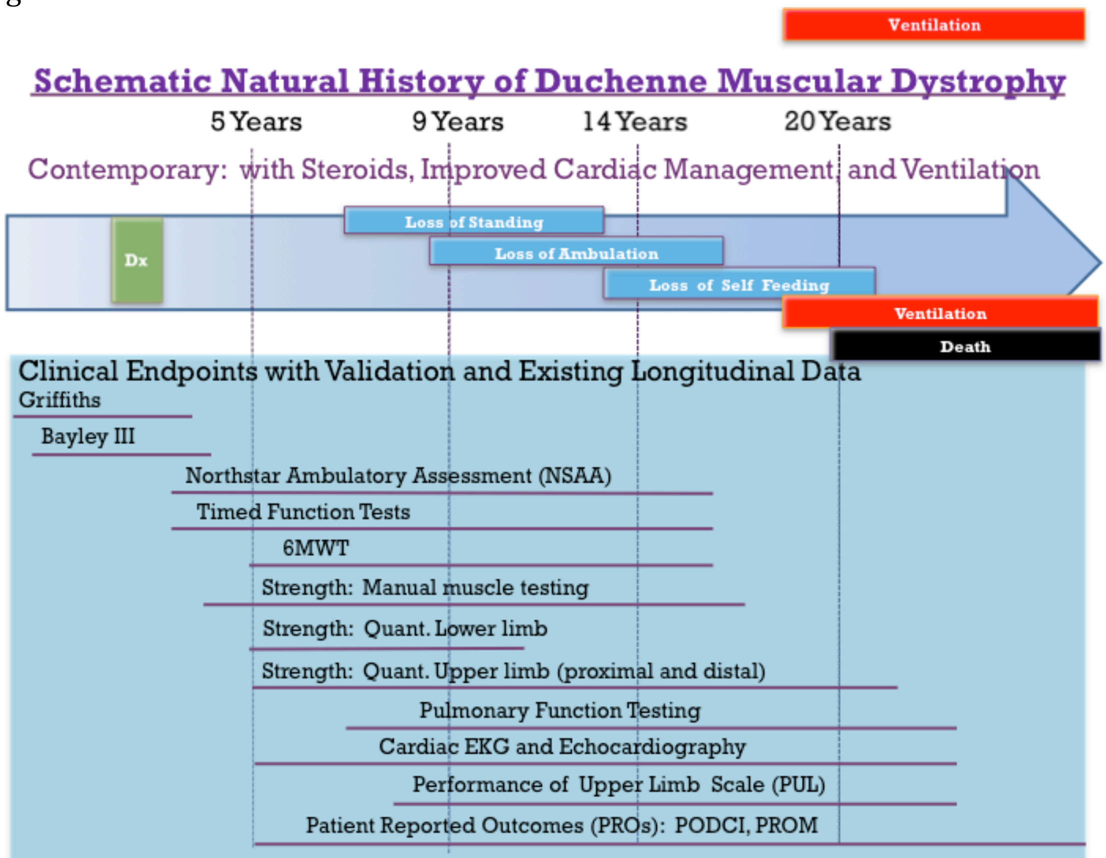

47, 48

Cardiac MRI is an important emerging measure that has yet to meet all of the criteria to be included in the schematic (similarly, skeletal muscle MRI is a promising measure, described in more detail later in this document).

C. Overview of the Natural History in Duchenne muscular dystrophy

What follows is a brief overview of the current natural history of DMD across the spectrum of disease. Furthermore, while specific functional changes are observed at different ages, it should be emphasized that the disease is due to generalized skeletal muscle involvement and cardiomyopathy and pathological processes involved in DMD are ongoing over the course of a patient's lifetime — while loss of ambulatory capacity and gross motor functions may be a primary focus in ambulatory boys, neuromuscular deterioration may already be measurable in the upper limb and other muscle groups. Note, the ages are approximations, and the intent is not to create artificial stages of disease.

- *Neonates/Infancy:* While DMD is rarely diagnosed in infancy, the disease is manifested at birth. Even though some of the infants detected due to family history are sometimes referred to as being asymptomatic, most will still show delayed development if evaluated with tools such as the Griffiths Mental Development Scales, an outcome measure than can be used in the very young (6-

47 months)[49] and the Bayley Scales of Infant and Toddler Development, Third Edition (Bayley-III).[50],[51] One study of children with DMD with mutations upstream or in exon 44 had higher Developmental Quotient (DQ) than those with mutations downstream exon 44 which are associated with involvement of dystrophin isoforms expressed at high levels in brain. The difference was significant for total and individual subscale DQ with the exception of the locomotor subscale. Items, such as ability to run fast, or getting up from the floor consistently failed in all children, irrespective of the age or of the site of mutation.[52]

- *Young children, early ambulant (aged one to 42 months):* The development of gross motor milestones is typically slower than in boys without Duchenne, and some children may show signs of delayed language and cognitive impairment. Toddlers and young children may also be scored with developmental outcome measures such as the Bayley-III and Griffith's Developmental Scales. [53], [54] Gross motor scores were lower in young children with DMD at baseline compared with published controls and revealed a further declining trend at 6 months. Repeated measures analysis over 12 months revealed that gross motor scores declined further at 12 months. Cognitive and language scores were lower at baseline compared with typically developing children and did not change significantly at 6 or 12 months. Fine motor skills, also low at baseline, improved over 1 year.
- *Young Ambulatory (from four to approximately seven):* A period where there may be slower gains in ambulatory function as compared to typically developing children (on 6MWT and 10 meter walk/run tests) and either gains or losses in milestones as noted by endpoints such as the North Star Ambulatory Assessment (NSAA). However, it is important to note that physiologic deterioration is ongoing and boys are increasingly falling behind normative performance levels of their normally functioning peer group.
- *Late Ambulatory (from approximately seven to thirteen years of age)*: *G*enerally defined as when individuals begin to suffer a decline in their gross motor functions as well as some pulmonary function parameters, particularly maximal expiratory pressure (MEP) and maximal inspiratory pressure (MIP). During this stage of disease, there is marked progressive loss of muscle fiber in the proximal muscles, growing weakness and the gradual loss of gross motor skills and ambulatory functions (including standing ability, stair climbing and ultimately, the ability to walk). Ankle equinus contractures are the most common skeletal deformity. There is risk of osteopenia and fractures. There is also a comparative loss in height and increased weight gain in comparison their normally functioning peer group.
- *Early Non-ambulatory (beginning the age when a boy starts using a wheelchair full-time):* After boys can no longer walk, there is continued muscular deterioration throughout the upper and lower limbs, and skeletal deformities such as limb contractures and spine deformity may become problematic. Powered mobility is required after loss of ambulation. Postural maintenance and sitting balance is initially intact and progressively lost. There is increasing loss of upper limb function (with decreasing ability to reach overhead, dress, self-feed,

and perform other self-care). There is continued decline in pulmonary function with ultimate need for mechanical cough assistance and progressive risk of nocturnal hypoventilation requiring non-invasive ventilation. Cardiomyopathy is evident by cardiac MRI in virtually all patients and in some patients by cardiac echo. After transition to a wheelchair, patients tend to put on more weight compared to their normally functioning peer group.

- *Late non-ambulatory:* Postural support of the trunk and head support from a seating system is required as well as power recline. Upper extremity function is severely limited to distal fine motor function and tabletop activities. Maintaining computer access is a critical quality of life concern. Virtually all patients benefit from mechanical cough assistance and there is a high risk of nocturnal and daytime hypoventilation requiring non-invasive ventilation. Optimal nutritional management may require gastrostomy tube placement and enteral formula supplementation. There is risk for dysphagia and aspiration. Adequate phonation may become an issue late in the disease course. There may be a larger number of older DMD patients with unmet medical needs. As patients age, respiratory impairment and heart disease (heart failure and conduction abnormalities) are causes of morbidity and, eventually, mortality.

Both progressive limb weakness and decline in pulmonary function are due to skeletal myopathy, however, cardiac deterioration due to progressive cardiomyopathy may not be correlated with skeletal muscle deterioration. With increased lifespan due to effective ventilation interventions, cardiomyopathy has become a more common cause of death among patients with DMD.[55] Cardiomyopathy in DMD does not usually manifest clinically until later phases of disease progression, but is likely present to some degree beginning at birth. However, the concept that cardiac disease develops only later along the spectrum of DMD progression does not appear to be the case — imaging data suggest a proportion of boys already show fibrosis in the heart at ages as young as six.

The loss of clinical milestones is a hallmark of disease progression in DMD. Prior to and after the loss of ambulation, the difficulty performing functions and the loss of milestones, occur in a generally predictable descending order (although there may be some overlap or slight variation in some of the milestones).

Ambulatory functions and milestones

- Unable to jump, hop, and run
- Gower's sign with standing
- Loss of standing from the floor
- Loss of transition from lying supine to sit
- Loss of stair climbing
- Loss of ability to stand from a chair
- Loss of ability to walk independently (defined by inability to perform 10 meter walk/run)
- Loss of standing in place

Non-ambulatory milestones (descending order)

- Loss of ability to reach overhead
- Loss of ability to reach the scalp
- Loss of ability to self-feed without adaptations (hand to mouth)
- Loss of ability to place hands to table top
- Loss of ability to use a computer (distal hand function)

Pulmonary milestones are primarily measured by forced vital capacity, peak cough flow, and maximal static airway pressures (MIP and MEP)) indicating a need for interventions as outlined in the DMD Care Considerations.[56]

- $\leq$50% predicted FVC (cough assistance; monitoring required)
- $\leq$ 40% predicted FVC (non-invasive ventilation should be a consideration)
- $\leq$ 30% FVC (inability to sustain adequate overnight ventilation without support is likely)
- Maximum expiratory pressure (MEP) < 60 cm water (preoperative training in and postoperative use of manual and assisted cough techniques are necessary)
- Peak cough flow <160 L/min (manual and mechanically assisted cough techniques necessary)
- MEP <40 cm water (manual and mechanically assisted cough techniques necessary on a daily basis)

Cardiac milestones

- Normal Ejection fraction (afterload reduction with angiotensin converting enzyme (ACE) inhibitors, or Angiotensin II receptor blockers (ARBs), recommended by some cardiologists)
- < 55% Ejection fraction (most would agree that cardiac medications indicated)

D. How optimal medical management affects the course of DMD

Sponsors should be aware that current medical management (as depicted in Figure 1) has changed the natural history in DMD affecting the timing of clinically meaningful milestones in individuals with access to high quality care. This has largely been due to the use of glucocorticoids, management of spine deformity, pulmonary management, and cardiac management. The occurrence of contractures may impact mobility and upper limb function and efforts are made to prevent and manage contractures. Despite these interventions, the cardiomyopathy and pulmonary involvement in DMD still leads to substantially shortened lifespan. In the CINRG natural history cohort of 340 subjects followed prospectively from 2006-2011, death occurred in 5% of the cohort (5%) and age at death ranged from 9.9 years to 29.5 years.[57]

Glucocorticoid therapy: Corticosteroids have had an effect on all-cause survival as well[58] (although, their effects on DMD-related heart disease are, however, somewhat

more equivocal). A Cochrane review has also concluded that glucocorticoid corticosteroids improve muscle strength and function over six months to two years. Improvements were seen in time taken to rise from the floor (Gower's maneuver time), nine meters walking time, four-stair climbing time, the ability to lift weights, leg function grade and forced vital capacity.[59] In several natural history studies, steroids have been shown to delay the loss of ambulatory milestones, prolonging ambulation by about two to three years over time and delayed losses in upper-limb functioning — so that young men can continue to raise their hand to their mouths and feed themselves for a longer period of time.[60] Steroids have also affected pulmonary function — young men treated with steroids reach an older age before requiring mechanical cough assistance or non-invasive ventilation as defined by FVC parameters outlined in the DMD care considerations.[61] However, as previously noted, glucocorticoid therapy also comes with substantial adverse events.

Contracture management: While ankle equinus contractures begin in the late ambulatory stage and may contribute to the loss of stair climbing and ambulatory capacity, most lower limb and upper limb contractures occur subsequent to the loss of ambulation.[62] Approaches to contracture prevention and management have been outlined in the care considerations,[63] but the efficacy of these approaches has not been established. To the extent that contractures are directly related to antigravity strength (movement against gravity through a full range of motion), and ambulatory capacity, any intervention that maintains strength, function, and upright mobility will likely result in decreased contractures.[64]

Spine deformity management: The incidence of significant scoliosis requiring spinal arthrodesis has changed due to the use of glucocorticoids.[65] In addition, timely spine surgery for curves > 30-40 degrees has impacted survival.[66]

Pulmonary management: The American Thoracic Society practice parameter[67] includes recommendations for management of DMD with airway clearance strategies or mechanical cough assistance and non-invasive ventilation. Survival has been most impacted by ventilation — two recent studies have reported that lifespans in Duchenne can be lengthened substantially due to the implementation of non-invasive ventilation.[68, 69] Consequently, a larger number of young men with DMD are living into their twenties and thirties but often with significant disability. In addition, early death is still commonly observed in individuals with DMD in the early teen years to early 20's, mostly due to heart problems.

Cardiac management: The cardiac management has evolved from treatment of symptomatic heart failure to prevention of progressive ventricular dysfunction with early afterload reduction e.g., angiotensin converting enzyme (ACE) inhibitors, Angiotensin II receptor blockers (ARBs), and beta blockers). ACE inhibitors have impacted positively on survival in young men with DMD-associated clinical cardiomyopathy, by reducing stress on the heart (afterload reduction).[70] Data are needed on the combined effect of afterload reduction and corticosteroids on the development of ventricular dysfunction. Cardiac conduction abnormalities are

screened for with ECG and Holter monitoring. The use of left ventricular assist devices and cardiac transplantation in DMD is an evolving topic.[71]

The standard of care received by patients with Duchenne therefore has significant implications on the design of trials in the population, depending upon the outcomes being measured.

E. Heterogeneity in DMD disease progression: Predictability and sources of variability

The goal of therapeutics in DMD is to slow or stabilize disease progression in comparison to that expected by natural history. It should be noted that there is heterogeneity observed among patients in terms of disease progression, as noted in the diagnostics chapter, in that some patients with DMD may experience more aggressive rates of progression than others. The clinical heterogeneity of Duchenne muscular dystrophy (DMD) was once viewed as a major obstacle to the interpretation of therapeutic trials but with an evolution in the understanding of natural history, this may no longer be the case.

Sources of variability

With more data coming from natural history studies and the placebo arms from Duchenne treatment studies, many of the causes for variability in outcomes are becoming clearer. Sponsors should consider taking the following causes of heterogeneity into account when designing their phase II and phase III studies. The following critical elements are at least identifiable and could possibly have a large enough effect size that trial designs should manage them.

- Disease severity / stage of disease

Some variability in future progression is explained on the basis of disease severity, stage of the disease, and known natural history. For example baseline levels of function predict subsequent disease progression in DMD. Higher baseline function or stabilization of baseline function over the short-term is almost always associated with slower long-term decline.[72, 73] Lower baseline function may be associated with rapid subsequent decline in ambulatory endpoints when patients have passed critical thresholds of strength and function. Baseline measures of ambulatory capacity have been used to stratify cohorts in DMD trials.

The age at loss of clinically meaningful milestones (a proxy for disease severity) also predicts the age at loss of future milestones. For example, the age at loss of ambulation predicts the age at which subsequent loss of upper limb functions occurs and the age at which critical pulmonary milestones are reached.[74] It follows that changes in some clinical outcome measures in response to treatment over the short term, can predict subsequent disease progression years later. This has been demonstrated in children using corticosteroid treatment followed for many years[75].

Sponsors should take care to prevent an imbalance in the ages of study participants, which can introduce substantial variability into a trial. It is critical that control and

treatment arms in clinical trials be appropriately matched by age and functional status. Baseline functional performance — in relation to specific endpoints such as 6MWT, or time function tests — should also be taken into account as it does have an impact on the subsequent rate of progression over time.

- Genetic predictors of disease progression

Mutations within the *DMD* gene (exon-skippable mutations/deletions or nonsense mutations, deletions, duplications, point mutations) may be associated with an altered course of progression from one another. One study suggests that there is a trend for children with duplication mutations to perform better than the cohort as a whole.[76] Within those with deletions, there are specific subgroups that appear to be different from each other. For instance, there is a trend towards better baselines and less severe decline in progression as measured by 6MWT in boys eligible for skipping of exon-44 when compared to those with boys eligible for skipping at exons 45 and 53.[77] The exact effect size is being determined as this document is being written. With larger cohorts or longer follow-up, differences between subgroups may become significant.

However, while there may be differences between subgroups of patients with specific mutations, the mean 12-month changes in each subgroup falls within a narrow range in comparison to the mean of the whole DMD cohort. Furthermore, it should be recognized that some variability will be present within specific subgroups due to the many sources of heterogeneity listed here.

It is also worth noting that some mutations appear to select for more dystrophin-related abnormalities in non-skeletal muscle causing more pulmonary, cardiac and neurocognitive impairment.[78, 79]

Clinical trials of treatments that are not mutation specific should collect appropriate samples for full genetic analysis. As noted in the diagnostics chapter, some trial participants may need to be rescreened with a technique that provides a complete analysis of the *DMD* gene (see diagnostics chapter).

- Genetic modifiers

Genetic screening has identified polymorphisms in other genes that may have altered aspects of the response of muscle to dystrophin deficiency and/or drug treatment (e.g. glucocorticoids). These genetic modifiers may modify the onset, severity, or drug responsiveness of Duchenne muscular dystrophy patients, and are instructive regarding key biochemical pathways involved in muscle damage, repair or response to steroids. They are also important in increasing understanding of factors responsible for patient-patient variability, and could eventually prove helpful in interpreting clinical trial data. Genetic modifier studies, as with most genetic association studies in any human trait, typically require large numbers of patients studied using reliable and sensitive biochemical or clinical outcome measures. Differences in methods of characterizing or categorizing cohorts of

patients, as well as ethnic differences in polymorphism allele frequencies can lead to challenges in statistical analyses and reproducibility of genetic association studies. To date, two potential genetic modifiers have been identified:

Latent TGF-beta-binding protein 4 (LTBP4) polymorphisms: A minor allele present in about thirty percent of the population appears to have a protective effect on ambulation roughly equivalent to the effect of steroid treatment, prolonging ambulation by as much as two years.[80]

Secreted phosphoprotein 1 (SPP1 or osteopontin) polymorphisms: In the case of osteopontin, the genetic modifier may actually be modifying patient's responses to corticosteroid management rather than affecting the disease itself directly.[81]

There may be other genetic modifiers yet to be identified. However, at the time of writing, data on genetic modifiers comes from small cohorts and the effect sizes are not yet clearly defined. Sponsors should review the most current data on the subject to see whether screening for these genetic modifiers in their clinical trials is advisable, for stratification or planned post-hoc analyses to explain potential causes of variation in the outcomes of patients.

- Corticosteroid therapy

There are data to suggest that differences in patterns of steroid use — including whether the patient is on daily versus intermittent regimens, dosage, time on treatment, and possible drug choice (deflazacort or prednisone) — may have variable effects on clinical progression and function.[82, 83] (Note, at the time of writing, deflazacort is not yet marketed in the US, though some individuals have acquired access to it). Since medical management of DMD with corticosteroids tends to be individualized, differences in side effects between the steroid regimes may also result in differences in how clinicians adjust the dose or in patient/caregiver adherence. .

Enrollment in the trials should either be restricted or stratified according to harmonized corticosteroid therapy. Historically, six months of stable corticosteroid therapy has been used as inclusion criteria however, sponsors of clinical trials should be aware that some ambulatory boys may continue to have functional improvements beyond six months on corticosteroid treatment.

- Night splinting, physical therapy, and other standard interventions

It is important to note that night splinting, physical therapy and other standard of care interventions as described by the DMD Care Considerations are recommended because they are expected to have significant effects on functional performance.[84] Significant variability in the course of progression could be introduced depending upon whether or not a person with DMD receives standard of care contracture prevention and management, or is adherent to recommended prevention and management strategies.

Sponsors of clinical trials should make certain that the standards of care are observed at every center that is involved in their studies — including both pulmonary support, night splints and stretching which could make a difference in children's performance on functional measures. Sponsors should also take note of concurrent complementary therapy study participants may be taking. Some studies are attempting to monitor the family's adherence to physiotherapy, home stretching and splinting, in an effort to capture these variables for possible post-hoc analyses.

Ideally, sponsors should control for as many of these factors as possible to reduce potential variability in disease course among participants in their clinical trials.

F. Ongoing natural history study needs

Given the relentless course of DMD and the difficulty in conducting adequately powered studies in a rare disease, there is need to establish adequate, reliable and well-matched natural history controls that account for known causes in variability of the disease. To be useful for natural history controls, the collection of natural history data must be of a certain rigor to satisfy FDA requirements.

Sponsors should refer to the following three documents for guidance:

- FDA Guidance for Industry - Computerized Systems Used in Clinical Investigations (2007)
- CFR Part 11, Subpart B – Electronic Records
- ICH Guidelines for Good Clinical Practice – Section 4.9 (Records and Reports), and Section 5.5 (Trial Management, Data Handling, and Record Keeping

VI. DMD CLINICAL TRIAL DESIGNS, OUTCOME MEASURES AND CONSIDERATIONS

A. General Comments

The purpose of clinical research is to better understand the disease process, natural history, patient experience and current treatment options. In contrast, regulatory guidance for trial designs and outcome measures is intended to serve as an evaluation tool for consistency of assessment of efficacy of new therapeutics in the context of both first and subsequent generation of therapeutics.

Recognizing the need to bring drugs to market efficiently, sponsors are invited to discuss with the FDA how their drug development package can best gain experience and document safety in the different DMD populations, including what studies or programs might be put in place prior to marketing, as well as post-marketing commitments.

B. Maximizing inclusion of populations in studies

DMD is a rare disease. In the US, there are approximately only 500 boys born with DMD each year — and the pool of subjects available for each personalized therapy is

smaller still. Very young boys have historically been the under-diagnosed, while the numbers of older non-ambulatory boys and young men who can participate in clinical trials is limited by mobility, burden of participation, lack of experience with endpoints, and death.

Nevertheless, the DMD community has advocated that potential therapies targeting DMD be evaluated across the spectrum of disease. Sponsors are encouraged to study new drugs in different age groups and disease stages in order to gain a better understanding of a potential therapy's safety and how it might work across the entire spectrum of the disease.

To date, most studies have been performed on ambulatory boys — largely because of the selection of a change in 6MWD as the primary endpoint in clinical trials. Yet there is a strong physiological rationale for the benefit of earlier treatment, as therapies that preserve muscle, in particular, are likely to have the greatest impact on prognosis before muscle health has deteriorated.

Until there is an increase in newborn screening, early detection of very young patients may be complicated; however, this is the population that, in principle, would achieve the greatest benefit from an intervention because they have the least accumulated injury. There are special ethical circumstances that need to be considered in an individual and compassionate way when screening in families at risk — and approximately one third of boys with DMD will represent a new mutation in the dystrophin gene.[85]

Nevertheless, it is not only possible but also feasible for a sponsor to identify some young patients including neonates in special circumstances. Once there is adequate safety data to move into very young children, we anticipate that sponsors will be compliant with current pediatric regulation to develop a therapy and test it in this population.

Employing overly restrictive entry criteria in a rare disease poses a danger to successful recruitment. Patients with Duchenne muscular dystrophy with a wide range of cognitive impairment have been able to participate in clinical trials as long as they can perform the outcome measures.

There is also a need to understand safety and efficacy at later stages of disease — and to see whether treatments that protect skeletal muscle also preserve heart and respiratory function. Demonstrating safety in a broader population would also lend support to a wide labeling for the product.

At present, there is no single instrument that can measure clinical outcomes and is equally sensitive to change across the entire spectrum of DMD over the course of a six to eighteen month study. Sponsors are encouraged to consider the use or validation of instruments that could expand the population (age group/disease status) that can be studied in one setting.

C. Clinical outcome measures and endpoints in DMD

Although to date, most trials in DMD have focused on ambulatory patients, there is broad international consensus on a range of age-appropriate clinical outcome measures that could be considered in the study of subjects with DMD. Clinically meaningful loss of capacity can occur in a number of functional domains that merit consideration as clinical endpoints in a trial. The appropriateness of outcome measures depends on age and functional capacity of study participants, and the mechanism of action of the drug. Sponsors may consider monitoring pulmonary and cardiac status even in younger patients, with outcome measures that are sensitive in younger populations (see discussion below). This may be useful to demonstrate long-term benefit and to anticipate newer, more sensitive measures of cardiac change or pulmonary specific change.

Treatment effects may vary by muscle group depending upon 1) the stage of disease, 2) the differential rate of progression of each muscle group in that stage, 3) the muscle fiber type, 4) the drug's mechanism of action, 5) the bio-distribution of the drug to different tissues and muscle fiber types, 6) the route of administration, and the medical addressability of the disease itself — there may be a point where a muscle has deteriorated beyond a point at which it can respond to therapy.

Motor Outcome Measures:

Motor outcomes measures exist across the age spectrum of DMD. However, sponsors should consider whether the following age/disease specific outcome measures, which have been used to characterize the natural history of DMD could be adapted for use as clinical endpoints in their development program.

Not all motor endpoints are measuring the same phenomenon. For example, there might be correlation at baseline of, for example, the 6MWT and the TFTs, because 6MWT is an endurance test, and muscle perfusion and metabolism are important in prolonged exercise. However, drugs that might improve muscle perfusion or metabolism could impact the 6MWT while not impacting, in a short term, the TFTs or muscle strength in the short term. Consequently, the selection of a motor endpoint for a specific drug program needs to be based on the mechanism of action of the drug as well as the age appropriateness.

The sponsor will need to ensure that these or similar outcome measures are appropriately validated in the population in which they are studied.

For example, **in neonates, infants, and young children up to age 4**, developmental scales have been used in DMD, i.e. the Griffiths Scale of Mental Development or the Bayley III Scales of Infant and Toddler Development BSID-III[86, 87, 88] Many development scales require formal training and certification on the part of the clinical evaluator. The sponsor should consider the availability of language and country specific validation of each scale in choosing an outcome measure, as well as understand the limitations posed by the end of range effects of each scale.

Developmental scales may also undergo revision over time and may pose additional challenges in interpretation.

For young Ambulatory (from four to approximately seven):

- **The North Star Ambulatory Assessment** is a useful measure of gross motor function in ambulant children from the age of four into adolescence. It was developed with a Duchenne disease progression construct in mind and is reliable, validated against other endpoints and is clinically meaningful.[89, 90, 91, 92, 93, 94] The measure has been shown to be sufficiently responsive to differentiate disease progression in children with DMD on continuous versus intermittent steroids.[95] Recent clinical trials using dystrophin restoration strategies have not shown the NSAA to be sufficiently sensitive to changes in disease progression over 48 weeks but it may be considered for longer duration trials.
- **Time to stand from supine has** been used for decades as a clinical trial endpoint in DMD.[96, 97, 98] Loss of standing ability has been shown to be predictive of time to loss of ambulation and time to 10 % decline in ambulatory function. (McDonald 2013b) It is reliably obtained in younger DMD subjects[99] and a useful endpoint for younger DMD patients. Limitations include the early loss of the endpoint in many boys with DMD, and reduced sensitivity of the endpoint as defined by the ratio of the minimally clinically important difference (MCID), which is greater than 3 seconds in DMD, to the mean baseline value.[100]
- **Time to run / walk 10 meters** is another timed function test used for decades as a clinical trial endpoint in children with DMD ages 4 and older.[101, 102, 103] It is easily obtained in the clinic and reliable in younger children.[104] The velocity of the 10 meter run / walk increases in DMD up to age seven but not to the same extent as seen in typically developing children. It is reliably assessed and validated with other endpoints. It is predictive of future loss of ambulation. A change of on the order of several seconds or less has been shown to be clinically meaningful.[105, 106]
- **Time to climb 4-stairs** is a timed function test that represents stair climbing ability – a clinically meaningful function in and of itself. It has been used as an endpoint in DMD trials for decades.[107, 108, 109] Stair climbing velocity improves up to around age 7 and then declines. It is predictive of loss of stair climbing ability, loss of ambulation and time to 10% decline in ambulatory capacity. Challenges include standardized equipment at multiple sites, sensitivity to small changes and variability that may impact sample size.
- **Myometry** provides **quantitative measures of strength** and is a biomarker of muscle function. Several measures can be used, including isometric fixed or hand-held devices or fixed isokinetic devices. Manual muscle testing (MMT) was used as the primary outcome measure to demonstrate an effect in the initial prednisone trial, but the outcome measure has a large standard deviation.[110] Hand held dynamometry is more practical and continuous variable, whereas quantitative muscle testing (QMT) requires expensive and bulky equipment. The stage of disease and mechanism of action of experimental therapeutic need to be considered for inclusion. MMT appears less sensitive and reliable in comparison

with quantitative myometry.[111] In general, children ages 5 and older may be more reliably assessed with myometry. The two muscle groups most reliably assessed in children with myometry are the knee extensors and elbow flexors.[112, 113] Myometry may be a very appropriate measure of efficacy for agents that increase or preserve muscle mass. In one recent study using a dystrophin restoration strategy in 5-6 year olds showed improved myometry strength values of knee extensors and elbow flexors.[114, 115, 116, 117, 118]

The Late Ambulatory stage (from approximately seven to thirteen years of age):
The **6-minute walk test (6MWT)** has been the most commonly used primary outcome measure in clinical development programs at the time this guidance is being written. The 6-minute walk distance (6MWD) is believed to be a global / integrated measure of multiple systems involved in walking in DMD. It correlates with stride length and cadence — validated measures of disease progression in DMD in longitudinal studies of gait pathomechanics.[119, 120] In DMD 6MWD also correlates with quantitative knee extension strength,[121] biomechanical efficiency as measured by the energy expenditure index,[122] community physical activity as measured by the StepWatch™ accelerometer[123] and gross motor skills as measured by the NSAA.[124, 125] In addition, it is a clinically meaningful measure of disease progression [126, 127] and a change of this magnitude also correlates with patient-reported measures of health-related quality of life. A ten percent or greater reduction in ambulatory capacity over 12 months has been associated with future loss of ambulation, time to a persistent 10% reduction in 6MWD has been used an outcome in DMD in one-year.[128]

The 6MWT does have some limitations, however. For instance, in younger ambulant boys with DMD, 6MWT performance may actually improve up until approximately the age of seven and perhaps later with current standard of care treatment[129]. A second challenge to the 6MWT is the floor effect of losing ambulation (defined as inability to walk 10 meters). This is an invariant feature of the disease process. Most studies have included patients who lose ambulation with values for 6MWD imputed as zero. The aggregated data analysis of change in 6MWD is strongly influenced by the inclusion or exclusion of patients who lose ambulation during the trial, which leads to imputed zero values.[130] This impact of loss of ambulation has been seen in larger cohorts over 1-3 years follow-up as well. In addition, the baseline 6MWD, using a cutoff of 350 meters is also associated with a change in 6MWD over one year with 48 week to three-year follow-up.[131, 132] Finally, it may be difficult to routinely perform the 6MWT at all potential clinical trial sites. In such settings, other outcomes, such as rise time from the floor, 10 meter run / walk, time to climb 4 stairs and other measures of functional performance, would be more appropriate in ambulant boys.

North Star Ambulatory Assessment (NSAA): On the new linearized 100-point scale of the North Star an approximate 7 to 9 point change has been deemed to be

the minimal important difference.[133] The challenge is that a recent multicenter trial demonstrated less than a 7-point decline in the linearized NSAA in placebo treated patients over 48 weeks. This may limit the responsiveness of the measure for therapies that stabilize disease progression in 48-week duration trials. Thus, the NSAA may be more useful in trials of longer duration.

Other useful motor outcome measures for late ambulatory populations recently identified include: **Time to run / walk 10 meters, time to climb and descend 4-stairs**, and more recently identified measures which include time at high step rate and number of high rate steps by **accelerometry.**[134]

Myometry. There has been a concern that lower extremity myometry reaches a floor effect in late ambulatory DMD patients and that upper extremity myometry doesn't change substantially over 48 weeks.[135] While quantitative knee extension by myometry does change in the late non-ambulant corticosteroid treated DMD patient over 48 weeks by an average of 1.85 pounds,[136] the MCID in the late ambulant DMD population has been shown to be 2.1 to 2.4 pounds.[137] Other muscle groups do not show significant changes in myometry over 48 weeks in the late ambulatory DMD population.

The non-ambulant population

This is a population in which outcome measures will need to be validated for future therapeutic trials. A number of instruments are at various stages of development. These include, the Performance of Upper Limb Scale (PUL)[138, 139] which focuses on the continuum of functionality and on basic functional workspace and is an appropriate upper extremity measure PUL; the `Motion & Function Assessment Tool' (MFAsT)[140] that uses the Microsoft Kinect Sensor, a low cost method of assessing an individual's 3-dimensional functional workspace (specifically designed for DMD, with a conceptual framework reflecting the progression of weakness and natural history of functional decline in DMD); quantitative strength testing (pinch test, grip test, elbow extensors, elbow flexors, etc.); quantitative measure of reachable workspace which measures shoulder movement; quantification of elbow, wrist and digit strength movement; the nine-whole peg test,[141] and the Egen classification (EK) scale.[142]

In addition, the Motor Function Measure (MFM),[143] a general scale developed for use across multiple neuromuscular disorders has been used in DMD. Although it is suitable for multicenter trials and has adequate reliability, sponsors should consider if the available data for comparison with other measures, the extent of natural history in DMD compared with other measures, and whether the evidence, which shows its sensitivity to change and correlation with clinical meaningfulness, are acceptable for use.

In short, rather than a defined set of outcome measure that a sponsor must use, there is a large toolbox of potential outcome measures and endpoints from which

sponsors may choose. It is the sponsor's responsibility to determine the appropriateness of the outcome measure and endpoint for the investigational drug's mechanism of action, and patient population as measured by age and functional status.

As this is a rapidly evolving area and is continuing to evolve, it is expected that the sponsors will use the most up-to-date available data for any outcome measure to ensure that they are suitable in the relevant DMD population for each trial.

However, there remains a need to develop transitional measures that can serve as a bridge across different age groups, and sponsors are encouraged to work with the FDA, the DMD community and its scientific advisors to develop novel endpoints (see below).

Pulmonary Outcome Measures and Endpoints

Pulmonary outcome measures can be divided into measures of strength, clinical measures of restrictive lung disease (which are predictive of the need for pulmonary interventions), and measures of cough function.

- Measures of strength: The maximum inspiratory pressure (MIP) and the maximum expiratory pressure (MEP) are standard clinical measures that assess muscle strength. MIP is largely the function of diaphragmatic strength, where MEP is more reflective of the strength of the rectus abdominis and oblique muscles (and to a lesser extent the intercostals). These measures are relatively independent of chest wall compliance and lung function.

- Clinical measures of restrictive lung disease: Forced Vital Capacity (FVC) is a global measure of lung function and capacity. In DMD, its clinical utility derives from thresholds of diminished function, which dictate consideration of intervention.

- Measures of cough function: Pulmonary management in neuromuscular diseases has largely been driven by findings in more common adult diseases. Measures of cough abilities such as the peak cough flow and peak expiratory flow rate have been used in these other contexts. There are positive data predicting that measuring peak flow is a potentially useful measure that correlates with quality of life in other neuromuscular diseases — although it may be difficult to use across all populations in DMD. It has been used in DMD, but without contemporary published natural history data, and thus could be considered exploratory pulmonary biomarker to measure in DMD. Clinical intervention with mechanical insuflation/exsuflation device use in DMD is driven by a threshold initially identified by clinical experience and reinforced in subsequent consensus statements.[144, 145, 146]

- Chest wall compliance and intrinsic lung function may impact peak flow measures. Use of concomitant medical therapies, including M-I/E devices and potentially even chest PT may influence peak flow measures. Sponsors should consider these potential confounding influences in measurements of pulmonary endpoints.

Cardiac Endpoints
Cardiac measures in DMD are evolving. Historically, echocardiograms have been used to assess heart health in DMD — if the echocardiogram looked normal, it was assumed that the patient with DMD was not at great risk of cardiac events. But echocardiogram is primarily a safety measure. Other measures are needed to monitor treatment response.

Pediatric cardiologists have begun to look at cardio-myopathies with cardiac MRI. In Duchenne, cardiac MRI has been used to show that damage to the heart begins quite early. Myocardial fibrosis may be observed in 17% of 6-7-year old boys with DMD.[147]

Selection of cardiac outcome measures for clinical trials is however complicated by the limited natural history data on cardiac disease in Duchenne and the non-linear relationship between decreased cardiac capacity and actual cardiac events.[148]

- Traditional echocardiography – measures of LV function (EF, dimensions, volumes) typically remain relatively stable until early teenage years, then begins to decline.
- Cardiac MRI: Measures of wall strain have emerged as earlier markers of cardiac dysfunction in boys with DMD. Declines in these markers can be detected earlier while LVEF by echo are stable. Late gadolinium enhancement to detect areas of cardiac fibrosis has detected subclinical cardiomyopathy in young boys with DMD even earlier than changes in ventricular wall motion or function are detected
- ECG: Abnormalities in ECG also evident much earlier than changes in ventricular function or dimensions as measured by echocardiogram. Whether these ECG changes are harbingers of later cardiac dysfunction or are more independent effects of the dystrophinopathy is not completely understood

At this point in time, given the lack of natural history, there is no justification at present to use any specific cardiac biomarker for accelerated approval because there are no data in a large enough cohort that would give regulators confidence that a shift as a biomarker would predict clinical improvement.

Implications for clinical trial design, sponsors should address:
- Requirements for background prophylactic cardiac medications at baseline
- Careful recording of baseline use and any change during trial

- Recommendations around minimal requirements for cardiac monitoring in trial design
    - SOC echo per care considerations
    - ECGs throughout trial: Corrected QT interval assessments need to be individualized to the MoA of a drug. Sponsors can standardize this with supplying machines and central reading sites and formulate. Depends upon the negotiation with the agency and the MoA. A protein therapeutic may not need it or it may only be useful in older populations.
    - For pharmacological interventions influencing potentially relevant pathways, or dystrophin-restoring therapies (applicable to exon-skipping, etc.…) – consider more sensitive measures for detection of earlier declines in cardiac function even if as exploratory measures only (e.g., wall strain measures by cardiac MRI or speckle tracking by echo).

Patient Reported Outcomes (PROs) in DMD

A number of PROs with existing longitudinal data have been used or are being evaluated in DMD. However, at the time of writing, none have been validated according to FDA guidance [see Patient-Reported Outcome Measures: Use in Medical Product Development to Support Labeling Claims] that details the methodology in validating PRO instruments in the disease of interest.

Sponsors are encouraged to work with academic and patient communities to develop a PRO, according to FDA guidance (see Guidance for Industry Patient-Reported Outcome Measures: Use in Medical Product Development to Support Labeling Claims), which encompasses as large a spectrum of the DMD population as possible. Health-related quality of life (QOL) instruments that are DMD-specific could also be developed, although if QOL instruments are validated in DMD, it becomes unnecessary. Also, at the time of writing, there are few tools that look at caregiver burden in DMD to address how treatment or lack of treatment affects the family; however, this may be an important are of exploration for a sponsor to consider.

A brief list of the PROs that are closest to being validated:

- The Pediatric Outcome Data Collection Instrument or PODCI, which has several domains that measure functional ability like putting on off coat.
- PROM
- PedsQL: Note, this scale moves very slowly in time, and may not be suitable for registration studies.
- NM Module
- The NeuroQol contains an extensive set of measures that are validated for neurological disorders. http://www.neuroqol.org/Pages/default.aspx
- PROM for upper limb

Development of Additional Novel Endpoints in DMD

Sponsors looking for novel endpoints in these populations could use standardized instruments that are used to assess function and demonstrate clinical meaningfulness in a variety of tools that may already be used in clinical care.

In addition, sponsors are encouraged to explore novel composite outcomes in development that assess progression of DMD across the spectrum of disease (i.e., combine components of scales such as the NorthStar with upper limb functional measures), similar to the MFM construct, and demonstrate adequate sensitivity to change in DMD.

A number of novel measures of upper limb function could be considered in non-ambulant populations, such as the '9-hole peg' test, which correlates with other measures, finger tapping and pinch/grip strength and the MyoGrip, MyoPinch and MoviPlate as measures of distal function.[149]

For older non-ambulant boys, the case can be made for finger tapping — as a function that allows a young man to control a computer mouse, is a clinically meaningful function. Some of the PRO's also include measures of distal function. Another exploratory measure being explored in natural history studies is typing time. However, the change in these measures over time is usually slow, and trials may need to follow patients for a long period of time (perhaps in an extension phase of a trial, or as part of a post-marketing commitment).

Figure 2

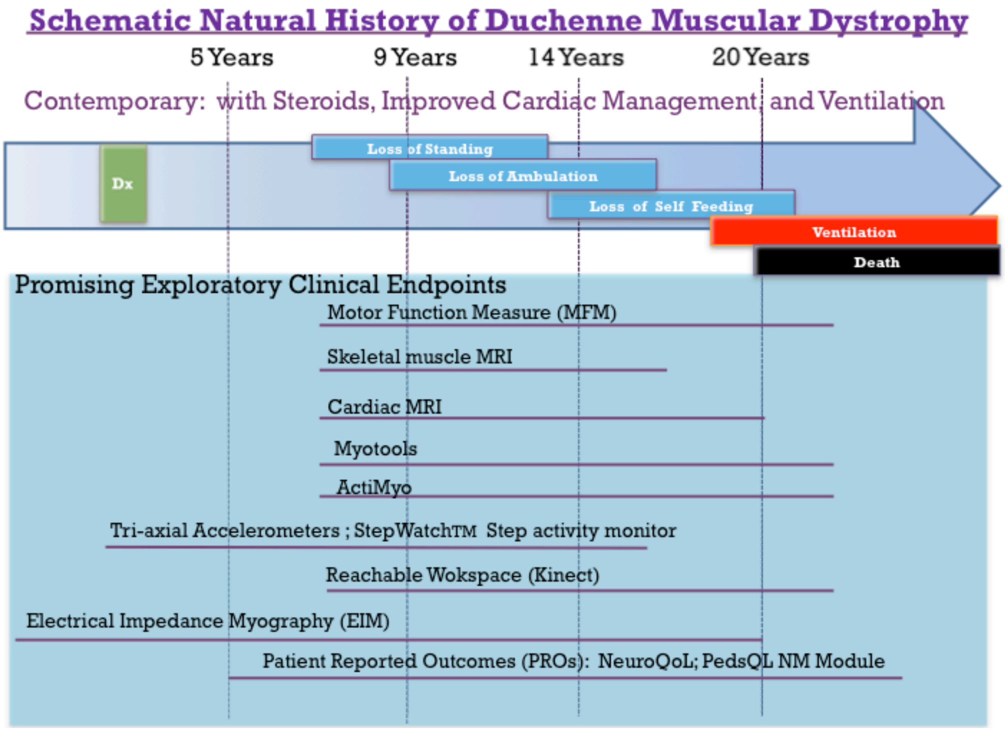

150, 151

D. Clinical Trials

Feasibility issues for trials in DMD: The limits of rare disease and medical addressability by endpoint (pool of participants and trial sites)

Sponsors are encouraged to gather safety and efficacy data of new drugs in DMD patients across the spectrum of disease. Sponsors may choose to develop separate protocols or may consider more novel trial designs encompassing more than one patient segment to collect this data.

Trial Design

There is widespread support in the DMD community to move away from placebo-controls. While the most robust data come from placebo-controlled trials, other trial designs without placebo controls may be considered if circumstances warrant.

In such cases, well-matched (age-matched and stage-matched) natural history controls would be acceptable. The challenge is to find those matches for each trial participant, from natural history data. Natural history will very heavily depend upon the standard of care being applied.

A danger, however, is that the likelihood for results that are difficult to interpret with using natural history controls is substantially greater than in randomized placebo controlled trials.

We also recognize that most sponsors are reluctant to pursue prolonged clinical development programs, and there are also concerns about tying up participants in a clinical trial of a product that, in the end, might not be efficacious.

It is the sponsor's responsibility to be familiar with the most current available data to understand the natural histories in DMD including natural history as it is modified by available therapy, and to update as appropriate for their trial design. We encourage sponsors to discuss any plans to use natural history controls with the agency.

When a placebo-controlled trial is performed, sponsors are encouraged to consider clinical trial designs that increase access to patients across the entire spectrum of disease and that limit exposure to placebo. These designs will vary depending upon the drug's mechanism of action, and the sensitivity of the specific outcomes being measured.

In addition, the focus on 6MWT as the primary endpoint for studies in DMD has limited the access to and participation in the clinical trial process as sponsors have restricted the inclusion criteria for trials to specific 6MWD ranges likely to decline but not lose ambulation.

Sponsors should use a single primary endpoint in trial designs, along with appropriate secondary endpoints. The primary endpoint may be either a clinical outcome or a pharmacodynamic biomarker depending upon the purpose of the trial.

Categorical endpoints – whilst statistically strong – may not be in the patient-community's best interest in DMD because the functions lost over the course of a trial are irreversible. Thus, the focus should be on developing more sensitive, dynamic endpoints that are either demonstrated by natural history data to be clinically meaningful or to be surrogate endpoints.

As the leading cause of death in DMD, it is critical that sponsors should consider using appropriate tools to measure safety and efficacy of compounds in the myocardia in all populations with DMD.

Novel trial designs
In order to meet the expectation of the DMD community to reduce exposure to placebo, novel trial designs, including for instance, delayed placebo or rapid roll over trials (after patients reach an non-categorical endpoint) should be explored.

One approach to minimize the time off drug in between trials in DMD studies would be to plan to proceed directly from dose escalation studies into clinical efficacy studies once a dose has been selected.

Studies with more than one experimental therapy
Guidance on conducting studies with more than one new molecular entity has previously been published [http://www.fda.gov/downloads/Drugs/GuidanceComplianceRegulatoryInformation/Guidances/UCM236669.pdf].

Standardization of measurement across trials
Because of the paucity of data in DMD (and many other) rare diseases, making data that is collected in one trial be available for use in another trial – could be critical for the entire field.

To whatever extent possible, sponsors are encouraged to harmonize how clinical trial outcomes are measured, including, potentially, the frequency with which those measures are used for efficacy – to allow interpretability across trials and across sites. It is understood however, that the ability to do so might be limited by the drug's mechanism of action and pharmacodynamics.

Use of biomarkers in DMD trials:
As Single Primary Outcome Measures
The approval of a drug for the treatment of DMD based on the use of a biomarker as a single primary surrogate efficacy measure can be considered under accelerated approval. As described in the biomarkers chapter that follows, at the present time, there is a great deal of research interest in understanding the role of biomarkers in

DMD. Some of these biomarkers appear to be pharmacodynamics markers that may be useful for proof of concept and dose selection. If the effect on a particular biomarker is reasonably likely to predict clinical benefit, the FDA would consider an accelerated approval based on the use of a biomarker as a surrogate outcome measure in DMD (see Subpart H--Accelerated Approval of New Drugs for Serious or Life-Threatening Illnesses and the Guidance Expedited Programs for Serious Conditions––Drugs and Biologics).

Use of Biomarkers as Supportive Secondary Outcome Measures
We are also open to considering the argument that a positive biomarker result (generally included as a secondary outcome measure in a trial) in combination with a positive finding on a primary clinical outcome measure may support a claim of disease modification in DMD. There is widespread evidence-based agreement in the research community that for instance, that the absence of dystrophin is fundamental to the underlying disease process in DMD. The status of approaches to measure it are discussed in the biomarkers section — as are other biomarkers that have potential as surrogate outcome measures such as skeletal muscle MRI. We encourage sponsors to analyze the results of these biomarkers independently with the understanding that these findings will be interpreted in the context of the state of the scientific evidence at the time of a future new drug application or biologics license application submission.

E. Extrapolation:
Extrapolating data of efficacy from one stage of disease to another could depend upon the mechanism of action. However, drugs designed to improve the quality and health of the muscle would be expected to benefit the patients with DMD at any stage of the disease — and thus, it may not be necessary to test a drug at every stage of the disease to justify a broader indication. Nevertheless, having some secondary endpoint data showing an effect in other disease stages could help support a broader indication.

VII. BIOMARKERS IN DUCHENNE MUSCULAR DYSTROPHY
A. General Comments
The FDA shares the Duchenne community's goal to develop biomarkers and surrogate endpoints that could provide very rapid information and data with regard to the signal of a drug and whether there is biological activity that could prove promising in terms of altering the disease course.

Sponsors should be aware that biomarker development is an aspect of DMD that is rapidly evolving. They should consider the inclusion of some of the biomarkers described in this section in their clinical development programs as endpoints or for planned post-hoc analyses, as the findings may help support an NDA for their lead product in development — and by helping move the field forward towards a consensus on the utility of a biomarker — may reduce costs and speed the time required for the development of subsequent products.

This section describes a number of biomarkers that may have prognostic or predictive values (with value in forecasting the patient's prognosis, or likelihood to benefit from a particular treatment). More attention is given to pharmacodynamic biomarkers, which provide an indication of response after being treated (sometimes in relation to pre-treatment values) — and which may have potential as surrogate endpoint that could substitute for a clinical endpoint.

The prediction is based on epidemiologic data, therapeutic, pathophysiologic or some other scientific evidence. *A surrogate endpoint biomarker* is intended to substitute for the clinical endpoint. This type of biomarker provides early and accurate prediction of both: a clinical endpoint benefit (or harm or lack of benefit or harm) and the effects of treatment on this biomarker. A 'holistic evaluation' of available data (epidemiologic data, data from clinical trials, pathophysiologic or some other scientific evidence) demonstrates that a biomarker can substitute for a clinical endpoint.[152] The designation of surrogate endpoint requires agreement with regulatory authorities.

Surrogate endpoint markers can also be the primary endpoint in 'adequate and well-controlled studies if there is a well-established relationship between the surrogate marker and clinical outcome then that trial can be used to provide evidence for conventional marketing approval. If on the other hand, there is not a well-established relationship between the surrogate marker and clinical outcome but it is 'reasonably likely' to predict a clinical outcome, then a positive effect on the surrogate endpoint could lead to an accelerated approval.

In DMD, biomarkers that faithfully report on both the health and amount of skeletal muscle may potentially be useful at different stages of the clinical trial process as prognostic, predictive, or pharmacodynamic biomarkers.

The chapter is split into two sections: one looking at biomarkers found in muscle tissue. The biopsy-based biomarkers used to date in DMD trials – dystrophin and utrophin – are widely accepted by the scientific field as appropriate pharmacodynamic biomarkers for therapies whose mechanism of action is directed toward their expression, as they may confirm the mechanism of action and be useful for selecting doses in subsequent trials, and (in the case of dystrophin in particular), from natural history studies, it is clear that patients who express dystrophin (BMD) do better than DMD patients, and even DMD patients who express very low dystrophin levels appear to have a slower disease progression.[153] At the time of this writing, data are being analyzed regarding the potential of measures of dystrophin as a predictive and surrogate endpoint that could support applications for one of the expedited approval processes. Biopsy-based biomarkers may nonetheless be unattractive for use in large phase III studies due to the invasive collection method.

The second section looks at less invasive methodologies to measure changes in the muscle and new milieu. Some of these, such as measures of proteins, protein fragments and genetic materials in the blood or urine are exploratory but worth

greater investments due to the ease with which they can be measured. Some imaging techniques are much further along. In particular, the direct imaging of skeletal muscle using MR techniques has the potential to serve as an efficacy-response biomarker and surrogate endpoint.

Sponsors should be aware that scientific consensus regarding the utility of any of the exploratory biomarkers may have been reached since the time of writing — and should discuss this with the FDA. In the meantime, however, evidence of an effect on an exploratory biomarker could provide supportive evidence for a claim of disease modification in an NDA. When combined with some other evidence suggestive of clinical benefit, sponsors could help establish the use of a biomarker as a surrogate endpoint.

B. Muscle Biopsy Biomarkers: Dystrophin And Utrophin

General comments

Dystrophin organizes and stabilizes the sarcolemma to effectively distribute contractile forces and maintain myofiber structural integrity and function. Dystrophin is also a scaffolding protein necessary for the proper localization (and thus function) of signaling molecules such as neuronal Nitric Oxide Synthase (nNOS). The use of dystrophin in muscle as a diagnostic (and prognostic biomarker) has already been discussed in the diagnosis chapter.

The accurate quantification of dystrophin or utrophin in muscle tissue can provide important support for the clinical development of dystrophin- or utrophin-restorative therapies. Evidence of a significant effect on dystrophin levels, for instance, could provide proof of concept that a dystrophin-restorative therapy does in fact increase dystrophin production.

However, to measure either dystrophin or utrophin content in the target muscle accurately, one must first address specimen collection — how the muscles are biopsied. In addition to technical issues related to how the muscle sample is collected and processed, there are considerations regarding the ethics and reliability of the process.

Considerations related to muscle biopsies

Sponsors should be aware of considerations related to the measurement of a biomarker in a muscle biopsy — for instance, interpreting the relative quantification of dystrophin with the tools available today requires pre-treatment /self-patient controls. Sponsors should consider issues related to specimen collection, handling, and laboratory practice and take care to minimize sampling errors.

Ethical concerns of biopsies in children

Sponsors should be aware that there are ethical issues related to performing multiple muscle biopsies in patients with a degenerative neuromuscular disorder. Bearing in mind the invasiveness of the procedure, sponsors should consider whether biopsies are absolutely necessary when planning a clinical trial in order to

minimize their use. If a biopsy is required, the greatest care should be taken make certain that the biopsy provides a useful specimen and that the timing post treatment is appropriate. Trials performing muscle biopsies should commit to timely feedback about the biopsy analysis to the trial participants, and DMD community.

Criteria of what is an appropriate biopsy for dystrophin or utrophin quantification

Site of biopsy/muscle group: At baseline, the amount of dystrophin varies by donor, mutation, and muscle group. For this reason, baseline muscle biopsies are essential in documenting changes induced by novel therapeutic agents targeting dystrophin or utrophin. Determination of dystrophin or utrophin content depends on the method utilized, and the denominator used (e.g. total protein or RNA content, myofibrillar protein content, unit membrane area). Many DMD patients show revertant fibers (endogenous clonal exon skipping), varying by mutation and muscle group. The biopsy sites should be chosen to maximize the information on dystrophin or utrophin expression pre-and post-treatment.

Note: dystrophin levels vary between different muscle tissues (so ideally the reference healthy sample should be from the same muscle as the trial biopsy).

The handling of the biopsy: *This methodology represents agreed upon good practice at the time of writing this document. However, sponsors are encouraged to utilize the best current methodology at the time of conducting their trial.* [154]

- Tissues should be flash/snap frozen in isopentane cooled in liquid nitrogen soon after surgery.
- Care should be taken to avoid the use of tissue-embedding media that compromise biochemical analyses involving gel electrophoresis (immunoblots, mass spec).
- Flash-frozen tissues should be stored in pre-chilled (dry ice), small airtight screw top tubes. Hydration of the container (including ice frozen in bottom of tube) may prevent dessication artifact (freeze drying) with extended storage.
- Shipment and transport with temperature monitoring of biopsies from clinical sites to laboratory of analysis. Great care should be made in selecting the courier confirming their expertise in low temperature controlled shipments and have significant demonstrable history of shipping clinical materials.
- Samples must not be allowed to thaw at any point, as freeze-thaw cycles decrease intact dystrophin or utrophin content as an artifact.
- Lab qualification issues: Sponsors should only utilize laboratories that are qualified to handle muscle biopsies.

Minimizing variability and sampling errors

- Sponsors should be aware that a potential limitation of muscle biopsies and quantitation of dystrophin or other myofiber proteins can be the age-related

replacement of muscle with variable fibrosis and fat in DMD patients. Dystrophin is only expressed in myofibers, and the gradual age-related loss of myofibers in DMD patient muscle complicates the interpretation of dystrophin rescue. However, the intent of the above mentioned procedures are to help minimize such complications.

- Some experts have proposed the use of imaging to guide the biopsy to make sure that the specimen contains an adequate sample of myofibers rather than fibrotic tissue.
- Muscle biopsies from both Becker muscular dystrophy and female DMD carrier patients and DMD patients often show variability in expression of dystrophin both in neighboring myofibers, between different regions of the same biopsy, and between different biopsies. Histopathology can also be variable within these same biopsies.
- Therefore, quantification of dystrophin expression in DMD biopsies requires rigorous protocols with adequate controls, extremely careful sample handling, and careful examination of a large number of fields with myofiber counting and grading by experienced pathologists or readers blinded to the treatment assignment of the patients. Similar issues with variability in utrophin staining should also be expected due to differing regions of myofiber regeneration.
- Sponsors should be familiar with the most current methods to minimize variability and sampling errors when evaluating biochemical efficacy in clinical trials.

Dystrophin Analyses

Broadly disseminated techniques

At present, the two most commonly used methods to quantify dystrophin are immunofluorescence or immunohistochemical analysis and Western Blot. Immunofluorescence can be used to determine the percentage of muscle fibers that express dystrophin and the levels at which dystrophin is expressed in these fibers. Western Blot can show the total amount of dystrophin in the specimen and its size. The methods are complementary and protocols that allow standardization of the methodologies across laboratories have also now been published. While neither technique provides a complete account of dystrophin restoration, both methods can show increases of dystrophin expression over baseline.

Immunofluorescence or immunohistochemical analysis by type: many pathology laboratories routinely employ this method. However, the necessary methods to quantitate dystrophin in a manner able to support clinical trials and drug development are not broadly disseminated. *Standardization of immunofluorescence methods across laboratories has not been widely established yet. However, protocols that allow standardization across laboratories have now been published [Anthony et al, Neurology 2014 TKTK).*[155] *The advantage of immunohistochemical methods is that they examine relative levels of dystrophin and correct localization at the sarcolemma. This method is also more sensitive than*

western blotting. [Publication pending] Immunostaining quantitation of relative dystrophin levels should be done by specific referral laboratories with extensive documented experience with dystrophin quantitation methods and demonstrated reproducibility (intra assay (between sections) of a biopsy and inter assay precision — between experiments).

Western Blot: Western Blot is a standard method of quantifying the amount and size of a protein. However, it needs to be recognized that dystrophin is a large molecular weight (427kD), low abundance protein. There are frequently encountered technical challenges with consistency and reliability of multiple steps of the protocol, including solubilization, electrophoresis, transfer (blotting), immunodetection, and quantitation.

Methodology: Protein solubilization. One frequently published method is to use cryosections (lacking any embedding media; 20-50 10 micron) collected in pre-chilled small tubes, with rapid solubilization in low volume high SDS buffer, immediate electrophoresis on gradient Tris-acetate gels, and normalization of dystrophin content to myofiber proteins in the same blots or post-transfer gels.

Standardization: Protocols to standardize immunoblotting for dystrophin across laboratories are also published in Anthony et al. [Publication pending]

Emerging technologies

Mass spectrometry: Mass spectrometry methods show potential advantages of high reliability accuracy, and sensitivity. Mass spectrometry methods typically require the addition of stable isotope labeled peptides to the solubilized human muscle sample. One recently reported exploratory method uses stable isotope labeled mouse muscle mixed with human muscle biopsy samples, leading to highly accurate and reliable quantitation of dystrophin over a large dynamic range.[156] This method requires that the DMD patient dystrophin being analyzed be of a similar molecular weight as normal dystrophin (e.g. this method is not applicable to the mini- or micro-dystrophin constructs utilized in gene therapy approaches).

Benchmarking to immunoblot and immunostaining has been done in pre-clinical trials of exon skipping, and has shown concordance between all methods.[157] The major distinctions are that the reliability of the mass spec method appears considerably better than immunoblotting or immunostaining, due to the many multiple quantitative measures (peptides) per test, and the high resolution and quantitative precision of the mass spectrometers.

Use as a primary dystrophin measure: At the time of writing, there have yet to be published reports using mass spectrometry approaches in the context of a dystrophin replacement clinical trial, which would be necessary to validate the methodology.

Current limitations for all methods

All currently used and developed methods allow only relative quantitation and not absolute quantification of dystrophin levels. Since dystrophin levels vary between healthy individuals, using the same control reference sample is necessary to extrapolate relative quantitation.

Reference ranges and outliers
Dystrophin and exon skipping should be compared within an individual using the same muscle groups in a pre and post treatment biopsy. Dystrophin quantification must be done in a blinded manner by comparison to a baseline biopsy in order to be valid.

As methodologies for dystrophin quantitation are refined and widely accepted, it should become feasible for dystrophin quantitation to be presented as a percentage of normal control muscle samples, analyzed in parallel. Sponsors are encouraged to consider methodologies that allow for standardization. Ideally, the percentage of positive fibers as well as the relative dystrophin levels should be assessed.

Use of dystrophin quantification or relative quantification as a biochemical outcome measure
The amount of dystrophin restoration necessary to achieve clinical benefit is unclear at present, and may depend upon the disease stage at treatment initiation and state/health/ fragility of the muscle.

It has been established that dystrophin levels correlate with the prognosis seen in female DMD carriers[158] (normal dystrophin[159]), and in male Becker muscular dystrophy (abnormal but at least partially functional dystrophin).[160] While the amount of dystrophin restoration that can be achieved therapeutically is yet to be seen, the broad consensus is that similar levels of dystrophin restoration would be likely to result in some clinically meaningful benefit. However, the correlation is unlikely to be perfect between what may be seen as a result of therapeutic *de novo* dystrophin introduced in DMD patients later in life and what has been reported in female carriers and patients BMD, where some dystrophin is present from birth. The therapeutic benefits of dystrophin restoration may depend upon the age at treatment initiation, the health of the muscle in the patient receiving treatment and/or other factors. Nonetheless, in a medically addressable population, some degree of dystrophin restoration is reasonably likely to result in some clinical benefit, although the effect size and timing of clinical response are unclear at the time this guidance is being written.

Utrophin Analysis
The methodologies employed to quantify the expression of utrophin or associated proteins such as the sarcoglycans and nNOSs closely mirror those described above for dystrophin.[161] However, utrophin analysis also presents several unique challenges:

- The most significant complication with staining for utrophin changes after therapeutic intervention is the very high level of utrophin staining seen in

DMD and to a lesser extent, Becker biopsies. Most fibers in DMD biopsies will be utrophin positive highlighting the massive number of regenerating fibers seen at any one time within a biopsy. Therefore a combination approach should be considered both quantifying utrophin levels and quantifying the numbers of regenerating fibers. A utrophin modulation approach should at least have the same level of utrophin as the pre-biopsy but the numbers of regenerating fibers decreased over the therapy dosing to confirm the utrophin identified is due to the drug rather than regeneration.

- The utrophin abundance in normal skeletal muscle is 1-2% of dystrophin and therefore may only be useful as a baseline for quantifying fold-increase in response to up regulation therapies.
- The use of normal fetal muscle biopsies available from commercial biobanks will contain significantly more utrophin with most, if not all fibers, positive so can be used as a positive control for staining.
- Expression/purification of recombinant human utrophin is feasible and would support absolute quantification by Western blot against a standard curve. However it is not clear if endogenous and recombinant human utrophin are similarly post-translationally modified, or how any differences in post-translational modification may impact immunoreactivity.
- Utrophin expression is prevalent in regenerating skeletal muscle fibers, the vasculature and nerves of normal skeletal muscle and non-muscle cells of the immune and fibrotic responses to dystrophinopathy, which complicates interpretation of Western Blot analysis. Immunofluorescence analysis can address this issue.
- While Western Blot quantification for upregulation of other DGC components may provide some assurance of utrophin upregulation in skeletal muscle fibers, the utrophin expressed in regenerating fibers interacts with DGC components. Some of the DGCs that interact with utrophin are only present in muscle (e.g. alpha- and gamma-sarcoglycan and nNOS), thus Western Blotting could give information about the muscle-specific DGC.
- At the time of writing, no utrophin-null human tissue is available for background correction either Western Blot or immunofluorescence based quantifications, although the highly restricted localization of utrophin to the neuromuscular and myotendinous junctions, vasculature and nerves of normal muscle leave large regions of muscle sections amenable to background correction.
- It is unknown whether baseline levels of utrophin are stable in the patient, and the available data suggest that it increases with age.[162]
- Mass spectrometry methods may also be applicable to quantification of utrophin, but at the time of this writing, this work is very preliminary.

Muscle Biopsy Biomarkers: RT/RNA PCR analysis for exon-skipping detection to confirm mechanism of action in the exon-skipping field

Duchenne muscular dystrophy is mostly caused by mutations in the *DMD* gene that lead to a reading frame shift and premature translation termination. Antisense

oligonucleotides [AONs] have been designed to promote exon skipping during splicing of dystrophin pre-mRNA, restoring the reading frame and allowing translation of internally truncated, but functional dystrophin protein. A commonly used parameter to assess and compare the efficacy of various antisense molecules is the exon skipping percentage, which is defined as the percentage of transcripts in which the targeted exon is skipped relative to the total number of dystrophin transcripts (skipped vs. non skipped). There appears to be a correlation between exon-skipping percentages and dystrophin restoration, taking into account that quantification by both methods has only been achieved by highly specialized centers. Hence the measurement of exon skipping at the RNA level is an important assessment in verifying AONs' ability to successfully modify the appropriate gene target. Due to the low abundance of dystrophin mRNA, the efficacy of AONs to induce exon skipping has predominately been assessed at the transcriptional level using the semi-quantitative nested reverse-transcription polymerase chain reaction (RT-PCR) or quantitative PCR (qPCR) with differing protocols and amplification cycles.

Because of the different dynamics of transcripts and proteins, the exon skipping levels may not directly correlate to dystrophin levels. Nevertheless, this is another pharmacodynamic marker that can confirm the whether exon skipping has at least occurred after treatment with AONs. At the time of drafting this document various groups have been investigating to develop a quantitative method to measure exon skipping more accurately.[163,164, 165,166]

B. Non-Biopsy Based Biomarkers

General comments

This sub-section deals with two classes of exploratory biomarkers, substances that can be measured in the blood and urine, and non-invasive imaging techniques. Both classes of biomarkers in development could have considerable advantages over muscle biopsies in that they sample large groups of muscles, and thus do not suffer from the sampling errors that can be encountered with muscle biopsies, particularly if adequate care is not taken following appropriate procedures. Muscle biopsies are appropriate in circumstances where the goal is to determine if the therapeutic is acting via the intended target mechanism and in circumstances where there is no other manner in which to determine the optimal dosing. While at the time of writing, we recognize that sponsors may need to rely upon established methodologies in their registrational studies, we would also encourage them to explore the use of less invasive biomarkers in their clinical development programs.

Serum and Urine Biomarkers

Sampling blood and urine in DMD may indicate the health and integrity of skeletal muscles. Biomarkers in the blood or urine potentially contain signals coming not only from the affected muscles, but also from other cells involved in response to the muscle damage, including inflammatory cells and motor neurons. The blood and urine biomarkers that are coming from skeletal muscle suffer from the fact that since they reflect the amount of skeletal muscle as well as the health and integrity of

the muscles, they must be corrected for loss of skeletal muscle mass as the disease progresses. The blood biomarkers that have been explored to date include both protein and RNA, while urine biomarkers are primarily metabolites.

Proteins and protein fragments
Proteins can measured by multiple methods for 'biomarker discovery' including immunological methods (antibodies), proteomics methods, or aptamer panels (e.g, the Somalogics Platform). A number of potential protein biomarkers for DMD have been identified in human trials. Some appear to be similar of CK, flagging the deterioration of muscle seen in DMD. But a number of others that may provide additional information (including response to treatment) have recently been identified.

A number of ongoing studies are utilizing the SomaLogic platform, which involves proteomics tools, including modified aptamer protein-binding reagents with high affinity and slow dissociation rates that target thousands of proteins critical to biological functions as well as an assay that can simultaneously identify and quantify proteins across approximately eight logs of concentration in small sample volumes.[167,168,169] Since the modified aptamers are composed of DNA, established DNA measurement technologies can be used to quantify them and provide a readout of thousands of proteins from a small amount of biological sample.[170] A potential advantage of the platform is that it samples rare proteins in addition to abundant proteins. Therefore, it may be possible to detect changes in relevant pathways that might be missed by other platforms including proteins that are indicative of denervation that are never seen in the normally functioning peer group.

Another platform under evaluation in Duchenne are Nordic Bioscience's fibrosis biomarkers assays, which identify specific protein fragments, or 'neo-epitopes' produced when proteins are subject to post-translational modifications (PTMs), e.g. cleavage, glycosylation or citrullination, that are related to defined (patho)physiological processes during morphological deterioration.[171, 172, 173, 174] The resulting specificity between the parent protein and the relevant PTM gives rise to modified peptides that are associated with specific (patho)physiological processes in cancer, fibrosis, or neuromuscular degeneration.

Other studies have looked at a range serum or urine protein and protein fragments in DMD include Matrix metallopeptidase 9 (MMP-9) in DMD[175], fibronectin[176], muscle protein fragments in serum and urine, succinate in *mdx,* prostaglandin D2,[177] and3-methyl-L-histidine[178]. Some proteins may be markers of the disease repair process and tissue remodeling.

At the time of writing this guidance, all of these biomarkers are exploratory, however, sponsors are encouraged to screen for these potential biomarkers in longitudinal studies. With such data, the potential utility of some of these markers to monitor treatment response may become more apparent.

MicroRNAs

MicroRNAs (miRs) are short (~22 nucleotide) RNA molecules that function in the post-transcriptional regulation of gene expression by inducing mRNA degradation or translational inhibition. A set of miRs, called dystromirs[179, 180, 181] have been identified in the serum of Duchenne muscular dystrophy (DMD) patients, as well as that of that of DMD animal models, at copy numbers that are significantly different from healthy subjects or control animals. Dystromirs may have advantages over proteins or metabolites as serum biomarkers. Quantitative RT-PCR serves as a rapid, sensitive and accurate method of detection of these small RNA molecules. Since they may be actively exported from muscle cells, serum levels of dystromirs could be less sensitive to the effects of physical activity than CK. At the time of writing, further data are required in longitudinal treatment studies to determine whether dystromirs would be effective tools in monitoring response to treatment.

Recommendations regarding serum and urine biomarkers:

Sponsors should endeavor to collect serum and urine specimens at time points during the trials with the appropriate ethical agreement for use in future biomarker development as research materials. Potentially any samples eventually identified to be samples from the placebo group could be biobanked and be made available to assist other entities developing new biomarkers.

Imaging modalities

An inherent deficit in dystrophin-deficient muscle is increased membrane fragility that renders dystrophic muscle more susceptible to contraction-induced injury.[182, 183, 184, 185, 186, 187, 188, 189] Dystrophic skeletal muscle in humans undergoes repeated cycles of muscle fiber injury, degeneration, and regeneration. The pathological progression of DMD includes increased sarcolemmal permeability, muscle inflammation, and ultimately myofibers are progressively replaced by fat and connective tissue. The pathological progression is relentless but highly variable within and across individuals, both spatially and temporally, with some muscle groups rapidly progressing while others are relatively preserved late in the disease. Imaging this progressive replacement of muscle with fat and fibrosis can assess the current status of the patient, and when combined with natural history data on the rate of progression of muscle replacement, imaging can allow assessment of the impact of interventions.

Ultrasound (US) is a non-invasive imaging technique that can provide rapid anatomical and functional measurement of human tissue, and places low demand on the subject.[190] As such it is well suited for pediatric imaging. US imaging has been extensively applied to investigate cardiac abnormalities associated with DMD. Muscle atrophy and intramuscular fibrosis and fatty infiltration can be visualized using US of skeletal muscle.[191, 192] US density analysis of skeletal muscle provides a sensitive method for distinguishing between healthy children and children with neuromuscular disorders.[193] Quantitative muscle ultrasound has been applied to study DMD by quantifying echo intensity and muscle thickness. A significant increase of echo intensity with age, reflecting increasing dystrophic muscle changes,

was observed. This increase was related to ambulatory status, functional grading, muscle strength and motor ability.[194]

Electrical impedance myography (EIM) provides a non-invasive approach for quantifying tissue composition and compartmentation and as such has relevance for assessment of neuromuscular disease pathology. EIM 50 kHz phase measurements have been reported to correlate well with standard functional measures in DMD; NorthStar Ambulatory Assessment test ($R = 0.83$, $p = 0.02$).[195]

DEXA is a technique that can be used to estimate body composition including bone mineral density and body lean soft tissue and indirectly provides an estimate of fat content. Studies of DEXA in DMD subjects have found decreased regional lean mass, increased regional fat mass, and decreased strength — but DEXA cannot distinguish between muscle and fibrosis.[196] Nevertheless, there may be a role for DEXA to help normalize muscle mass for the accurate measurement of serum biomarkers.

MRI and MRS, magnetic resonance imaging and spectroscopy, provide the most detailed and quantitative information as to the status of individual skeletal muscles. Healthy muscle can be distinguished from diseased muscle, and the infiltration of fat and fibrosis can be monitored and quantified. The primary limitation of MR approaches is that they are costly and the evaluation is time consuming, as compared to other imaging approaches. Nonetheless, the power of MRI/MRS will make it increasing important for DMD studies. Combining MR with more frequent follow-ups using less expensive approaches such as ultrasound or EIM may ultimately provide the best compromise.

Magnetic resonance imaging (MRI) is the modality of choice when high-resolution/high contrast images of soft tissue are demanded. MRI is a non-invasive technique that does not use ionizing radiation, provides outstanding volumetric coverage of tissue, instruments are widely available, and the technique can be run quantitatively and standardized across sites.[197] Magnetic resonance spectroscopy (MRS) is a class of techniques used to measure the biochemical properties of tissue. The fundamental hardware required for MRS is identical to that used in MRI, which makes MRS a high-value ancillary study to MRI. A fundamental strength of MRS is the increased specificity for measurement of distinct tissue constituents. An example relevant to DMD is the high-fidelity separation of tissue water and fat signals, which typically co-contribute to standard MRI signals collected from skeletal muscle of DMD individuals. MRS techniques have been applied to investigate cellular metabolites typically using the most abundant magnetic isotopes of hydrogen (^{1}H), carbon (^{13}C), and phosphorus (^{31}P). MRS has been used to improve diagnosis, to better define the natural history of a disease process, and in some studies to monitor the response to therapy.[198, 199, 200, 201, 202, 203, 204, 205, 206, 207] While most MRI/MRS investigations of DMD skeletal muscle have focused on the lower extremities, investigation of shoulder and upper extremity muscle is also feasible and underway.[208, 209] Finally, the fact that that MRI/MRS measures are obtained with the subject at rest, greatly reduces the impact of motivational issues

that confound many functional measures. Taken together, these attributes make MRI/MRS attractive techniques for longitudinal investigations of rare disease in human pediatric subjects.

MRI/MRS: Emerging Biomarkers of Human muscular dystrophy Pathology
Numerous studies have demonstrated the ability of MRI to detect alterations in skeletal muscle structure in patients with muscular dystrophy.[210, 211, 212, 213, 214, 215, 216, 217, 218, 219, 220, 221] Indeed, due to its excellent soft tissue 3D imaging capability and the ability to perform longitudinal measures of muscle mass, MRI has been used in clinical trials to quantify changes in skeletal muscle volume following treatment with, either a neutralizing antibody to myostatin or following myoblast transplants.[222, 223] However, most MRI investigations have relied on T_1- weighted images and the contrast generated by fatty tissue infiltration to visualize the pattern of muscle involvement in muscular dystrophy patients.[224] Radiologists have developed a four-point grading system to categorize disease severity, based on visual inspection of fatty tissue infiltration.[225] This strategy was recently used to screen DMD subjects prior to injection with antisense oligonucleotides.[226] However, few MR studies have presented a robust quantitative approach to monitor disease progression in DMD, limiting the viability of MR as a sensitive surrogate outcome measure for clinical trials.

Spectroscopy and spectroscopic imaging have been well established as noninvasive quantitative biochemical assays. A number of studies have found strong correlations between intramuscular lipids and measures of functional ability.[227, 228, 229, 230, 231, 232, 233, 234, 235, 236, 237, 238, 239, 240, 241, 242, 243] A recent study evaluated the effects of corticosteroid initiation in in DMD steroid naïve patients using MRI/MRS found a significant decrease in T_2 (the transverse relaxation time constant) that occurred prior to any measureable functional improvements.[244] This suggests that MRI/MRS responses to therapies may be more sensitive to treatment effects and may even be predictive of future functional improvements.

<u>MR Imaging of Inflammation</u>
MR imaging strategies are also sensitive to muscle inflammation regions of increased signal intensity in muscles of young boys with DMD in the absence of fatty tissue infiltration.[245] This finding is consistent with inflammation occurring early in DMD, prior to the loss in contractile tissue and accumulation of fatty tissue. The importance of inflammation in DMD is further supported by the 100 fold higher serum levels of TNF-α that is detectable in boys with DMD compared to controls (27.8 ng/L vs. 0.27 ng/L).[246] Changes in the serum levels of TNF-α with age are consistent with the observations that muscle expression of TNF-α and IL-6 decreases with age.[247] A direct relationship between inflammatory markers (serum and tissue) and hyper intensity observed on STIR images has been documented in FSHD.[248]

Another MR imaging modality, Na+ imaging, showed that areas of hyper intensity on STIR images from skeletal muscle in DMD subjects are directly related to muscle

edema.[249] Taken together, these findings indicate that MR sequences may be sensitive to early inflammation in the dystrophies.

Other investigations have focused on imaging strategies that are sensitive to muscle damage and inflammation to visualize dystrophic lesions, which may be particularly important in younger boys with DMD. Using short-tau inversion recovery (STIR) sequences, it is possible to identify regions of increased signal intensity or muscle inflammation in dystrophic muscles of young boys with DMD in the absence of fatty tissue infiltration. T_2 weighted imaging has been implemented to visualize dystrophic lesions and found increased signal intensity in both young and older ambulatory boys with DMD, with muscle regions of elevated T_2 ranging from 1% to 94%. The most challenging part in implementing magnetic resonance to characterize dystrophic human muscle lies in the assessment of muscle damage/inflammation, in the presence of large amounts of fatty tissue infiltration. However, the ability to monitor muscle damage *in vivo* is extremely important, since the primary target of most gene therapy or pharmaceutical interventions is the restoration of the expression of structural proteins and normal sarcolemmal integrity. Preliminary data in both murine models of muscular dystrophy and boys with DMD demonstrate the feasibility of implementing T_2 weighted imaging and ^{1}H-spectroscopic relaxometry to study dystrophic muscle.

MR Imaging of Fibrosis

A significant challenge for MR and other noninvasive imaging modalities is the quantification of fibrosis. The observed MR signal intensity associated with fibrosis undergoes a characteristic rapid decay due to the extremely short T_2s of water molecules associated with collagen.

Cardiac MRI studies in DMD subjects have reported an age related decrease in myocardial T_2 compared to controls[250, 251] and an increase in myocardial T_2 heterogeneity[252]. Similar results have been observed in animal models with diabetic induced cardiac fibrosis.[253, 254] The decreased T_2 has been hypothesized to represent an increased fraction of water molecules “bound” to collagen and other fibrotic tissue. Similarly, MRS relaxometry data acquired in the parent project show an age dependent decrease in muscle water T_2 in both calf and thigh skeletal muscles in boys with DMD, but not healthy controls. This decrease in T_2 is typically masked in skeletal muscle imaging by the large amounts of fatty tissue deposition (preliminary results).

MRI/MRS: Role in Clinical Trials

Numerous studies have now shown the ability of MRI/MRS to visualize structural alterations in skeletal muscle of patients with muscular dystrophy[255, 256, 257, 258, 259, 260] yet MR has been incorporated in only a handful of DMD clinical trials. However, the emerging data supports a much-expanded future utilization of MR imaging in DMD trials. Since MRI/MRS outcomes faithfully report on both the health and amount of skeletal muscle, they can potentially be used at different stages of the clinical trial process as prognostic, predictive, or pharmacodynamics biomarkers.

Combining MR/MRS outcome measures with functional outcomes in treatment trials could lead to the demonstration that MRI/MRS measures can serve as an efficacy-response biomarkers and surrogate endpoints to accelerate clinical development.

IX. **CONCLUSION**

We believe that DMD would be an appropriate indication to use some of the more flexible measures for a regulatory filing as set forth in FDASIA. These measures have recently (in 2014) been described, in Guidance for Industry*: Expedited Programs for Serious Conditions – Drugs and Biologics*.

In the case of DMD, at the time of writing, sponsors may take one of several approaches towards an expedited approval pathway. For instance, clear evidence of a response to treatment in certain biomarkers — such as evidence of dystrophin where previously there was none, or evidence of a change in the health or rate of deterioration of skeletal muscle on MRI/MRS — could be supportive of accelerated approval for a product — particularly if combined with intermediate clinical endpoint data,, or, potentially, PRO data suggesting that trial participant's have experienced meaningful clinical benefit.

Similarly, preliminary evidence of early clinical benefit — such as a significant change in time to function tests or 6MWT — could merit a breakthrough designation for a product. Sponsors are, however, encouraged to include exploratory biomarker measures as secondary endpoints in their trials as well, in order to foster biomarker discovery and to increase the database to support the use of those markers as pharmacodynamic or surrogate endpoints in future trials.

REFERENCES

[1] Mendell, JR, Schilling C, Leslie, N et al. Evidence-Based Path to Newborn Screening for Duchenne Muscular Dystrophy. ANN NEUROL 2012;71:304–313.

[2] Moat SJ, Bradley DM, Salmon R et al. Newborn bloodspot screening for Duchenne muscular dystrophy: 21 years experience in Wales (UK). Eur J Hum Genet. 2013 Oct;21(10):1049-53. doi: 10.1038/ejhg.2012.301. Epub 2013 Jan 23.

[3] Peay HL, Hollin I, Fischer R, Bridges JF. A community-engaged approach to quantifying caregiver preferences for the benefits and risks of emerging therapies for duchenne muscular dystrophy. Clin Ther. 2014 May 1;36(5):624-37. doi: 10.1016/j.clinthera.2014.04.011.

[4] Moat SJ, Bradley DM, Salmon R et al. Newborn bloodspot screening for Duchenne muscular dystrophy: 21 years experience in Wales (UK). Eur J Hum Genet. 2013 Oct;21(10):1049-53. doi: 10.1038/ejhg.2012.301. Epub 2013 Jan 23.

[5] Guo JJ, Pandey S, Doyle J et al. A review of quantitative risk–benefit methodologies for assessing drug safety and efficacy—report of the ISPOR risk–benefit management working group. Value Health 2010:13, 657–666.

[6] Lynd LD, O'brien BJ. Advances in risk-benefit evaluation using probabilistic simulation methods: an application to the prophylaxis of deep vein thrombosis. J Clin Epidemiol. 2004 Aug;57(8):795-803.

[7] Levitan B. A concise display of multiple end points for benefit-risk assessment. Clin Pharmacol Ther. 2011 Jan;89(1):56-9. doi: 10.1038/clpt.2010.251. Epub 2010 Nov 24.

[8] Coplan PM1, Noel RA, Levitan BS et al. Development of a framework for enhancing the transparency, reproducibility and communication of the benefit-risk balance of medicines. Clin Pharmacol Ther. 2011 Feb;89(2):312-5. doi: 10.1038/clpt.2010.291. Epub 2010 Dec 15.

[9] Noel R, Hermann R, Levitan B et al. (2012) Application of the Benefit-Risk Action Team (BRAT) Framework in Pharmaceutical R&D: Results From a Pilot Program Drug Information Journal 46(6): 736-743.

[10] Yuan Z1, Levitan B, Berlin JA. Benefit-risk assessment: to quantify or not to quantify, that is the question. Pharmacoepidemiol Drug Saf. 2011 Jun;20(6):653-6. doi: 10.1002/pds.2149.

[11] Walker S, Liberti L, McAuslane N, Levitan BS. Refining the benefit-risk framework for the assessment of medicines: valuing and weighting benefit and risk parameters. Clin Pharmacol Ther. 2011 Feb;89(2):179-82. doi: 10.1038/clpt.2010.290.

[12] Hauber AB, Fairchild AO, Johnson FR. Quantifying Benefit–Risk Preferences for Medical Interventions: An Overview of a Growing Empirical Literature. Applied Health Economics and Health Policy. August 2013, Volume 11, Issue 4, pp 319-329.

[13] Hauber AB, Fairchild AO, Johnson FR. Quantifying Benefit–Risk Preferences for Medical Interventions: An Overview of a Growing Empirical Literature. Applied Health Economics and Health Policy. August 2013, Volume 11, Issue 4, pp 319-329.

[14] Joy M, Little E, Maruthur N et al. Patient preferences for the treatment of type 2 diabetes: A scoping review, Pharmacoeconomics, 2013:31(10): 877-892.

[15] Bridges JFP. Stated preference methods in health care evaluation: An emerging methodological paradigm in health economics. Applied Health Economics and Health Policy 2003;2(4):213-24..
[16] Bridges JF, Kinter ET, Kidane L et al. Things are Looking up Since We Started Listening to Patients: Trends in the Application of Conjoint Analysis in Health 1982-2007. Patient. 2008 Dec 1;1(4):273-82. doi: 10.2165/01312067-200801040-00009.
[17] Ryan M, Gerard K. Using discrete choice experiments to value health care programmes: current practice and future research reflections. Appl Health Econ Health Policy. 2003;2(1):55-64.
[18] Marshall D, Bridges JFP, Hauber B et al. Conjoint analysis applications in health: How are studies being designed and reported? An update on current practice in the published literature between 2005 and 2008. The Patient: Patient-Centered Outcomes Research 2010;3(4):249-56.
[19] Hole AR. Modelling heterogeneity in patients' preferences for the attributes of a general practitioner appointment. J Health Econ. 2008 Jul;27(4):1078-94. doi: 10.1016/j.jhealeco.2007.11.006. Epub 2007 Nov 29.
[20] Cunningham CE, Deal K, Rimas H et al. Using conjoint analysis to model the preferences of different patient segments for attributes of patient-centered care. The Patient: Patient-Centered Outcomes Research 2008;1(4):317-30.
[21] Peay HL, Hollin I, Fischer R, Bridges JF. A community-engaged approach to quantifying caregiver preferences for the benefits and risks of emerging therapies for duchenne muscular dystrophy. Clin Ther. 2014 May 1;36(5):624-37. doi: 10.1016/j.clinthera.2014.04.011.
[22] Peay HL, Hollin I, Fischer R, Bridges JF. A community-engaged approach to quantifying caregiver preferences for the benefits and risks of emerging therapies for duchenne muscular dystrophy. Clin Ther. 2014 May 1;36(5):624-37. doi: 10.1016/j.clinthera.2014.04.011.
[23] Ciafaloni E, Fox DJ, Pandya S et al. Delayed Diagnosis in Duchenne Muscular dystrophy: Data from the Muscular dystrophy Surveillance, Tracking, and Research Network (MD STARnet). J Pediatr 2009;155:380-5.
[24]Noritz GH, Murphy NA and the Neuromotor Screening Expert Panel. Motor Delays: Early Identification and Evaluation. Pediatrics 2013;131;e2016; originally published online May 27, 2013; DOI: 10.1542/peds.2013-1056.
[25] Dent KM, Dunn DM, von Niederhausern AC et al. Improved molecular diagnosis of dystrophinopathies in an unselected clinical cohort. Am J Med Genet A. 2005;134(3): 295–298.
[26] Ciafaloni E, Fox DJ, Pandya S et al. Delayed Diagnosis in Duchenne Muscular dystrophy: Data from the Muscular dystrophy Surveillance, Tracking, and Research Network (MD STARnet). J Pediatr 2009;155:380-5.
[27] Bushby KMD, Hill A, Steele JG. Failure of early diagnosis in symptomatic Duchenne muscular dystrophy. Lancet 1999 Feb. 13 (353): 557-558.
[28] Khelifi MM, Ishmukhametova A, Khau Van Kien P et al. Pure intronic rearrangements leading to aberrant pseudoexon inclusion in dystrophinopathy: a

new class of mutations? Hum Mutat. 2011 Apr;32(4):467-75. doi:10.1002/humu.21471. Epub 2011 Mar 10.

[29] Gurvich OL, Tuohy TM, Howard MT et al. DMD pseudoexon mutations: splicing efficiency, phenotype, and potential therapy. Ann Neurol. 2008 Jan;63(1):81-9.

[30] Ishmukhametova A, Van Kien PK, Me´chin D et al. Comprehensive oligonucleotide array-comparative genomic hybridization analysis: new insights into the molecular pathology of the *DMD* gene. European Journal of Human Genetics (2012) 20, 1096–1100.

[31] Hoffman EP, Kunkel LM, Angelini C et al. Improved diagnosis of Becker muscular dystrophy by dystrophin testing. Neurology 1989;39(8):1011–7.

[32] Beggs AH, Hoffman EP, Snyder JR et al. Exploring the molecular basis for variability among patients with Becker muscular dystrophy: dystrophin gene and protein studies. Am J Hum Genet. 1991 Jul;49(1):54-67.

[33] Anthony K, Cirak S, Torelli S et al. Dystrophin quantification and clinical correlations in Becker muscular dystrophy: implications for clinical trials. Dystrophin quantification and clinical correlations in Becker muscular dystrophy: implications for clinical trials. Brain. 2011 Dec;134(Pt 12):3547-59. doi: 10.1093/brain/awr291. Epub 2011 Nov 18.

[34] Morandi L, Mora M, Tedeschi S et al. DMD and BMD in the same family due to distinct mutations. Am J Med Genet. 1995 Dec 4;59(4):501-5.,

[35] van den Bergen JC, Wokke BH, Janson AA et al. Dystrophin levels and clinical severity in Becker muscular dystrophy patients. J Neurol Neurosurg Psychiatry. 2014 Jul;85(7):747-53. doi: 10.1136/jnnp-2013-306-350.

[36] Pegoraro E, Schimke RN, Garcia C et al. Genetic and biochemical normalization in female carriers of Duchenne muscular dystrophy: evidence for failure of dystrophin production in dystrophin-competent myonuclei. Neurology. 1995 Apr;45(4):677-90.

[37] van den Bergen JC, Wokke BH, Janson AA et al. Dystrophin levels and clinical severity in Becker muscular dystrophy patients. J Neurol Neurosurg Psychiatry. 2014 Jul;85(7):747-53. doi: 10.1136/jnnp-2013-306-350.

[38] Hoffman EP, Arahata K, Minetti C et al. Dystrophinopathy in isolated cases of myopathy in females. Neurology. 1992 May;42(5):967-75.

[39] Mendell JR, Shilling C, Leslie ND et al. Evidence-based path to newborn screening for Duchenne muscular dystrophy. Ann Neurol. 2012 Mar;71(3):304-13. doi: 10.1002/ana.23528.

[40] Nicholson LV, Johnson MA, Bushby KM et al. Integrated study of 100 patients with Xp21 linked muscular dystrophy using clinical, genetic, immunochemical, and histopathological data. Part 1. Trends across the clinical groups. J Med Genet. 1993 Sep;30(9):728-36.

[41] Flanigan KM, Dunn DM, von Niederhausern A et al. Nonsense mutation-associated Becker muscular dystrophy: interplay between exon definition and splicing regulatory elements within the DMD gene. Hum Mutat. 2011 Mar;32(3):299-308. doi: 10.1002/humu.21426.

[42] Goemans N, van den Hauwe M, Wilson R et al. Ambulatory capacity and disease progression as measured by the 6-minute-walk-distance in Duchenne muscular

dystrophy subjects on daily corticosteroids. Neuromuscul Disord. 2013 Aug;23(8):618-23.

[43] McDonald CM, Henricson EK, Abresch RT et al. The 6-minute walk test and other endpoints in Duchenne muscular dystrophy: longitudinal natural history observations over 48 weeks from a multicenter study. Muscle Nerve. 2013 Sep;48(3):343-56. doi: 10.1002/mus.23902. Epub 2013 Jun 26.

[44] Bushby K, Connor E. Clinical outcome measures for trials in Duchenne muscular dystrophy: report from International Working Group meetings. Clin Investig (Lond). 2011 September; 1(9): 1217–1235. doi:10.4155/cli.11.113.

[45] Mercuri E, McDonald C, Mayhew A et al. International workshop on assessment of upper limb function in Duchenne Muscular dystrophy: Rome, 15-16 February 2012. Neuromuscul Disord. 2012 Nov;22(11):1025-8.

[46] Lynn S, Aartsma-Rus A, Furlong P et al. Measuring Clinical Effectiveness of Medicinal Products for the Treatment of Duchenne Muscular Dystrophy. In press, Neuromuscular Disorders.

[47] Bushby K, Connor E. Clinical outcome measures for trials in Duchenne muscular dystrophy: report from International Working Group meetings. Clin Investig (Lond). 2011 September; 1(9): 1217–1235. doi:10.4155/cli.11.113.

[48] McDonald CM, Henricson EK, Abresch RT et al. The cooperative international neuromuscular research group Duchenne natural history study--a longitudinal investigation in the era of glucocorticoid therapy: design of protocol and the methods used. Muscle Nerve. 2013 Jul;48(1):32-54. doi: 10.1002/mus.23807. Epub 2013 May 16.

[49] Smith RA, Sibert JR, Wallace SJ, Harper PS. Early diagnosis and secondary prevention of Duchenne muscular dystrophy. Arch Dis Child. 1989 Jun;64(6):787-90.

[50] Connolly AM, Florence JM, Cradock MM et al. One Year Outcome of Boys With Duchenne Muscular dystrophy Using the Bayley-III Scales of Infant and Toddler Development. Pediatr Neurol. 2014 Jun;50(6):557-63.

[51] Connolly AM, Florence JM, Cradock MM et al. Motor and cognitive assessment of infants and young boys with Duchenne Muscular dystrophy: results from the Muscular dystrophy Association DMD Clinical Research Network. Neuromuscul Disord. 2013 Jul;23(7):529-39.

[52] Pane M, Scalise R, Berardinelli A et al. Early neurodevelopmental assessment in Duchenne muscular dystrophy. Neuromuscul Disord. 2013 Jun;23(6):451-5. doi: 10.1016/j.nmd.2013.02.012. Epub 2013 Mar 25.

[53] Connolly AM, Florence JM, Cradock MM, Eagle M, Flanigan KM, McDonald CM, Karachunski PI, Darras BT, Bushby K, Malkus EC, Golumbek PT, Zaidman CM, Miller JP, Mendell JR; MDA DMD Clinical Research Network. One Year Outcome of Boys With Duchenne Muscular dystrophy Using the Bayley-III Scales of Infant and Toddler Development. Pediatr Neurol. 2014 Jun;50(6):557-63.

[54] Connolly AM, Florence JM, Cradock MM, Malkus EC, Schierbecker JR, Siener CA, Wulf CO, Anand P, Golumbek PT, Zaidman CM, Philip Miller J, Lowes LP, Alfano LN, Viollet-Callendret L, Flanigan KM, Mendell JR, McDonald CM, Goude E, Johnson L,

Nicorici A, Karachunski PI, Day JW, Dalton JC, Farber JM, Buser KK, Darras BT, Kang PB, Riley SO, Shriber E, Parad R, Bushby K, Eagle M; MDA DMD Clinical Research Network. Motor and cognitive assessment of infants and young boys with Duchenne Muscular dystrophy: results from the Muscular dystrophy Association DMD Clinical Research Network. Neuromuscul Disord. 2013 Jul;23(7):529-39.

[55] Passamano L, Taglia A, Palladino A et al. Improvement of survival in Duchenne Muscular Dystrophy: retrospective analysis of 835 patients ActaMyol. 2012 October; 31(2): 121-125.

[56] Bushby K, Finkel R, Birnkrant DJ et al. Diagnosis and management of Duchenne muscular dystrophy, part 1: diagnosis, and pharmacological and psychosocial management. Lancet Neurol. 2010 Jan;9(1):77-93. doi: 10.1016/S1474-4422(09)70271-6. Epub 2009 Nov 27. Review.

[57] McDonald CM, Henricson EK, Abresch RT et al. The cooperative international neuromuscular research group Duchenne natural history study--a longitudinal investigation in the era of glucocorticoid therapy: design of protocol and the methods used. Muscle Nerve. 2013 Jul;48(1):32-54. doi: 10.1002/mus.23807. Epub 2013 May 16.

[58] Schram G, Fournier A, Leduc H et al. All-cause mortality and cardiovascular outcomes with prophylactic steroid therapy in Duchenne muscular dystrophy. Journal of the American College of Cardiology Volume 61(9), 2013, 948-954.

[59] Manzur AY, Kuntzer T, Pike M, Swan A. Glucocorticoid corticosteroids for Duchenne muscular dystrophy. Cochrane Database Syst Rev. 2008 Jan 23;(1):CD003725. doi: 10.1002/14651858.CD003725.pub3.

[60] Henricson EK, Abresch RT, Cnaan A et al. The cooperative international neuromuscular research group Duchenne natural history study: glucocorticoid treatment preserves clinically meaningful functional milestones and reduces rate of disease progression as measured by manual muscle testing and other commonly used clinical trial outcome measures. Muscle Nerve. 2013 Jul;48(1):55-67. doi: 10.1002/mus.23808. Epub 2013 May 6.

[61] Bushby K, Finkel R, Birnkrant DJ et al. Diagnosis and management of Duchenne muscular dystrophy, part 1: diagnosis, and pharmacological and psychosocial management. Lancet Neurol, 9(1): 77-93, 2010.

[62] McDonald CM, Abresch RT, Carter GT et al. Profiles of neuromuscular diseases. Duchenne muscular dystrophy. Am J Phys Med Rehabil. 1995 Sep-Oct;74(5 Suppl):S70-92.

[63] Bushby K, Finkel R, Birnkrant DJ et al. Diagnosis and management of Duchenne muscular dystrophy, part 1: diagnosis, and pharmacological and psychosocial management. Lancet Neurol. 2010 Jan;9(1):77-93. doi: 10.1016/S1474-4422(09)70271-6. Epub 2009 Nov 27. Review.

[64]Skalsky AJ, McDonald CM. Prevention and management of limb contractures in neuromuscular diseases. Phys Med RehabilClin N Am. 2012 Aug;23(3):675-87.

[65] Lebel DE, Corston JA, McAdam LC et al. Glucocorticoid treatment for the prevention of scoliosis in children with Duchenne muscular dystrophy: long-term

follow-up. J Bone Joint Surg Am. 2013 Jun 19;95(12):1057-61. doi: 10.2106/JBJS.L.01577.

[66] Eagle M, Bourke J, Bullock R et al. Managing Duchenne muscular dystrophy--the additive effect of spinal surgery and home nocturnal ventilation in improving survival. Neuromuscul Disord. 2007 Jun;17(6):470-5. Epub 2007 May 8

[67] Finder JD, Birnkrant D, Carl J et al. Respiratory care of the patient with Duchenne muscular dystrophy: ATS consensus statement. Am J Respir Crit Care Med. 2004 Aug 15;170(4):456-65. Review.

[68] Passamano L, Taglia A, Palladino A et al. Improvement of survival in Duchenne Muscular Dystrophy: retrospective analysis of 835 patients ActaMyol. 2012 October; 31(2): 121-125.

[69] Rall S, Grimm T. Survival in Duchenne muscular dystrophy. Acta Myol. 2012 Oct;31(2):117-20.

[70] Duboc D, Meune C, Pierre B et al. Perindopril preventive treatment on mortality in Duchenne muscular dystrophy: 10 years' follow-up. Am Heart J. 2007 Sep;154(3):596-602.

[71] Ryan TD, Jefferies JL, Sawnani H et al. Implantation of the HeartMate II and HeartWare left ventricular assist devices in patients with duchenne muscular dystrophy: lessons learned from the first applications. ASAIO J. 2014 Mar-Apr;60(2):246-8.

[72] McDonald CM, Henricson, EK, Abresch, RT et al. The 6-minute walk test and other endpoints in nonsense-mutation-mediated dystrophinopathy: longitudinal natural history observations in a multicenter study. Muscle & Nerve. 2013 48(3):343-356

[73] Humbertclaude V, Hamroun D, Bezzou K et al. Motor and respiratory heterogeneity in Duchenne patients: implication for clinical trials. Eur J Paediatr Neurol. 2012 Mar;16(2):149-60. doi: 10.1016/j.ejpn.2011.07.001. Epub 2011 Sep 15.

[74] Henricson EK, Abresch RT, Cnaan A et al. The cooperative international neuromuscular research group Duchenne natural history study: glucocorticoid treatment preserves clinically meaningful functional milestones and reduces rate of disease progression as measured by manual muscle testing and other commonly used clinical trial outcome measures. Muscle Nerve. 2013 Jul;48(1):55-67. doi: 10.1002/mus.23808. Epub 2013 May 6.

[75] Humbertclaude V, Hamroun D, Bezzou K et al. Motor and respiratory heterogeneity in Duchenne patients: implication for clinical trials. Eur J Paediatr Neurol. 2012 Mar;16(2):149-60. doi: 10.1016/j.ejpn.2011.07.001. Epub 2011 Sep 15.

[76] Pane M, Mazzone ES, Sormani MP et al. (2014) 6 Minute Walk Test in Duchenne MD Patients with Different Mutations: 12 Month Changes. PLoS ONE 9(1): e83400. doi:10.1371/journal.pone.0083400.

[77] Pane M, Mazzone ES, Sormani MP et al. (2014) 6 Minute Walk Test in Duchenne MD Patients with Different Mutations: 12 Month Changes. PLoS ONE 9(1): e83400. doi:10.1371/journal.pone.0083400.

[78] Muntoni F, Torelli S, Ferlini A. Dystrophin and mutations: one gene, several proteins, multiple phenotypes. Lancet Neurol. 2003 Dec;2(12):731-40. Review.
[79] D'Angelo MG, Lorusso ML, Civati F et al. Neurocognitive profiles in Duchenne muscular dystrophy and gene mutation site. Pediatr Neurol. 2011 Nov;45(5):292-9. doi: 10.1016/j.pediatrneurol.2011.08.003.
[80] Flanigan KM, Ceco E, Lamar KM et al. LTBP4 genotype predicts age of ambulatory loss in Duchenne muscular dystrophy. Ann Neurol. 2013 Apr;73(4):481-8. doi: 10.1002/ana.23819. Epub 2013 Feb 20.
[81] Pegoraro E, Hoffman EP, Piva L et al. SPP1 genotype is a determinant of disease severity in Duchenne muscular dystrophy. Neurology. 2011 Jan 18;76(3):219-26. doi: 10.1212/WNL.0b013e318207afeb. Epub 2010 Dec 22.
[82] Mayhew AG, Cano SJ, Scott E et al. Detecting meaningful change using the North Star Ambulatory Assessment in Duchenne muscular dystrophy.Dev Med Child Neurol. 2013 Nov;55(11):1046-52. doi: 10.1111/dmcn.12220. Epub 2013 Aug 5.
[83] Ricotti V, Ridout DA, Scott E et al. Long-term benefits and adverse effects of intermittent versus daily glucocorticoids in boys with Duchenne muscular dystrophy. J Neurol Neurosurg Psychiatry. 2013 Jun;84(6):698-705. doi: 10.1136/jnnp-2012-303902. Epub 2012 Dec 18.
[84] Bushby K, Finkel R, Birnkrant DJ et al. Diagnosis and management of Duchenne muscular dystrophy, part 2: implementation of multidisciplinary care. Lancet Neurol. 2010 Feb;9(2):177-89. doi: 10.1016/S1474-4422(09)70272-8. Epub 2009 Nov 27. Review. Erratum in: Lancet Neurol. 2010 Mar;9(3):237.
[85] Grimm T, Kress W, Meng G, Müller CR. Risk assessment and genetic counseling in families with Duchenne muscular dystrophy. Acta Myol. 2012 Dec;31(3):179-83.
[86] Pane M, Scalise R, Berardinelli A et al. Early neurodevelopmental assessment in Duchenne muscular dystrophy.Neuromuscul Disord. 2013 Jun;23(6):451-5. doi: 10.1016/j.nmd.2013.02.012. Epub 2013 Mar 25.
[87] Connolly AM, Florence JM, Cradock MM et al. One Year Outcome of Boys With Duchenne Muscular dystrophy Using the Bayley-III Scales of Infant and Toddler Development. Pediatr Neurol. 2014 Jun;50(6):557-63.
[88] Connolly AM, Florence JM, Cradock MM et al. Motor and cognitive assessment of infants and young boys with Duchenne Muscular dystrophy: results from the Muscular dystrophy Association DMD Clinical Research Network. Neuromuscul Disord. 2013 Jul;23(7):529-39.
[89] Scott E, Mawson SJ. Measurement in Duchenne muscular dystrophy: considerations in the development of a neuromuscular assessment tool. Dev Med Child Neurol. 2006 Jun;48(6):540-4. Review.
[90] Mazzone ES, Messina S, Vasco G et al. Reliability of the North Star Ambulatory Assessment in a multicentric setting. Neuromuscul Disord. 2009 Jul;19(7):458-61. Epub 2009 Jun 23.
[91] Mazzone E, Martinelli D, Berardinelli A et al. North Star Ambulatory Assessment, 6-minute walk test and timed items in ambulant boys with Duchenne muscular dystrophy. Neuromuscul Disord 2010;20(11):712-716.

[92] Mayhew A, Cano S, Scott E et al. Moving towards meaningful measurement: Rasch analysis of the North Star Ambulatory Assessment in Duchenne muscular dystrophy. Dev Med Child Neurol 2011;53(6):535-542.
[93] Scott E, Eagle M, Mayhew A, et al. Development of a Functional Assessment Scale for Ambulatory Boys with Duchenne Muscular dystrophy. Physiother Res Int. 2011 Sep 23. doi: 10.1002/pri.520. [Epub ahead of print].
[94] Mayhew AG, Cano SJ, Scott E et al. Detecting meaningful change using the North Star Ambulatory Assessment in Duchenne muscular dystrophy. Dev Med Child Neurol. 2013 Nov;55(11):1046-52.
[95] Mayhew AG, Cano SJ, Scott E et al. Detecting meaningful change using the North Star Ambulatory Assessment in Duchenne muscular dystrophy. Dev Med Child Neurol. 2013 Nov;55(11):1046-52.
[96] Brooke MH, Fenichel GM, Griggs RC et al. Clinical investigation in Duchenne dystrophy: 2. Determination of the "power" of therapeutic trials based on the natural history. Muscle Nerve 1983;6(2):91-103.
[97] Brooke MH, Griggs RC, Mendell JR et al. Clinical trial in Duchenne dystrophy. I. The design of the protocol. Muscle Nerve 1981;4(3):186-197.
[98] Fowler WM, Jr., Abresch RT, Aitkens S et al. Profiles of neuromuscular diseases. Design of the protocol. Am J Phys Med Rehabil 1995;74(5 Suppl):S62-69.
[99] Mayhew JE, Florence JM, Mayhew TP et al. Reliable surrogate outcome measures in multicenter clinical trials of Duchenne muscular dystrophy. Muscle Nerve 2007;35(1):36-42.
[100] McDonald CM, Henricson EK, Abresch RT et al. The 6-minute walk test and other clinical endpoints in duchenne muscular dystrophy: reliability, concurrent validity, and minimal clinically important differences from a multicenter study. Muscle Nerve. 2013 Sep;48(3):357-68. doi: 10.1002/mus.23905. Epub 2013 Jul 17.
[101] Brooke MH, Fenichel GM, Griggs RC et al. Clinical investigation in Duchenne dystrophy: 2. Determination of the "power" of therapeutic trials based on the natural history. Muscle Nerve 1983;6(2):91-103.
[102] Brooke MH, Griggs RC, Mendell JR et al. Clinical trial in Duchenne dystrophy. I. The design of the protocol. Muscle Nerve 1981;4(3):186-197.
[103] Fowler WM, Jr., Abresch RT, Aitkens S et al. Profiles of neuromuscular diseases. Design of the protocol. Am J Phys Med Rehabil 1995;74(5 Suppl):S62-69.
[104] Mayhew JE, Florence JM, Mayhew TP et al. Reliable surrogate outcome measures in multicenter clinical trials of Duchenne muscular dystrophy. Muscle Nerve 2007;35(1):36-42.
[105] Mendell JR, Moxley RT, Griggs RC et al. Randomized, double-blind six-month trial of prednisone in Duchenne's muscular dystrophy. N Engl J Med. 1989 Jun 15;320(24):1592-7.
[106] McDonald CM, Henricson EK, Abresch RT et al. The 6-minute walk test and other clinical endpoints in duchenne muscular dystrophy: reliability, concurrent validity, and minimal clinically important differences from a multicenter study. Muscle Nerve. 2013 Sep;48(3):357-68. doi: 10.1002/mus.23905. Epub 2013 Jul 17.

[107] Brooke MH, Fenichel GM, Griggs RC et al. Clinical investigation in Duchenne dystrophy: 2. Determination of the "power" of therapeutic trials based on the natural history. Muscle Nerve 1983;6(2):91-103.
[108] Brooke MH, Griggs RC, Mendell JR et al. Clinical trial in Duchenne dystrophy. I. The design of the protocol. Muscle Nerve 1981;4(3):186-197.
[109] Fowler WM, Jr., Abresch RT, Aitkens S et al. Profiles of neuromuscular diseases. Design of the protocol. Am J Phys Med Rehabil 1995;74(5 Suppl):S62-69.
[110] Mendell JR, Moxley RT, Griggs RC et al. Randomized, double-blind six-month trial of prednisone in Duchenne's muscular dystrophy. N Engl J Med. 1989 Jun 15;320(24):1592-7.
[111] Mayhew JE, Florence JM, Mayhew TP et al. Reliable surrogate outcome measures in multicenter clinical trials of Duchenne muscular dystrophy. Muscle Nerve 2007;35(1):36-42.
[112] Mayhew JE, Florence JM, Mayhew TP et al. Reliable surrogate outcome measures in multicenter clinical trials of Duchenne muscular dystrophy. Muscle Nerve 2007;35(1):36-42.
[113] McDonald CM, Henricson EK, Abresch RT et al. The 6-minute walk test and other clinical endpoints in duchenne muscular dystrophy: reliability, concurrent validity, and minimal clinically important differences from a multicenter study. Muscle Nerve. 2013 Sep;48(3):357-68. doi: 10.1002/mus.23905. Epub 2013 Jul 17.
[114] Bushby K, Finkel R, Wong B et al. Ataluren Treatment of Patients with Nonsense Mutation Dystrophinopathy. In press, Muscle & Nerve.
[115] Brooke MH, Fenichel GM, Griggs RC et al. Clinical investigation in Duchenne dystrophy: 2. Determination of the "power" of therapeutic trials based on the natural history. Muscle Nerve 1983;6(2):91-103.
[116] Brooke MH, Griggs RC, Mendell JR et al. Clinical trial in Duchenne dystrophy. I. The design of the protocol. Muscle Nerve 1981;4(3):186-197.
[117] Fowler WM, Jr., Abresch RT, Aitkens S et al. Profiles of neuromuscular diseases. Design of the protocol. Am J Phys Med Rehabil 1995;74(5 Suppl):S62-69.
[118] Bushby K, Finkel R, Wong B et al. Ataluren Treatment of Patients with Nonsense Mutation Dystrophinopathy. In press, Muscle & Nerve.
[119] McDonald CM, Henricson EK, Han JJ et al.The 6-minute walk test as a new outcome measure in Duchenne muscular dystrophy. Muscle Nerve. 2010 Apr;41(4):500-10. doi: 10.1002/mus.21544.
[120] McDonald CM, Henricson EK, Han JJ et al. The 6-minute walk test in Duchenne/Becker muscular dystrophy: longitudinal observations. Muscle Nerve. 2010 Dec;42(6):966-74. doi: 10.1002/mus.21808.
[121] McDonald CM, Henricson EK, Abresch RT et al. The 6-minute walk test and other clinical endpoints in duchenne muscular dystrophy: reliability, concurrent validity, and minimal clinically important differences from a multicenter study. Muscle Nerve. 2013 Sep;48(3):357-68. doi: 10.1002/mus.23905. Epub 2013 Jul 17.
[122] McDonald CM, Henricson EK, Abresch RT et al. The 6-minute walk test and other clinical endpoints in duchenne muscular dystrophy: reliability, concurrent validity,

and minimal clinically important differences from a multicenter study. Muscle Nerve. 2013 Sep;48(3):357-68. doi: 10.1002/mus.23905. Epub 2013 Jul 17.
[123] Davidson ZE, Ryan MM, Kornberg AJ et al. Strong Correlation Between the 6-Minute Walk Test and Accelerometry Functional Outcomes in Boys With Duchenne Muscular Dystrophy. J Child Neurol. 2014 Apr 23. [Epub ahead of print]
[124] Mazzone E, Martinelli D, Berardinelli A et al. North Star Ambulatory Assessment, 6-minute walk test and timed items in ambulant boys with Duchenne muscular dystrophy. Neuromuscul Disord. 2010 Nov;20(11):712-6.
[125] Goemans N, van den Hauwe M, Wilson R et al. Ambulatory capacity and disease progression as measured by the 6-minute-walk-distance in Duchenne muscular dystrophy subjects on daily corticosteroids. Neuromuscul Disord. 2013 Aug;23(8):618-23.
[126] Henricson E, Abresch R, Han JJ et al. The 6-minute walk test and person-reported outcomes in boys with duchenne muscular dystrophy and typically developing controls: longitudinal comparisons and clinically-meaningful changes over one year. PLoS Curr. 2013 Jul 8;5. pii: ecurrents.md.9e17658b007eb79fcd6f723089f79e06. doi: 10.1371/currents.md.9e17658b007eb79fcd6f723089f79e06.
[127] Mazzone ES, Pane M, Sormani MP et al. 24 month longitudinal data in ambulant boys with Duchenne muscular dystrophy. PLoS One. 2013;8(1):e52512.
[128] McDonald CM, Henricson EK, Abresch RT et al. The 6-minute walk test and other endpoints in Duchenne muscular dystrophy: longitudinal natural history observations over 48 weeks from a multicenter study. Muscle Nerve. 2013 Sep;48(3):343-56.
[129] Ricotti V1, Ridout DA, Scott E, et al. NorthStar Clinical Network. Long-term benefits and adverse effects of intermittent versus daily glucocorticoids in boys with Duchenne muscular dystrophy. J Neurol Neurosurg Psychiatry. 2013 Jun;84(6):698-705. doi: 10.1136/jnnp-2012-303902. Epub 2012 Dec 18.
[130] Mendell, JR, Schilling C, Leslie, N et al. Evidence-Based Path to Newborn Screening for Duchenne Muscular Dystrophy. ANN NEUROL 2012;71:304–313.
[131] McDonald CM, Henricson EK, Abresch RT et al. The 6-minute walk test and other endpoints in Duchenne muscular dystrophy: longitudinal natural history observations over 48 weeks from a multicenter study. Muscle Nerve. 2013 Sep;48(3):343-56. doi: 10.1002/mus.23902. Epub 2013 Jun 26.
[132] Mercuri 2014 in press.
[133] Mayhew AG, Cano SJ, Scott E et al. Detecting meaningful change using the North Star Ambulatory Assessment in Duchenne muscular dystrophy. Dev Med Child Neurol. 2013 Nov;55(11):1046-52. doi: 10.1111/dmcn.12220. Epub 2013 Aug 5.
[134] Bushby 2014, in press.
[135] McDonald CM, Henricson EK, Abresch RT et al. The 6-minute walk test and other endpoints in Duchenne muscular dystrophy: longitudinal natural history observations over 48 weeks from a multicenter study. Muscle Nerve. 2013 Sep;48(3):343-56. doi: 10.1002/mus.23902. Epub 2013 Jun 26..

[136] McDonald CM, Henricson EK, Abresch RT et al. The 6-minute walk test and other endpoints in Duchenne muscular dystrophy: longitudinal natural history observations over 48 weeks from a multicenter study. Muscle Nerve. 2013 Sep;48(3):343-56. doi: 10.1002/mus.23902. Epub 2013 Jun 26.

[137] McDonald CM, Henricson EK, Abresch RT et al. The 6-minute walk test and other clinical endpoints in duchenne muscular dystrophy: reliability, concurrent validity, and minimal clinically important differences from a multicenter study. Muscle Nerve. 2013 Sep;48(3):357-68. doi: 10.1002/mus.23905. Epub 2013 Jul 17.

[138] Mayhew A, Mazzone ES, Eagle M et al. Development of the Performance of the Upper Limb module for Duchenne muscular dystrophy. Dev Med Child Neurol. 2013 Nov;55(11):1038-45. doi: 10.1111/dmcn.12213. Epub 2013 Aug 1.

[139] Pane M, Mazzone ES, Fanelli L et al. Reliability of the Performance of Upper Limb assessment in Duchenne muscular dystrophy. Neuromuscul Disord. 2014 Mar;24(3):201-6. doi: 10.1016/j.nmd.2013.11.014. Epub 2013 Dec 5.

[140] Kurillo G, Han JJ, Nicorici A, Bajcsy R. Tele-MFAsT: Kinect-Based Tele-Medicine Tool for Remote Motion and Function Assessment. Stud Health Technol Inform. 2014;196:215-21.

[141] McDonald CM, Henricson EK, Abresch RT et al. The cooperative international neuromuscular research group Duchenne natural history study--a longitudinal investigation in the era of glucocorticoid therapy: design of protocol and the methods used. Muscle Nerve. 2013 Jul;48(1):32-54. doi: 10.1002/mus.23807. Epub 2013 May 16.

[142] Steffensen B, Hyde S, Lyager S, Mattsson E. Validity of the EK scale: a functional assessment of non-ambulatory individuals with Duchenne muscular dystrophy or spinal muscular atrophy. (Physiother Res Int. 2001;6(3):119-34.

[143] Vuillerot C, Payan C, Girardot F et al. Responsiveness of the motor function measure in neuromuscular diseases. Arch Phys Med Rehabil. 2012 Dec;93(12):2251-6.e1. doi: 10.1016/j.apmr.2012.05.025. Epub 2012 Jun 13.

[144] Bach JR, Ishikawa Y, Kim H. Prevention of pulmonary morbidity for patients with Duchenne muscular dystrophy. Chest 1997;112:1024-8.

[145] Finder JD, Birnkrant D, Carl J et al. Respiratory care of the patient with Duchenne muscular dystrophy: ATS consensus statement. American journal of respiratory and critical care medicine 2004;170:456-65.

[146] Birnkrant DJ, Bushby KM, Amin RS et al. The respiratory management of patients with duchenne muscular dystrophy: a DMD care considerations working group specialty article. Pediatr Pulmonol 2010;45:739-48.

[147] Hor KN, Taylor MD, Al-Khalidi HR et al. Prevalence and distribution of late gadolinium enhancement in a large population of patients with Duchenne muscular dystrophy: effect of age and left ventricular systolic function. J Cardiovasc Magn Reson. 2013 Dec 21;15:107. doi: 10.1186/1532-429X-15-107.

[148] Spurney C, Shimizu R, Morgenroth LP et al. Cooperative international neuromuscular research group duchenne natural history study demonstrates insufficient diagnosis and treatment of cardiomyopathy in duchenne muscular dystrophy. Muscle Nerve. 2014 Jan 3. doi: 10.1002/mus.24163.

[149] Servais L, Deconinck N, Moraux A et al. Innovative methods to assess upper limb strength and function in non-ambulant Duchenne patients. Neuromuscul Disord. 2013 Feb;23(2):139-48. doi: 10.1016/j.nmd.2012.10.022. Epub 2012 Dec 5.
[150] McDonald CM, Henricson EK, Abresch RT et al. The 6-minute walk test and other endpoints in Duchenne muscular dystrophy: longitudinal natural history observations over 48 weeks from a multicenter study. Muscle Nerve. 2013 Sep;48(3):343-56. doi: 10.1002/mus.23902. Epub 2013 Jun 26.
[151] Bushby K, Connor E. Clinical outcome measures for trials in Duchenne muscular dystrophy: report from International Working Group meetings. Clin Investig (Lond). 2011 September; 1(9): 1217–1235. doi:10.4155/cli.11.113
[152] Wagner JA, Williams SA, Webster CJ. Biomarkers and surrogate end points for fit-for-purpose development and regulatory evaluation of new drugs. Clin Pharmacol Ther. 2007 Jan;81(1):104-7.
[153] Pane M, Mazzone ES, Sormani MP et al. (2014) 6 Minute Walk Test in Duchenne MD Patients with Different Mutations: 12 Month Changes. PLoS ONE 9(1): e83400. doi:10.1371/journal.pone.0083400.
[154] Aartsma-Rus A, Muntoni F.. Workshop Report: 194th ENMC international workshop. 3rd ENMC workshop on exon skipping: Towards clinical application of antisense-mediated exon skipping for Duchenne muscular dystrophy 8–10 December 2012, Naarden, The Netherlands. Neuromuscul Disord. 2013 Nov;23(11):934-44. doi: 10.1016/j.nmd.2013.06.369. Epub 2013 Jul 24.
[155] Anthony K, Arechavala-Gomeza V, Taylor LE et al. Dystrophin quantification: biological and translational research implications. Neurology. 2014: in press
[156] Brown KJ, Marathi R, Fiorillo AA, Ciccimaro EF et al. Accurate Quantitation of Dystrophin Protein in Human Skeletal Muscle Using Mass Spectrometry.J Bioanal Biomed. 2012 Dec 18;Suppl 7. pii: 001.
[157] Kleopa KA, Drousiotou A, Mavrikiou E et al. Naturally occurring utrophin correlates with disease severity in Duchenne muscular dystrophy.Hum Mol Genet. 2006 May 15;15(10):1623-8. Epub 2006 Apr 4.
[158] Pegoraro E, Schimke RN, Garcia C et al. Genetic and biochemical normalization in female carriers of Duchenne muscular dystrophy: evidence for failure of dystrophin production in dystrophin-competent myonuclei. Neurology. 1995 Apr;45(4):677-90.
[159] Hoffman EP, Arahata K, Minetti C, Bonilla E, Rowland LP. Dystrophinopathy in isolated cases of myopathy in females. Neurology. 1992 May;42(5):967-75.
[160] Hoffman EP, Kunkel LM, Angelini C et al. Improved diagnosis of Becker muscular dystrophy by dystrophin testing. Neurology 1989;39(8):1011–7.
[161] Arechavala-Gomeza V, Kinali M, Feng L et al. Immunohistological intensity measurements as a tool to assess sarcolemma-associated protein expression. Neuropathol Appl Neurobiol. 2010 Jun;36(4):265-74. doi: 10.1111/j.1365-2990.2009.01056.x. Epub 2009 Nov 25.
[162] Kleopa KA, Drousiotou A, Mavrikiou E et al. Naturally occurring utrophin correlates with disease severity in Duchenne muscula dystrophy. Hum Mol Genet. 2006 May 15;15(10):1623-8. Epub 2006 Apr 4.

[163] Anthony K, Feng L, Arechavala-Gomeza V et al. Exon skipping quantification by quantitative reverse-transcription polymerase chain reaction in Duchenne muscular dystrophy patients treated with the antisense oligomer eteplirsen. Human gene therapy methods. 2012;23(5):336-45.
[164] Bassi E, Falzarano S, Fabris M et al. Persistent dystrophin protein restoration 90 days after a course of intraperitoneally administered naked 2'OMePS AON and ZM2 NP-AON complexes in mdx mice. Journal of biomedicine & biotechnology. 2012;2012:897076.
[165] Ferlini A, Rimessi P. Exon skipping quantification by real-time PCR. Methods in molecular biology. 2012;867:189-99.
[166] Kinali M, Arechavala-Gomeza V, Feng L et al. Local restoration of dystrophin expression with the morpholino oligomer AVI-4658 in Duchenne muscular dystrophy: a single-blind, placebo-controlled, dose-escalation, proof-of-concept study. Lancet Neurol. 2009;8(10):918-28.
[167] Davies DR Gelinas AD, Zhang C *et al.* Unique Motifs and Hydrophobic Interactions Shape the Binding of Modified DNA Ligands to Protein Targets. *ProcNatlAcadSci USA* 2012:**109**: 19971-19976.
[168] Brody E, Gold L, Lawn RM *et al*. High-content affinity-based proteomics: unlocking protein biomarker discovery. *Exp Rev MolDiagn* 2010:**10**(8):1013-1022.
[169] Gold L, Janjic N, Jarvis T *et al.* Aptamers and the RNA World, Past and Present. *Cold Spring HarbPerspect Biol.* doi:10.1102/cshperspect.a003582 (published in advance, December 8, 2010, part of “The RNA World” series).
[170] Kraemer S, Vaught JD, Bock C *et al.* From SOMAmer-based biomarker discovery to diagnostic and clinical applications: A SOMAmer-based, streamlined multiplex proteomic assays. *PLOS ONE* 2011:**6**(10): e26332.
[171] Veidal, SS, Vassiliadis, E, Bay-Jensen, A-C,et al. Procollagen type I N-terminal propeptide (PINP) is a marker for fibrogenesis in bile duct ligation-induced fibrosis in rats. Fibrogenesis & Tissue Repair, 2010, 3(1), 5. doi:10.1186/1755-1536-3-5.
[172] Leeming, DJ, Byrjalsen, I, Jiménez, W et al. Protein fingerprinting of the extracellular matrix remodelling in a rat model of liver fibrosis--a serological evaluation. Liver International : Official Journal of the International Association for the Study of the Liver, 2013:33(3), 439–447. doi:10.1111/liv.12044
[173] Nielsen MJ, Nedergaard, AF, Sun S et al. The neo-epitope specific PRO-C3 ELISA measures true formation of type III collagen associated with liver and muscle parameters. American Journal of Translational Research, 2013:5(3), 303–315.
[174] Nedergaard et al. Journal of Sarcipenia, Cachexia and Muscle Research, 2014 in submission.
[175] Nadarajah, V D, van Putten M, Chaouch A et al. Serum matrix metalloproteinase-9 (MMP-9) as a biomarker for monitoring disease progression in Duchenne muscular dystrophy (DMD). Neuromuscular disorders : NMD. 2011;21(8):569-578. Epub 2011/07/05. doi: 10.1016/j.nmd.2011.05.011.
[176] Martin CF, Hiller M, Spitali P et al. Fibronectin is a serum biomarker for Duchenne muscular dystrophy. Proteomics. Clinical applications. 2014. Epub 2014/01/25. doi: 10.1002/prca.201300072.

[177] Nakagawa T, Takeuchi A, Kakiuchi R et al. A prostaglandin D2 metabolite is elevated in the urine of Duchenne muscular dystrophy patients and increases further from 8 years old. Clinica chimica acta; international journal of clinical chemistry. 2013;423:10-14. Epub 2013/04/23. doi: 10.1016/j.cca.2013.03.031.
[178] Ellero-Simatos S, Szymanska E, Rullmann, T et al. Assessing the metabolic effects of prednisolone in healthy volunteers using urine metabolic profiling. Genome medicine. 2012;4(11):94. Epub 2012/12/04. doi: 10.1186/gm395.
[179] Hu J, Kong M, Ye Y et al. Serum miR-206 and other muscle-specific microRNAs as non-invasive biomarkers for Duchenne muscular dystrophy. Journal of neurochemistry. 2014. Epub 2014/01/28. doi: 10.1111/jnc.12662.
[180] Zaharieva IT, Calissano M, Scoto M et al. Dystromirs as serum biomarkers for monitoring the disease severity in Duchenne muscular Dystrophy. *PloS One*. 2013;8(11):e80263. Epub 2013/11/28. doi: 10.1371/journal.pone.0080263.
[181] Cacchiarelli D, Legnini I, Martone J et al. miRNAs as serum biomarkers for Duchenne muscular dystrophy. EMBO molecular medicine. 2011;3(5):258-265. Epub 2011/03/23. doi: 10.1002/emmm.201100133. PubMed PMID: 21425469;
[182] Hoffman EP, Brown RH, Jr. & Kunkel LM. Dystrophin: the protein product of the Duchenne muscular dystrophy locus. Cell. 1987;51(6):919-928. Epub 1987/12/24.
[183] Petrof BJ, Shrager JB, Stedman HH et al. Dystrophin protects the sarcolemma from stresses developed during muscle contraction. Proc Natl Acad Sci U S A. 1993 Apr 15;90(8):3710-4.
[184] Petrof BJ. The molecular basis of activity-induced muscle injury in Duchenne muscular dystrophy. Mol Cell Biochem. 1998 Feb;179(1-2):111-23. Review.
[185] Kamogawa Y, Biro S, Maeda M et al. Dystrophin-deficient myocardium is vulnerable to pressure overload in vivo. Cardiovasc Res. 2001 Jun;50(3):509-15.
[186] Dudley RW, Danialou G, Govindaraju K et al. Sarcolemmal damage in dystrophin deficiency is modulated by synergistic interactions between mechanical and oxidative/nitrosative stresses. Am J Pathol. 2006 Apr;168(4):1276-87; quiz 1404-5.
[187] Legrand B, Giudice E, Nicolas A et al. Computational study of the human dystrophin repeats: interaction properties and molecular dynamics. PLoS One. 2011;6(8):e23819. doi: 10.1371/journal.pone.0023819. Epub 2011 Aug 25.
[188] Legrand B, Giudice E, Nicolas A et al. Computational study of the human dystrophin repeats: interaction properties and molecular dynamics. PLoS One. 2011;6(8):e23819. doi: 10.1371/journal.pone.0023819. Epub 2011 Aug 25.
[189] Hakim CH, Grange RW, Duan D. The passive mechanical properties of the extensor digitorum longus muscle are compromised in 2- to 20-mo-old mdx mice. J Appl Physiol (1985). 2011 Jun;110(6):1656-63. doi: 10.1152/japplphysiol.01425.2010. Epub 2011 Mar 17.
[190] Hughes MS, Marsh JN, Wallace KD et al. Sensitive ultrasonic detection of dystrophic skeletal muscle in patients with duchenne muscular dystrophy using an entropy-based signal receiver. Ultrasound Med Biol. 2007 Aug;33(8):1236-43. Epub 2007 Apr 30.
[191] Pillen S, Arts IM, Zwarts MJ. Muscle ultrasound in neuromuscular disorders. Muscle Nerve. 2008 Jun;37(6):679-93. doi: 10.1002/mus.21015. Review.

[192] Forst R, Casser HR. [7 MHz real-time sonography of the skeletal musculature in Duchenne muscular dystrophy]. Ultraschall Med. 1985 Dec;6(6):336-40. German.
[193] Maurits NM, Beenakker EA, van Schaik DE et al. Muscle ultrasound in children: normal values and application to neuromuscular disorders. Ultrasound Med Biol. 2004 Aug;30(8):1017-27.
[194] Jansen M, van Alfen N, Nijhuis van der Sanden MW et al. Quantitative muscle ultrasound is a promising longitudinal follow-up tool in Duchenne muscular dystrophy. Neuromuscul Disord. 2012;22(4):306-17.
[195] Rutkove SB, Darras BT. Electrical impedance myography for the assessment of children with muscular dystrophy: a preliminary study. Journal of physics. Conference series. 2013;434(1). Epub 2013/07/31. doi: 10.1088/1742-6596/434/1/012069.
[196] Skalsky, AJ, Han JJ, Abresch RT et al. Assessment of regional body composition with dual-energy X-ray absorptiometry in Duchenne muscular dystrophy: correlation of regional lean mass and quantitative strength. Muscle & nerve. 2009;39(5):647-651. Epub 2009/04/07. doi: 10.1002/mus.21212.
[197] Forbes SC, Walter GA, Rooney WD et al. Skeletal Muscles of Ambulant Children with Duchenne Muscular Dystrophy: Validation of Multicenter Study of Evaluation with MR Imaging and MR Spectroscopy. Radiology. 2013. Epub 2013/05/23. doi: 10.1148/radiol.13121948..
[198] Willcocks RJ, Arpan IA, Forbes SC et al. Longitudinal measurements of MRI-T2 in boys with Duchenne muscular dystrophy: effects of age and disease progression. Neuromuscul Disord. 2014 May;24(5):393-401. doi: 10.1016/j.nmd.2013.12.012. Epub 2014 Jan 11.
[199] Lehmann-Horn F, Weber MA, Nagel AM et al. Rationale for treating oedema in Duchenne muscular dystrophy with eplerenone. Acta Myol. 2012 May;31(1):31-9.
[200] Kornegay JN, Bogan JR, Bogan DJ et al. Canine models of Duchenne muscular dystrophy and their use in therapeutic strategies. Mamm Genome. 2012 Feb;23(1-2):85-108. doi: 10.1007/s00335-011-9382-y. Epub 2012 Jan 5. Review.
[201] Bish LT, Sleeper MM, Forbes SC et al. Long-term systemic myostatin inhibition via liver-targeted gene transfer in golden retriever muscular dystrophy. Hum Gene Ther. 2011 Dec;22(12):1499-509. doi:10.1089/hum.2011.102. Epub 2011 Aug 30.
[202] Kim HK, Laor T, Horn PS, Wong B. Quantitative assessment of the T2 relaxation time of the gluteus muscles in children with Duchenne muscular dystrophy: a comparative study before and after steroid treatment. Korean J Radiol. 2010 May-Jun;11(3):304-11. doi: 10.3348/kjr.2010.11.3.304. Epub 2010 Apr 29..
[203] Mavrogeni S, Papavasiliou A, Douskou M et al. Effect of deflazacort on cardiac and sternocleidomastoid muscles in Duchenne muscular dystrophy: a magnetic resonance imaging study. Eur J Paediatr Neurol. 2009 Jan;13(1):34-40. doi: 10.1016/j.ejpn.2008.02.006. Epub 2008 Apr 11.
[204] Walter G, Cordier L, Bloy D, Sweeney HL. Noninvasive monitoring of gene correction in dystrophic muscle. Magn Reson Med. 2005 Dec;54(6):1369-76.
[205] Torrente Y, Belicchi M, Marchesi C et al. Autologous transplantation of muscle-derived CD133+ stem cells in Duchenne muscle patients. Cell Transplant.

2007;16(6):563-77.
[206] Miller RG, Sharma KR, Pavlath GK et al. Myoblast implantation in Duchenne muscular dystrophy: the San Francisco study. Muscle Nerve. 1997 Apr;20(4):469-78.
[207] Karpati G, Ajdukovic D, Arnold D et al. Myoblast transfer in Duchenne muscular dystrophy. Ann Neurol. 1993 Jul;34(1):8-17. PubMed PMID: 8517684
[208] Wary C, Azzabou N, Zehrouni K et al. P.13.6 Nuclear Magnetic Resonance imaging and spectroscopy provide quantitative indices of disease severity in forearms of boys with Duchenne Muscle Dystrophy. Neuromuscular disorders : NMD. 2013;23(9):810.
[209] Wary C, Azzabou N, Giraudeau C et al. Quantitative NMRI and NMRS Indices Identify Augmented Disease Progression After Loss of Ambulation in Forearms of Boys with Duchenne Muscle Dystrophy. Proc. International Soc. Magn. Reson. Med. 22:4006 (2014)
[210] Liu GC, Jong YJ, Chiang CH & Jaw TS. Duchenne muscular dystrophy: MR grading system with functional correlation. Radiology. 1993;186(2):475-480.
[211] Matsumura K, Nakano I, Fukuda N et al. Proton spin-lattice relaxation time of Duchenne dystrophy skeletal muscle by magnetic resonance imaging. Muscle & nerve. 1988;11(2):97-102. Epub 1988/02/01. doi: 10.1002/mus.880110202.
[212] Huang Y, Majumdar S, Genant HK et al. Quantitative MR relaxometry study of muscle composition and function in Duchenne muscular dystrophy. J Magn Reson Imaging. 1994;4(1):59-64. Epub 1994/01/01.
[213] Marden FA, Connolly AM, Siegel MJ, Rubin DA. Compositional analysis of muscle in boys with Duchenne muscular dystrophy using MR imaging. Skeletal Radiol. 2005;34(3):140-148. Epub 2004/11/13. doi: 10.1007/s00256-004-0825-3.
[214] Papavasiliou A, Cokkinos DV. Cardiac and sternocleidomastoid muscle involvement in Duchenne muscular dystrophy: an MRI study. Chest. 2005;127(1):143-148.
[215] Kim HK, Laor T, Horn PS et al. T2 mapping in Duchenne muscular dystrophy: distribution of disease activity and correlation with clinical assessments. Radiology. 2010;255(3):899-908. Epub 2010/05/27. doi: 10.1148/radiol.10091547.
[216] Mercuri E, Jungbluth H, Muntoni F. Muscle imaging in clinical practice: diagnostic value of muscle magnetic resonance imaging in inherited neuromuscular disorders. Curr Opin Neurol. 2005;18(5):526-537.
[217] Mathur S, Lott DJ, Senesac C et al. Age-related differences in lower-limb muscle cross-sectional area and torque production in boys with Duchenne muscular dystrophy. Arch Phys Med Rehabil. 2010;91(7):1051-1058
[218] Kinali M, Arechavala-Gomeza V, Cirak S et al. Muscle histology vs MRI in Duchenne muscular dystrophy. Neurology. 2011;76(4):346-353. Epub 2011/01/26. doi: 10.1212/WNL.0b013e318208811f.
[219] Torriani M, Townsend E, Thomas BJ et al. Lower leg muscle involvement in Duchenne muscular dystrophy: an MR imaging and spectroscopy study. Skeletal Radiol. 2012;41(4):437-445.

[220] Triplett WT, Baligand C, Forbes SC et al. Chemical shift-based MRI to measure fat fractions in dystrophic skeletal muscle. Magnetic resonance in medicine : official journal of the Society of Magnetic Resonance in Medicine / Society of Magnetic Resonance in Medicine. 2013. Epub 2013/09/06. doi: 10.1002/mrm.24917.
[221] Kan HE, Scheenen TW, Wohlgemuth M et al. Quantitative MR imaging of individual muscle involvement in facioscapulohumeral muscular dystrophy. Neuromuscular disorders : NMD. 2009;19(5):357-362.
[222]Miller RG, Sharma KR, Pavlath GK et al. Myoblast implantation in Duchenne muscular dystrophy: the San Francisco study. Muscle Nerve. 1997;20(4):469-478. Epub 1997/04/01.
[223] Bish LT, Sleeper MM, Forbes SC et al. Long-term systemic myostatin inhibition via liver-targeted gene transfer in golden retriever muscular dystrophy. Hum Gene Ther. 2011;22(12):1499-1509. Epub 2011/07/27. doi: 10.1089/hum.2011.102.
[224] Misra LK, Luthra MG, Amtey SR et al. Enhanced T1 differentiation between normal and dystrophic muscles. Magnetic resonance imaging. 1984;2(1):33-35. Epub 1984/01/01.
[225]Mercuri E, Jungbluth H & Muntoni F. Muscle imaging in clinical practice: diagnostic value of muscle magnetic resonance imaging in inherited neuromuscular disorders. Curr Opin Neurol. 2005;18(5):526-537.
[226] van Deutekom JC, Janson AA, Ginjaar IB. Local dystrophin restoration with antisense oligonucleotide PRO051. The New England journal of medicine. 2007;357(26):2677-2686. Epub 2007/12/28. doi: 10.1056/NEJMoa073108.
[227] Fan Z, Wang J, Ahn M et al. Characteristics of magnetic resonance imaging biomarkers in a natural history study of golden retriever muscular dystrophy. Neuromuscular disorders : NMD. 2014;24(2):178-191. Epub 2013/12/04. doi: 10.1016/j.nmd.2013.10.005.
[228] Wang J, Fan Z, Vandenborne K et al. A computerized MRI biomarker quantification scheme for a canine model of Duchenne muscular dystrophy. International journal of computer assisted radiology and surgery. 2013;8(5):763-774. Epub 2013/01/10. doi: 10.1007/s11548-012-0810-6.
[229] Thibaud JL, Monnet A, Bertoldi D et al. Characterization of dystrophic muscle in golden retriever muscular dystrophy dogs by nuclear magnetic resonance imaging. Neuromuscular disorders : NMD. 2007;17(7):575-584. Epub 2007/06/01. doi: 10.1016/j.nmd.2007.03.013.
[230] Liu GC, Jong YJ, Chiang CH & Jaw TS. Duchenne muscular dystrophy: MR grading system with functional correlation. Radiology. 1993;186(2):475-480.
[231] Marden FA, Connolly AM, Siegel M J, Rubin DA. Compositional analysis of muscle in boys with Duchenne muscular dystrophy using MR imaging. Skeletal Radiol. 2005;34(3):140-148. Epub 2004/11/13. doi: 10.1007/s00256-004-0825-3.
[232] Kim HK, Laor T, Horn PS et al. T2 mapping in Duchenne muscular dystrophy: distribution of disease activity and correlation with clinical assessments. Radiology. 2010;255(3):899-908. Epub 2010/05/27. doi: 10.1148/radiol.10091547.

[233] Mathur S, Lott DJ, Senesac C et al. Age-related differences in lower-limb muscle cross-sectional area and torque production in boys with Duchenne muscular dystrophy. Arch Phys Med Rehabil. 2010;91(7):1051-1058.
[234] Kinali M, Arechavala-Gomeza V, Cirak S et al. Muscle histology vs MRI in Duchenne muscular dystrophy. Neurology. 2011;76(4):346-353. Epub 2011/01/26. doi: 10.1212/WNL.0b013e318208811f. PubMed PMID: 21263136;
[235] Torriani M, Townsend E, Thomas BJ et al. Lower leg muscle involvement in Duchenne muscular dystrophy: an MR imaging and spectroscopy study. Skeletal Radiol. 2012;41(4):437-445.
[236] Triplett WT, Baligand C, Forbes SC et al. Chemical shift-based MRI to measure fat fractions in dystrophic skeletal muscle. Magnetic resonance in medicine : official journal of the Society of Magnetic Resonance in Medicine / Society of Magnetic Resonance in Medicine. 2013. Epub 2013/09/06. doi: 10.1002/mrm.24917.
[237] Huang Y, Majumdar S, Genant HK et al. Quantitative MR relaxometry study of muscle composition and function in Duchenne muscular dystrophy. J Magn Reson Imaging. 1994;4(1):59-64. Epub 1994/01/01.
[238] Rooney W, Forbes SWT, Wang DJ, Vos S de et al. Proc. Intl. Soc. Magn. Reson. Med. 689 (Intl. Soc. Magn. Reson. Med, 2013).
[239] Lott DJ, Forbes SC, Mathur S et al. Assessment of intramuscular lipid and metabolites of the lower leg using magnetic resonance spectroscopy in boys with Duchenne muscular dystrophy. Neuromuscul Disord. 2014 Apr 13. pii: S0960-8966(14)00101-1. doi: 10.1016/j.nmd.2014.03.013. [Epub ahead of print]
[240] Torriani M, Townsend E, Thomas BJ et al. Lower leg muscle involvement in Duchenne muscular dystrophy: an MR imaging and spectroscopy study. Skeletal Radiol. 2012 Apr;41(4):437-45. doi: 10.1007/s00256-011-1240-1. Epub 2011 Jul 29.
[241] Wren TA, Bluml S, Tseng-Ong L, Gilsanz V. Three-point technique of fat quantification of muscle tissue as a marker of disease progression in Duchenne muscular dystrophy: preliminary study. AJR Am J Roentgenol. 2008 Jan;190(1):W8-12.
[242] Janssen BH, Voet NB, Nabuurs CI et al. Distinct disease phases in muscles of facioscapulohumeral dystrophy patients identified by MR detected fat infiltration. PLoS One. 2014 Jan 14;9(1):e85416. doi: 10.1371/journal.pone.0085416. eCollection 2014.
[243] Hiba B, Richard N, Hébert LJ et al. Quantitative assessment of skeletal muscle degeneration in patients with myotonic dystrophy type 1 using MRI. J Magn Reson Imaging. 2012 Mar;35(3):678-85. doi: 10.1002/jmri.22849. Epub 2011 Nov 8.
[244] ArpanI , Willcocks RJ, Forbes S et al. Examination of Effects of Corticosteroids on Skeletal Muscles of Boys with DMD using MRI and MRS. NEUROLOGY, in press, 2014.
[245] Marden FA, Connolly AM, Siegel MJ, Rubin DA. Compositional analysis of muscle in boys with Duchenne muscular dystrophy using MR imaging. Skel. Radiol. 2005; 34(3): 140–148.

[246] Saito K, Kobayashi D, Komatsu M et al. A sensitive assay of tumor necrosis factor alpha in sera from Duchenne muscular dystrophy patients. Clinical chemistry. 2000;46(10):1703-4. Epub 2000/10/06.
[247] Messina S, Vita GL, Aguennouz M et al. Activation of NF-kappaB pathway in Duchenne muscular dystrophy: relation to age. Acta myologica : myopathies and cardiomyopathies : official journal of the Mediterranean Society of Myology / edited by the Gaetano Conte Academy for the study of striated muscle diseases. 2011;30(1):16-23. Epub 2011/08/17.
[248] Frisullo G, Frusciante R, Nociti V et al. CD8(+) T cells in facioscapulohumeral muscular dystrophy patients with inflammatory features at muscle MRI. Journal of clinical immunology. 2011;31(2):155-66. doi: 10.1007/s10875-010-9474-6.
[249] Weber MA, Nagel AM, Jurkat-Rott K, Lehmann-Horn F. Sodium (23Na) MRI detects elevated muscular sodium concentration in Duchenne muscular dystrophy. Neurology. 2011 Dec 6;77(23):2017-24. doi: 0.1212/WNL.0b013e31823b9c78. Epub 2011 Nov 23.
[250] Mavrogeni S, Papavasiliou A, Douskou M et al. Effect of deflazacort on cardiac and sternocleidomastoid muscles in Duchenne muscular dystrophy: a magnetic resonance imaging study. Eur J Paediatr Neurol. 2009;13(1):34-40. Epub 2008/04/15. doi: S1090-3798(08)00033-0 [pii] 10.1016/j.ejpn.2008.02.006.
[251] Mavrogeni S, Tzelepis GE, Athanasopoulos G et al. Cardiac and sternocleidomastoid muscle involvement in Duchenne muscular dystrophy: an MRI study. Chest. 2005;127(1):143-8. Epub 2005/01/18. doi: 127/1/143 [pii] 10.1378/chest.127.1.143.
[252] Wansapura JP, Hor KN, Mazur W et al. Left ventricular T2 distribution in Duchenne muscular dystrophy. J Cardiovasc Magn Reson. 2010;12:14. Epub 2010/03/20. doi: 10.1186/1532-429X-12-14. PubMed PMID: 20298602;
[253] Bun SS, Kober F, Jacquier A et al. Value of in vivo T2 measurement for myocardial fibrosis assessment in diabetic mice at 11.75 T. Invest Radiol. 2012;47(5):319-23. Epub 2012/04/11. doi: 10.1097/RLI.0b013e318243e062.
[254] Loganathan R, Bilgen M, Al-Hafez B, Smirnova IV. Characterization of alterations in diabetic myocardial tissue using high resolution MRI. Int J Cardiovasc Imaging. 2006;22(1):81-90. Epub 2005/12/20. doi: 10.1007/s10554-005-5386-6.
[255] Huang Y, Majumdar S, Genant HK et al. Quantitative MR relaxometry study of muscle composition and function in Duchenne muscular dystrophy. J Magn Reson Imaging. 1994;4(1):59-64.
[256] Marden FA, Connolly AM, Siegel MJ, Rubin DA. Compositional analysis of muscle in boys with Duchenne muscular dystrophy using MR imaging. Skeletal radiology. 2005;34(3):140-8. doi: 10.1007/s00256-004-0825-3.
[257] Lodi R, Muntoni F, Taylor J et al. Correlative MR imaging and 31P-MR spectroscopy study in sarcoglycan deficient limb girdle muscular dystrophy. Neuromuscular disorders : NMD. 1997;7(8):505-11. PubMed PMID: 9447608.
[258] Kesper K, Kornblum C, Reimann J et al. Pattern of skeletal muscle involvement in primary dysferlinopathies: a whole-body 3.0-T magnetic resonance imaging study.

Acta neurologica Scandinavica. 2009;120(2):111-8. doi: 10.1111/j.1600-0404.2008.01129.x.

[259] Kan HE, Scheenen TW, Wohlgemuth M et al. Quantitative MR imaging of individual muscle involvement in facioscapulohumeral muscular dystrophy. Neuromuscular disorders : NMD. 2009;19(5):357-62. doi: 10.1016/j.nmd.2009.02.009.

[260] Matsumura K, Nakano I, Fukuda N et al. Duchenne muscular dystrophy carriers. Proton spin-lattice relaxation times of skeletal muscles on magnetic resonance imaging. Neuroradiology. 1989;31(5):373-6.
