# Supplementary material for: How a patient advocacy group developed the first proposed draft guidance document for industry for submission to the U.S. Food and Drug Administration
Source: Orphanet J Rare Dis. 2015 Jun 24;10:82. doi: 10.1186/s13023-015-0281-2 (PMC4486430; doi:10.1186/s13023-015-0281-2)
Supplement: Additional file 4: — Duchenne Community Imperatives and Cover Letter. Duchenne Community Imperatives and Cover Letter submitted with draft guidance to the FDA. [file 13023_2015_281_MOESM4_ESM.pdf]

June 25, 2014

Guidance Document Submission
Division of Dockets Management (HFA-305)
5630 Fishers Lane, Rm. 1061
Rockville, MD 20852

Dr. Janet Woodcock
Center for Drug Evaluation and Research
Food and Drug Administration
10903 New Hampshire Ave
Silver Spring, MD 20993-0002

Dear Dr. Janet Woodcock and colleagues at the FDA,

This correspondence constitutes a formal submission of a draft guidance authored by a consortium of stakeholders, under the coordination of Parent Project Muscular Dystrophy (PPMD), for consideration by the Food and Drug Administration (FDA). This material is intended as a submission to the dockets as provided under the advice from the FDA's good guidance practice work group, with the expectation that FDA will seriously consider adoption of all or significant sections of this submission.

When FDA, PPMD and other interested parties met on December 12, 2013 in the spirit of public-private partnership to convene a Duchenne policy forum, we discussed the challenges designing and implementing clinical trials for rare diseases like Duchenne muscular dystrophy and the need to develop guidance to help accelerate development and the review of potential therapies for Duchenne muscular dystrophy (Duchenne). The forum concluded with an agreement that the Duchenne community, led by PPMD, would develop the first draft guidance on Duchenne for industry.

After an intensive five month long process, overseen by a steering committee, developed by working groups composed of clinical experts, developers and patients, and further reviewed by a community advisory board, we are pleased to hereby present to you the Duchenne muscular dystrophy community's draft of the Guidance for Industry: Duchenne Muscular Dystrophy: Developing Drugs for Treatment over the Spectrum of Disease, the first-ever patient advocacy-initiated draft guidance for a rare disease, written to help accelerate the development and review of potential therapies for Duchenne muscular dystrophy (Duchenne).

Our submission is prefaced by the Duchenne Imperatives, which begins with a few case studies, summarizes the document's key points, and explains the Duchenne community's key imperatives — what we hope will be the take home messages from the community for the sponsors, the academic community and for the FDA, and to serve to frame the importance of the development of guidance for the community. We understand that the FDA may choose not to formally adopt this preface, though it is hoped that such information will inform FDA's deliberations regarding adoption of the formal draft guidance, which follows.

Parent Project
Muscular Dystrophy
401 Hackensack Avenue, 9th Floor, Hackensack, New Jersey 07601
t 800.714.5437 • 201.944.9985 f 201.944.9987 e info@ParentProjectMD.org • ParentProjectMD.org

By working closely with the FDA to provide industry and other clinical trial sponsors with clearer guidance from the patient perspective, we hope to increase the likelihood that clinical trials will be designed to better match the unique needs of Duchenne patients. It is our profound hope that this, in turn, will lead to the approval of much needed treatments for all people living with Duchenne muscular dystrophy.

We look forward to engaging with you on planning a meeting in the near future to discuss the document, and welcome any questions or comments which you have regarding the material appended.

With this letter, we ask that you officially open a docket for submission of the guidance. Thank you in advance for your attention to, and consideration of, this important initiative.

Sincerely,

**Pat Furlong**
President, Parent Project Muscular Dystrophy
Steering Committee Chair

*Draft Guidance Steering Committee*

**Neera Gulati, MD**
Patient Representative

**John Bridges, PhD**
John's Hopkins University

**Kevin Flanigan, MD**
Nationwide Children's Hospital

**Craig McDonald, MD**
UC-Davis

**Justin Fallon, PhD**
Brown University

**Lee Sweeney, PhD**
University of Pennsylvania

**Lawrence Charnas, MD, PhD**
Shire

**Tim Franson, MD**
FaegreBD Consulting

**The Duchenne Community Imperatives for the
Guidance for Industry on Duchenne Muscular Dystrophy: Developing Drugs for
Treatment over the Spectrum of Disease**

Duchenne muscular dystrophy is a progressive debilitating genetic disorder characterized by the loss and degeneration of skeletal muscle, primarily in boys. It is invariably fatal. But those words fail to adequately describe the disease or its impact on the family. Each family, each parent of a child with Duchenne Muscular Dystrophy has a different story to tell about their child's lifetime of progressive loss of function, loss of independence and dependence on family and the extraordinary burden — physically, financially, emotionally and spiritually — that Duchenne places upon the caregiver and family.

***Some of our stories:***

*"My son Christopher was diagnosed with Duchenne Muscular Dystrophy in 2003 when he was two years old. Most boys continue to lose ambulation around age 12 and die in their early 20s. My son will be 13 in July 2014 and he lost what little ambulation he had been able to hang on to about a month ago when he fell and broke his leg. It has been a devastating transition for our family, even though we are trying to hide the pain and focus on all of the new freedoms that he has in his power wheelchair. In truth, I hate the power wheelchair and everything that it represents… Permanent seating brings on a host of health complications, like contractures and scoliosis and weight gain and increased heart rate and decreased pulmonary function. He is 12 years old. He should be running and growing and instead he is changing seating positions and using the standing function on his power chair. He is lifted onto the toilet and unable to get into his best friend's house because they have steps…"*

*"My son, Elijah, is 9 years old. His favorite thing to do is help me in the kitchen. He particularly enjoys baking. Other things he likes to do include dancing, drawing, and crafts. When he was four years old, he was diagnosed with DMD. As the diagnosis was not new to me; I was devastated. I knew that it was a slow death sentence. I have a family history and watched my brother, David, slowly lose muscle strength. This began with the legs, then progressed to all the other muscles in the body, his body deteriorated before my very eyes, and then finally his heart muscle failed at the age of 18. I know that without a viable therapy, I will slowly lose my son to this devastating diagnosis. As the disease progresses, he will no longer be able to complete basic daily living skills, much less do his favorite activities…"*

*"My beautiful boy had trouble walking, getting off the floor. He never crawled. Doctors assured me, 'He'll grow out of it.' My son's physical abilities peaked at 7, but he frequently fell even at a young age. His body could never keep up with his dreams and desires. It was with bittersweet relief when he broke his ankle at 10 and stopped walking. That's right. I wanted my boy to use a wheelchair. NO MORE FALLING. Whenever I wish for life without a wheelchair, I simply look back to when my child would spontaneously crumple to the ground, unable to catch himself and slam his face onto the pavement, carpet, or his own feet. I'm constantly aware that his heart may give in; he may not recover from a simple flu. The likelihood of dying young stands in stark contrast to my son's lust for life and adventure. Dying happens to us all, there's no*


401 Hackensack Avenue, 9th Floor, Hackensack, New Jersey 07601
**t** 800.714.5437 • 201.944.9985 **f** 201.944.9987 **e** info@ParentProjectMD.org • ParentProjectMD.org

*denying that. But within that reality, if given a choice for my son? It would not be drowning in his own fluids. It would not be from his heart giving out as a young man…"*

*"Every single day your son loses a muscle fiber, or two or three or ten or a thousand. And when a sufficient number are lost, it results in functional loss. Every functional loss is like a little death… and it negatively impacts and ripples through the family. This is a progressive debilitating disease. We really need hope and we need better outcomes."*

*"I want the FDA to know that as a mother with a child for Duchenne, we are desperate for any treatment that can help our son to be stronger or live longer."*

Families affected by Duchenne often feel as if the FDA is an untouchable and unreachable group of professionals tasked with making critical decisions on potential drugs. Consequently, the community has been advocating that the FDA be more flexible in its review of rare and progressive diseases like Duchenne and to decrease the time and cost of conducting those trials for companies engaged in or considering trials for potential therapies for Duchenne.

***A precedent setting collaboration between a rare disease community and the FDA***
But last year, the FDA took the unusual step of inviting the Duchenne community to write the attached draft guidance for industry, which. it is hoped, will facilitate the development of therapies to meet the unmet medical needs of boys and young men across the spectrum of disease.

Given some of the recent delays in the progress of treatments for Duchenne to market, the community had felt there was a need for greater clarity about how to develop new compounds for Duchenne — given the rarity of the disorder (particularly the rarity of some of the specific mutations leading to Duchenne that potential therapies may target), the challenges of small study populations, and an incomplete understanding of how physical changes and changes in biomarkers relate to measurable changes in strength and physical function.

The FDA typically writes its own guidance for industry which serves the purpose of providing sponsors of clinical development programs with clear information about the type of endpoints that clinical trials should measure at different stages of the disease, how a product might qualify for a breakthrough therapy designation and potentially accelerated approval, and what type of data the FDA will find convincing when reviewing a new drug application.

However, in rare diseases, it is fitting that the FDA defer to the experts in the field — in this case, the Duchenne community, and the academic and industry researchers most familiar with Duchenne Muscular Dystrophy.

The FDA has told the community that they are committed to partnering with us in this effort. And indeed, the FDA collaborated with Parent Project Muscular Dystrophy (PPMD)

and its scientific advisors to structure a forum held in December 2013 that helped launch the process of drafting this policy guidance.

After the forum, a Steering Committee was formed in order to ensure the outcomes of the policy forum result in draft guidance worthy of submission to the FDA. The Steering Committee provided overall strategic direction to the guidance development from research to publication and launch. Committee members collectively set the tone and direction of the draft guidance, ensuring that all perspectives from across the Duchenne community are represented.

Seven working groups were then established, made up of over 80 representatives of each part of the Duchenne community (parents and patients, academics, industry) responsible for contributing to the writing, assembling and reviewing each topical section for the guidance document.

These working groups covered:

1) Benefit/Risk Assessments
2) Diagnosis
3) Natural History
4) Clinical Trial Designs, Outcome Measures and Considerations
5) Muscle Biopsy Based Biomarkers
6) Non-Muscle Based Biomarkers
7) The Duchenne Imperatives (which has been responsible for this cover letter, and for making sure the community's key recommendations were included in the guidance.)

Parents and/or individuals with the diagnosis of Duchenne muscular dystrophy have been included in the Steering Committee and every working group. In addition, a Community Advisory Board was developed to allow additional participation of parents and individuals with Duchenne.

Each working group was responsible for the content of its own chapter, and as such, there may be some variance in tone and perspective. It should be noted that although the Duchenne Community has written this guidance draft, it has been written from the perspective of the FDA speaking to clinical trial sponsors. Whenever the guidance speaks in the FDA voice, we believe that those positions are supported by comments made previously by FDA and the approach shown in other guidances for in serious life-threatening (such as HIV) and orphan diseases.

This guidance is evidence based. The chapter content is referenced to papers that are either in the peer-reviewed literature, or in press. In some cases, important papers that have been submitted and that are in an advanced stage of the peer review process are cited because we expect them to be in press early in the course of the FDA review. These are highlighted in case they are not published by the time FDA completes its revised version of the draft guidance that we have submitted.

Parent Project
Muscular Dystrophy
401 Hackensack Avenue, 9th Floor, Hackensack, New Jersey 07601
t 800.714.5437 • 201.944.9985 f 201.944.9987 e info@ParentProjectMD.org • ParentProjectMD.org

We trust that the guidance can accelerate the approval of new therapies for Duchenne — not just for one drug, but to help facilitate what we hope will be a whole line of drugs that are effective for DMD that we can use in the future and a pipeline that can really change the course for this disease. This is truly precedent setting, and we are hopeful that what has been learned from this process can applied broadly to other forms of muscular dystrophy, and for rare diseases in general.

***What is in the guidance***

**Benefit risk assessments:**
In a step that is something of a departure for draft guidance to sponsors, the Steering Committee decided to launch the draft guidance with a chapter on benefit risk assessments (and patient and caregiver preferences in Duchenne) because it was felt that sponsors should begin engaging with the community from the very start of a drug development process in order to understand their current unmet medical needs and preferences.

The benefit risk framework is fundamental to FDA regulatory decision-making process. It requires evidence both on the benefits and risks of a treatment and the capacity to have risk mitigation strategies. It also requires a subjective assessment of: What is meaningful benefit, what is risk tolerance and what is an acceptable risk benefit trade-off.

The guidance describes survey mechanisms for a patient, caregiver, and a broader community approach to help fill that evidence gap on those three capacities. These survey methods also have potential roles elsewhere in the drug development process such as in the prioritization of outcomes, the understanding of needs or concerns of these communities.

It is recommended that sponsor should engage patient and community centered research in preparing submissions both to inform the FDA's benefit risk analysis but also to engage a broader constituency in preparing submissions. We hope to express the FDA's understanding of the complexity of DMD — that meaningful benefit risk tolerance and acceptable trade-offs may vary across clinical subtypes, across disease progression status, or as a consequence of preference heterogeneity across patients, parents and caregivers.

Finally, the chapter reports on the result of research already conducted by PPMD, which found that parents are willing to accept significant risks for a treatment that even merely slows progression of Duchenne. These preferences may change however, as effective treatments become available, so sponsors should always partner with patients, caregivers and organizations to understand their current preferences.

**Diagnosis**
This chapter describes the diagnosis of DMD, referring sponsors to guidance produced by the American Academy of Pediatrics on the diagnostic algorithm to be followed in boys with delays in reaching developmental milestones and motor difficulties. It then describes the appropriate laboratory investigations, including methodology, if serum creatine


401 Hackensack Avenue, 9th Floor, Hackensack, New Jersey 07601
t 800.714.5437 • 201.944.9985 f 201.944.9987 e info@ParentProjectMD.org • ParentProjectMD.org

phosphokinase (CK) activity is elevated: to either confirm the diagnosis with molecular analysis of the *DMD* gene or by assessment of dystrophin protein expression on muscle biopsy.

The chapter informs sponsors of the diagnostic delay in Duchenne — the fact that it can take parents years to get a diagnosis for their child. This reduces the number of young patients with Duchenne who could be enrolled in clinical trials at a stage of the disease when they might be most likely to benefit. The implementation of routine newborn screening (which is feasible) would resolve this issue however.

The chapter discusses the value of a genetic analysis using modern molecular diagnostic methods that are more thorough than technologies early in use, and recommends that patients who have been screened with older technologies may need to be re-tested in order to more accurately characterize their mutations.

Sponsors are also alerted to the fact that some patients may have challenges accessing genetic testing. It refers sponsors to partnerships between advocacy and industry that can facilitate access to genetic testing such as the Decode Duchenne program (https://www.duchenneconnect.org/).

Finally, the chapter discusses how genetic and protein analysis, along with clinical assessment are needed to characterize the spectrum and classifications of DMD, concluding with a brief discussion of the use of dystrophin quantitation as a prognostic biomarker.

**Natural History**

The natural history chapter is longer than in most guidances, because the community felt that it was important to more accurately characterize the clinical course of Duchenne Muscular Dystrophy — and the sources of heterogeneity in outcome — based upon the most recent evidence, with current medical management that has altered the timing of the loss of certain milestones, but has done little to change the progressive debilitating nature of the disease.

There is broad scientific consensus regarding the utility of a number of tools, instruments and outcome measures in Duchenne. These are described briefly in a schematic according to the different age groups and disease stages in which they are used. The natural history of Duchenne is then described according to disease status and age range, along with the key features of the disease stage. Sponsors are informed that Duchenne also affects cardiac and pulmonary function although the natural history of cardiomyopathy in Duchenne is somewhat unpredictable and may not be correlated with the course of limb weakness due to skeletal myopathy. This chapter then provides a description of the loss of clinical milestones that are a hallmark of disease progression in DMD.

Optimal medical management has changed the disease somewhat, for some patients who are able to access it. The key interventions and their reported effects are described.

However, it is important to note that despite these interventions, many boys still go off their feet at a young age, and the cardiomyopathy and pulmonary involvement in DMD still leads to substantially shortened lifespans.

Other key sources of heterogeneity that sponsors need to be aware of are described, including disease severity/stage of disease, genetic predictors of disease progression (in the DMD gene), non-DMD gene genetic modifiers, corticosteroid therapy, and night splinting, physical therapy, and other standard interventions.

Finally, the chapter refers sponsors to FDA guidance about the standards that should be adhered to establish adequate, reliable and well-matched natural history controls that account for known causes in variability of the disease.

***Clinical Trial Designs, Outcome Measures and Considerations***
This chapter is a response to the Duchenne Community Imperative to make sure that trials — to the degree possible — are inclusive of people with Duchenne of all ages and disease stages. Consequently, the chapter begins with some recommendations to sponsors about how to go about maximizing inclusion of Duchenne populations in studies.

The chapter builds upon what was discussed in the natural history chapter — because it is the natural history of the disease that must inform clinical trial design. Accordingly, a detailed discussion of the established clinical outcome measures follows, organized by age/disease status in motor function outcomes, focusing on aspects of each measure that could be important for a sponsor designing clinical trials. There is a discussion of some of the uncertainties with some pulmonary endpoints, as well as problems with risk prediction of cardiac endpoints — although, the heart, in particular, cannot be neglected by sponsors in clinical development programs.

Sponsors are urged to use and help the community validate existing patient reported outcome (PRO) measures or develop new PRO measures in Duchenne, according to published FDA guidance. Sponsors are also encouraged to use exploratory outcome measures and to support the development of novel outcome measures that may expand their ability to show clinical benefit across populations.

With the available tools, sponsors are encouraged to carefully consider how best to be inclusive of individuals across the spectrum of disease. They might consider small studies in different disease stages, or one key registrational study following a primary endpoint in one medically addressable population, while studying safety in other populations. However there is a danger that one key registrational study may fail to show a clear outcome, if the wrong population was chosen, or if the study didn't adequately account for heterogeneity.

Finally, the chapter discusses other considerations, such as the potential benefit to the field of sponsors working together to standardize measurements across trials, when possible, the use of biomarkers as primary and secondary endpoints in trials, and when it might be possible to extrapolate that clinical benefits demonstrated in one disease stage population might also be expected to be seen in other disease stages.

***Biomarkers***

The last chapter discusses biomarkers, beginning with a description of how the FDA views different types of biomarkers, such as predictive, pharmacodynamic or surrogate endpoint biomarkers. The chapter is then divided into the following two sections.

- Muscle Biopsy Based Biomarkers

This section addresses muscle biopsy based biomarkers including dystrophin analysis, utrophin analysis and RT/RNA PCR analysis for exon-skipping detection.

It begins with a discussion of some of the key considerations related to performing muscle biopsies — including ethical considerations that should compel the sponsor to only perform muscle biopsies when it is appropriate and there are no suitable alternatives available for their clinical development program — and to take precautions to make certain that the specimen is of adequate quality to be useful. Some aspects of biopsy handling and ways to minimize variability and sampling errors are then discussed.

The established methodologies of dystrophin analysis — immunohistochemistry and western blot are then reviewed, and sponsors are referred to an international effort to standardize these methodologies across laboratories. An emerging technology utilizing mass spectrometry, which has some potential advantages of high reliability, accuracy and sensitivity is also introduced.

There are some limitations to each of the methods. However, we still believe that widely established dystrophin analysis techniques that are used for diagnostic and prognostic purposes, can also be used as a biochemical outcome measure in clinical trials. We believe that there is a strong likelihood that a change in dystrophin will eventually lead to clinical benefit in the appropriate medically addressable population — though the population and endpoints most likely to respond to treatment, as well as the timing and degree of benefit may remain unclear at this time.

Similarly, RT or qPCR for exon-skipping detection could be used to demonstrate that antisense oligonucleotides have achieved exon-skipping, even if the method is not quantitative as of yet.

The measurement of utrophin is also addressed — including the differences between utrophin quantitation because of its biology as compared to dystrophin, the importance of the associated proteins and being able to assess the integrity of the DG complex in the muscle.

- Non-Muscle Based Biomarkers

This subsection provides short descriptions that reflect the literature of the different serum, urine and imaging biomarkers that have been used in DMD.

From the patient's standpoint, the ability to bring a non-invasive imaging technique to eventually replace biopsy, and reach the goal of being a surrogate endpoint that could get


401 Hackensack Avenue, 9th Floor, Hackensack, New Jersey 07601
**t** 800.714.5437 • 201.944.9985 **f** 201.944.9987 **e** info@ParentProjectMD.org • ParentProjectMD.org

early approval with follow-on functional data, is a very positive development.
The subsection is mostly focused on MRI/MRS imaging of skeletal muscle. We believe that the MRI/MRS has the potential to eventually be authorized as a surrogate endpoint, if it is used in treatment trials.

## Key Duchenne Community Imperatives

In the process of considering this draft guidance, the Duchenne Community Imperatives working group has developed a number of key advocacy positions. These include some statements that are not included within the guidance itself, because they are essentially messages from the community to the FDA, the sponsors or the field in general.

*Overview:*
There are critical unmet medical needs across the entire spectrum of the disease — at all ages, stages and in each sub-population of Duchenne Muscular Dystrophy — that must be addressed with greater creativity and coordination on the part of regulators, sponsors, and government-funded researchers with more meaningful engagement of patients and caregivers. The standard approach to drug development used in more common diseases has to date failed to deliver even modest therapeutic options for patients with Duchenne — or many other rare diseases for that matter.

The system isn't working for our boys and young men.

***Benefit/Risk Assessments and patient preferences***

- The FDA is a government service and as such is mandated to be responsive to the needs and requirements of the community. Well-designed scientific benefit risk assessments in the disease-specific community are required to inform the FDA's regulatory decision making — and also to inform the conduct of clinical trials.

- The community wants safe and effective treatments — however, in the absence of any FDA-approved treatment for Duchenne at all, and the recognition that each day a person with Duchenne goes without treatment brings them a step closer to losing essential functions and to death — the community has expressed a willingness to accept a certain degree of uncertainty regarding both benefit and risk. Some in the community may be willing to take even greater risk — on account of accelerated rates of progression, or their proximity to loss of a vital function or death.

- We would encourage the FDA to be responsive to changing preferences, recognizing these may be lifelong therapies for patients and that the benefit/risk assessments may differ over time.

- Note, however, this does not mean flexibility with regard to whether a trial's findings are statistically significant. Rather, the flexibility we are seeking may concern where the line is drawn as to whether an intermediate clinical endpoint is clinically meaningful,


401 Hackensack Avenue, 9th Floor, Hackensack, New Jersey 07601
**t** 800.714.5437 • 201.944.9985 **f** 201.944.9987 **e** info@ParentProjectMD.org • ParentProjectMD.org

whether post-hoc analyses can support an NDA or whether a less than precise biomarker is reasonably likely to produce clinical benefit.

- We would like to remind the Agency that accelerated approval was pioneered in HIV disease, with several drugs were granted approval on the basis of small, transient increases in CD4 cell counts — without any evidence whether those CD4 cells were new or recirculating from the gut or lymph, whether they were naïve or memory CD4 cells, or whether they would have any effect whatsoever on reducing the morbidity or mortality of the disease. In the end, the CD4 cell changes probably did signify a drug effect, but they were not the optimal surrogate endpoint in the disease. We would ask that the Agency grant us the same consideration as it did the HIV community.

***Diagnosis***

- The diagnostic delay must be decreased as new treatments become available, and guidance on the diagnostic algorithm in children with development delay must be more broadly disseminated to primary care physicians.

- However, it should be noted that newborn screening completely bypasses the clinician's judgment, and makes diagnosis a more routine process. It would have a huge impact on early diagnosis, and early intervention, and its implementation would benefit the planning of future trials.

- The community recognizes that treating earlier is likely to have a greater impact, and since newborn screening is feasible, we should be doing it.

- Likewise, access to modern methods of DMD gene analysis must be increased. The information that a complete analysis provides may be crucial for classification of disease, and for patients to qualify for future clinical trials. Issues surrounding reimbursement must be resolved.

***Natural history***

- There is a need for open access to natural history databases for aggregation and meta-analyses, in order to better define effect sizes of potential sources of heterogeneity in outcome, and better characterize the natural history of the disease.

***Clinical trial, outcome measures and considerations***

- The Duchenne Community want trials that are inclusive of people with Duchenne of all ages across the spectrum of disease — to whatever degree possible. It is up to the sponsor to determine how best to do this, whilst bringing its product to market efficiently. This could help assure a broad label for products if they receive approval.

- There is widespread support in the community to move away from placebo-controls or to use trial designs that minimize exposure to placebo. It is hard for patients and


401 Hackensack Avenue, 9th Floor, Hackensack, New Jersey 07601
t 800.714.5437 • 201.944.9985 f 201.944.9987 e info@ParentProjectMD.org • ParentProjectMD.org

families to accept being placed on placebo, knowing that every day, a child with DMD loses muscle cells and functions. We request that the FDA be flexible in this regard.

- Sponsors are requested to address the co-morbid conditions of Duchenne, such as heart disease, which may become exacerbated if new therapies do help prolong life and function.

- While post-hoc analyses are generally not of accepted by the FDA as supporting an NDA, we would ask the FDA to use flexibility in the case of rare diseases. Historically, there has been a real distinction between consideration of pre-specified analyses versus post-hoc statistical analyses. But it is inherent in any orphan or ultra orphan disease, including Duchenne, that when there is a limited knowledge regarding natural history and there are also novel endpoints that are going to emerge (particularly in the non-ambulant disease of Duchenne), that one has to view the data and the evidence moving forward through that lens. The limitation of natural history, and development of novel endpoints will naturally lead to post-hoc statistical analyses based upon emerging concepts of natural history. This is a moving target. Regulatory agencies should exhibit some flexibility with regard to post-hoc analyses, otherwise, the field will be littered with 'failed' drugs, which have not actually failed, but were merely tested in the wrong population, or using the wrong endpoints, because of the limited knowledge about the disease.

- Access to individual data: Companies should pre-specify whether or not a family will be given access to their individual data.

- Compassionate use, extension studies and informed consent:
  We recognize that sponsor's ability to offer continued treatment after a study has concluded may be limited by the lack of adequate preclinical safety data — or clear safety and efficacy concerns about the compound. However, it may be in a sponsor's best interest to consider an extension phase, given that researchers are still learning how to measure benefit in these populations — and a longer course of study may be needed to tease out how the drug might be efficacious and in which population.

  Regardless, the sponsor has an obligation to clearly explain its intentions to the community and the community has an obligation to understand what are the limitations of the sponsor's ability to offer an extension phase of a trial.

- The informed consent should specifically spell out:
  - Whether the company has a policy on pre-approval compassionate use (and/or plans for expanded access programs), and
  - What that policy is; and
  - What the expectation is about expanding into an extension phase of the study after a trial is done and if there are positive results.


401 Hackensack Avenue, 9th Floor, Hackensack, New Jersey 07601
t 800.714.5437 • 201.944.9985 f 201.944.9987 e info@ParentProjectMD.org • ParentProjectMD.org

- Also, regarding compassionate use, when a family has more than one child with DMD, and one child is included in the study, we would urge sponsors to make a plan to offer the treatment as well to the other child who may not fit the study's inclusion criteria on a compassionate use basis, as soon as safety has been established. Having one child able to access an experimental treatment, and the other not, can be a source of great distress in a family.

- Sponsors should also be aware that participation in clinical trials can be a burden for the patient and his family — particularly in older non-ambulatory boys with limited mobility. Making trials more patient- and family friendly — with appointments on weekends or after-work hours and other services to help working parents — could increase accrual in studies.

- Conducting a trial, or parts of a trial closer to home to minimize burden on family could also encourage enrollment. While there may be limitations as to where children can be dosed and where standardized outcomes can be assessed, one approach has been to have safety assessments done locally while outcome assessments are performed at specialized centers.

***Biomarkers***

- Sponsors are strongly encouraged to monitor more than one biomarker as secondary endpoints. While we believe methods of dystrophin quantitation and MRI/MRS imaging are already well established, we would ask industry to explore other non-invasive biomarkers as well to increase the likelihood that these could be used as surrogate endpoint biomarkers at some later date.

- While we acknowledge that there is *much more to learn* about dystrophin quantitation, for now, the community feels that current methods can show that a treatment is restoring dystrophin to a certain extent and we believe that it is reasonably likely to confer clinical benefit to a medically addressable population, though it remains unclear how much benefit or at what time point that clinical benefit will be seen., We feel that such evidence should be used in the accelerated approval process. The community is unwilling to wait for years for new technologies to be validated and disseminated — and we fear that if the FDA does not respect our position in this matter, it will only strengthen the hand of those who want to work outside the system and to pass legislation that undermines the FDA.

- We also believe that since MRI/MRS outcomes faithfully report on both the health and amount of skeletal muscle, they can potentially be used at different stages of the clinical trial process not only as prognostic, predictive, or pharmacodynamics biomarkers but as an efficacy-response biomarkers and surrogate endpoints to accelerate clinical development — if used in treatment trials.

**Conclusion**

- Regarding the drugs currently in the pipeline: it should be acknowledged that there has been a rather steep learning curve testing drugs in Duchenne muscular dystrophy. It would be natural for some missteps to be made by some of the sponsors first out of the gate, with procedures that were not yet standardized across laboratories, lack of clarity about what might be the best outcome measures and an incomplete knowledge about the heterogeneity in the disease and among the trial participants. It is our hope

  that all of the products in the pipeline come to market — if not on the basis of biomarker data, then on preliminary clinical endpoints.

- However, it is also our hope for this to be a forward looking document; and it is our belief that, with current knowledge, by exercising proper care, sponsors can demonstrate changes in a number of biomarkers, including dystrophin restoration and MRI/MRS imaging, and that this may be a way forward towards being able to issue class-wide approvals for new agents.

- Considering what has been said in the guidance, the rarity and progressive debilitating nature of Duchenne for which there are no or limited rational therapeutic options, and the stated willingness of the DMD community to accept some uncertainty in both benefit and risk, we encourage the FDA to exercise maximum flexibility when reviewing future NDA applications.

- We are gravely concerned that it might be difficult to convincingly demonstrate every drug's effect within the typical drug development timespan, given the heterogeneity in natural history of the disease, uncertainty about which outcome measures to use, and what constitutes a medically addressable population for that outcome measure. Longer studies might be necessary, but they would increase the cost of drug development and also tie patients up in studies of potentially non-efficacious drugs. Consequently, we are also worried that industry will say, "We can't do studies in this population, it is too difficult, or will be too expensive. We are going to exit the stage."

- Therefore, if the FDA — because of some lingering doubts — is unwilling to accept biomarker evidence confirming a drug's mechanism of action when that mechanism of access is key to the etiology of the disease, we would request that they consider alternative drug approval mechanisms —such as adaptive approval — which could use such pharmacodynamic biomarker to grant drugs approval, until the relationship or lack of relationship of the biomarker to clinical benefit can be determined.

Parent Project
Muscular Dystrophy
401 Hackensack Avenue, 9th Floor, Hackensack, New Jersey 07601
**t** 800.714.5437 • 201.944.9985 **f** 201.944.9987 **e** info@ParentProjectMD.org • ParentProjectMD.org
